# ADP-dependent glucokinase regulates energy metabolism via ER-localized glucose sensing

**DOI:** 10.1038/s41598-019-50566-6

**Published:** 2019-10-03

**Authors:** Roland Imle, Bei-Tzu Wang, Nicolas Stützenberger, Jana Birkenhagen, Amol Tandon, Matthias Carl, Nastassja Himmelreich, Christian Thiel, Hermann-Josef Gröne, Gernot Poschet, Mirko Völkers, Karsten Gülow, Anne Schröder, Sara Carillo, Stefan Mittermayr, Jonathan Bones, Marcin Mikołaj Kamiński, Stefan Kölker, Sven Wolfgang Sauer

**Affiliations:** 1 Division of Child Neurology and Metabolic Diseases, Centre for Child and Adolescent Medicine, University Hospital Heidelberg, Im Neuenheimer Feld 430, D-69120 Heidelberg, Germany; 2 Department of Cellular and Molecular Pathology, German Cancer Research Center, Heidelberg, Germany; 3 Centre for Organismal Studies (COS), Heidelberg University, Heidelberg, Germany; 4 German Centre for Cardiovascular Research (DZHK), partner site Heidelberg/Mannheim, Heidelberg, Germany; 5 Department of Cardiology, Heidelberg University, Heidelberg, Germany; 6 German Cancer Research Center, 69120 Heidelberg, Germany; 7 University Hospital Regensburg, Internal Medicine I, Regensburg, Germany; 8 Characterisation and Comparability Laboratory, The National Institute for Bioprocessing Research and Training (NIBRT), Foster Avenue, Mount Merrion, Blackrock, Co. Dublin, A94 X099 Ireland; 9 School of Chemical and Bioprocess Engineering, University College Dublin, Belfield, Dublin 4, D04 V1W8 Ireland; 10 Department of Immunology, St. Jude Children’s Research Hospital, 262 Danny Thomas Place, Memphis, Tennessee 38105 USA; 11 Heidelberg University, Medical Faculty Mannheim, Department of Cell and Molecular Biology, 68167 Mannheim, Germany; 12 University of Trento, Center for Integrative Biology (CIBIO), Laboratory for Translational Neurogenetics, 38123 Trento, Italy; 13 Pediatric Soft Tissue Sarcoma Research Group, Hopp Children’s Cancer Center Heidelberg (KiTZ), German Cancer Research Center (DKFZ), Heidelberg, Germany; 14 Faculty of Biosciences, Heidelberg University, Heidelberg, Germany; 15 Division of Pediatric Surgery, Department of General, Visceral and Transplantation Surgery, University Hospital Heidelberg, Heidelberg, Germany

**Keywords:** Enzyme mechanisms, Glycobiology

## Abstract

Modulation of energy metabolism to a highly glycolytic phenotype, i.e. Warburg effect, is a common phenotype of cancer and activated immune cells allowing increased biomass-production for proliferation and cell division. Endoplasmic reticulum (ER)-localized ADP-dependent glucokinase (ADPGK) has been shown to play a critical role in T cell receptor activation-induced remodeling of energy metabolism, however the underlying mechanisms remain unclear. Therefore, we established and characterized *in vitro* and *in vivo* models for ADPGK-deficiency using Jurkat T cells and zebrafish. Upon activation, ADPGK knockout Jurkat T cells displayed increased cell death and ER stress. The increase in cell death resulted from a metabolic catastrophe and knockout cells displayed severely disturbed energy metabolism hindering induction of Warburg phenotype. ADPGK knockdown in zebrafish embryos led to short, dorsalized body axis induced by elevated apoptosis. ADPGK hypomorphic zebrafish further displayed dysfunctional glucose metabolism. In both model systems loss of ADPGK function led to defective N- and O-glycosylation. Overall, our data illustrate that ADPGK is part of a glucose sensing system in the ER modulating metabolism via regulation of N- and O-glycosylation.

## Introduction

ADP-dependent glucokinase (ADPGK) has first been described 1994 in hyperthermophilic archaea as a novel glucose-phosphorylating enzyme dependent on ADP (adenosine diphosphate) instead of ATP (adenosine triphosphate)^1^. It was laterally transferred to eukaryotes prior to insects^2^, though an evolutionary advantage remains elusive due to hexokinases 1–4^3,4^. Despite it’s catalytic function, this monomeric protein of about 54 kDa in size is structurally rather related to ribokinases and relies on magnesium as a cofactor^4^. It has a low Km of about 0.09 and lacks end-product inhibition. Other remarkable features are its temperature optimum at about 42° and an acidic pH-optimum of about 6^5^. It has been speculated that using ADP instead of ATP is an advantage under nutrient deprived and anoxic conditions such as those found in cancer cells suggesting a role in the “Warburg Metabolism”^4^. Highest ADPGK expression is found in immune cells of both myeloid and lymphoid lineages^5^. Upon stimulation, immune cells typically show a Warburg-like remodeling of metabolism^6^. In a previous study, we have shown that activation of Jurkat and primary human T cells leads to downregulation of mitochondrial respiration in concert with upregulation and deviation of glycolytic flux towards the glycerol-3-phosphate-dehydrogenase-shuttle, resulting in the release of a mitochondrial ROS (reactive oxygen species) signal and steering NFκB-dependent gene expression^5^. This metabolic shift coincided with increased ADPGK activity and expression of NFκB target genes like IL(interleukin)-2 or IL-8 were strongly dependent on ADPGK activity^5^. The precise mechanism of ADPGK in the activation-induced remodeling of metabolism has remained unclear. An eight-fold lower catalytic rate of ADPGK compared to classical hexokinases questions a quantitative contribution to the cellular glucose-6-phosphate pool. Considering its localization at the endoplasmic reticulum (ER), we hypothesized that ADPGK site-specifically provides glucose-6-phosphate for O-GlcNAc modifications or complex N / O-glycosylation, both being essential for T cell activation^7,8^. Our current study aims to pinpoint the role of ADPGK in metabolism. To this end, we established and characterized Jurkat T lymphocytes with CRISPR/Cas9-mutated ADPGK. We further tested the *in vivo* relevance of our findings by exploring the phenotype of ADPGK knockdown in zebrafish.

## Results

### Subcellular localization of ADPGK

**Figure 1:**
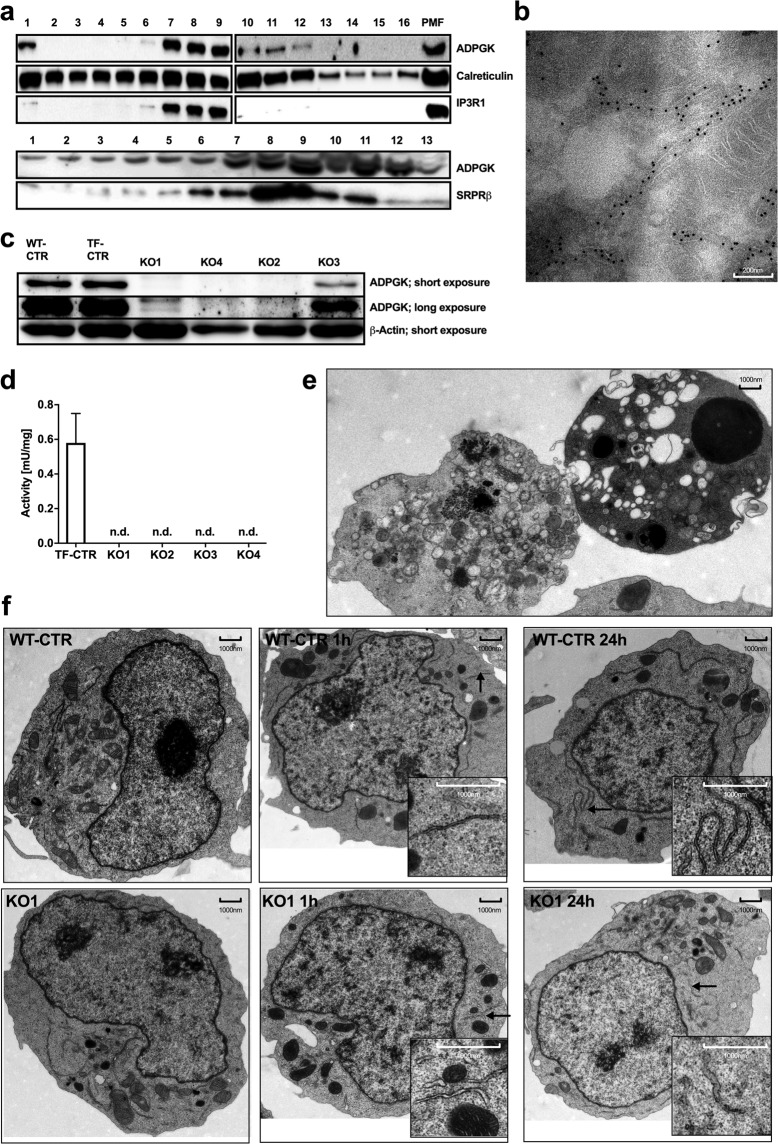
ADPGK is localized in ER lumen and important for ER biogenesis. **(a)** Representative immunoblots of density gradient-enriched ER fractions from Jurkat T cells, stained for ADPGK and different ER-markers (IP3R-1, Inositol-1,4,5-triphosphate receptor; SRPRβ, signal recognition particle receptor subunit β; PMF, post-mitochondrial fraction). N = 4 independent experiments. **(b)** Representative electron micrograph of ADPGK-GFP expressing HEK cells, stained with gold particle-labeled anti-GFP antibodies. **(c)** Representative immunoblots of ADPGK protein in Jurkat T cell knockout using β-Actin as a loading control. N = 5 independent experiments. **(d)** ADPGK activity assays in KO clones normalized to protein content. N = 7 independent experiments. **(e)** Electron micrographs of KO1 cells stimulated with PMA (10 ng/ml) and Ionomycin (10 µM) for 24 h. Dying cells show features of autophagy (left) and apoptosis (right). **(f)** Electron micrographs of KO1 and WT-CTR cells stimulated with PMA (10 ng/ml) and Ionomycin (10 µM) for 0 h, 1 h, and 24 h. Stimulation results in extended ER networks in control cells and short, dilated ER structures in KO1 cells. Black arrows indicate magnified structures. All images of blots represent cropped blots of appropriate protein size. For full length blots see Supplemental Fig. 3.

First, we studied the localization of ADPGK in the ER. Using density gradient enriched ER fractions prepared via ultracentrifugation, we examined co-localization of ADPGK with different ER marker proteins. We found that ADPGK co-localizes with ER markers calreticulin and IP3R1 (inositol-3-phosphate-receptor) as well as with the rough ER marker SRPRβ (signal recognition particle receptor) (Fig. 1a). The first 21 amino acids of the ADPGK precursor protein is the ER-targeting sequence, whereas a highly hydrophobic amino acid stretch at position 80–100 aa was predicted to be a membrane spanning region^5^, suggesting an active site protruding towards the cytosol. X-ray resolution of ADPGK structure however identified aa 72–89 as part of an amphipathic helix forming the glucose-binding site^9^. This observation indicated that APDGK is a soluble protein in the ER lumen while it cannot be excluded that the hydrophobic stretch could partly also mediate a degree of membrane association. We further gained evidence for ER-luminal localization of ADPGK in electron micrographs of HEK (human embryonic kidney) cells expressing ADPGK with a c-terminal Turbo-GFP(green fluorescent protein)-tag and stained with gold-labeled anti-GFP antibodies, which appeared to be localized within the ER lumen (Fig. 1b).

### Generation of ADPGK-deficient Jurkat T cell clones

**Figure 2:**
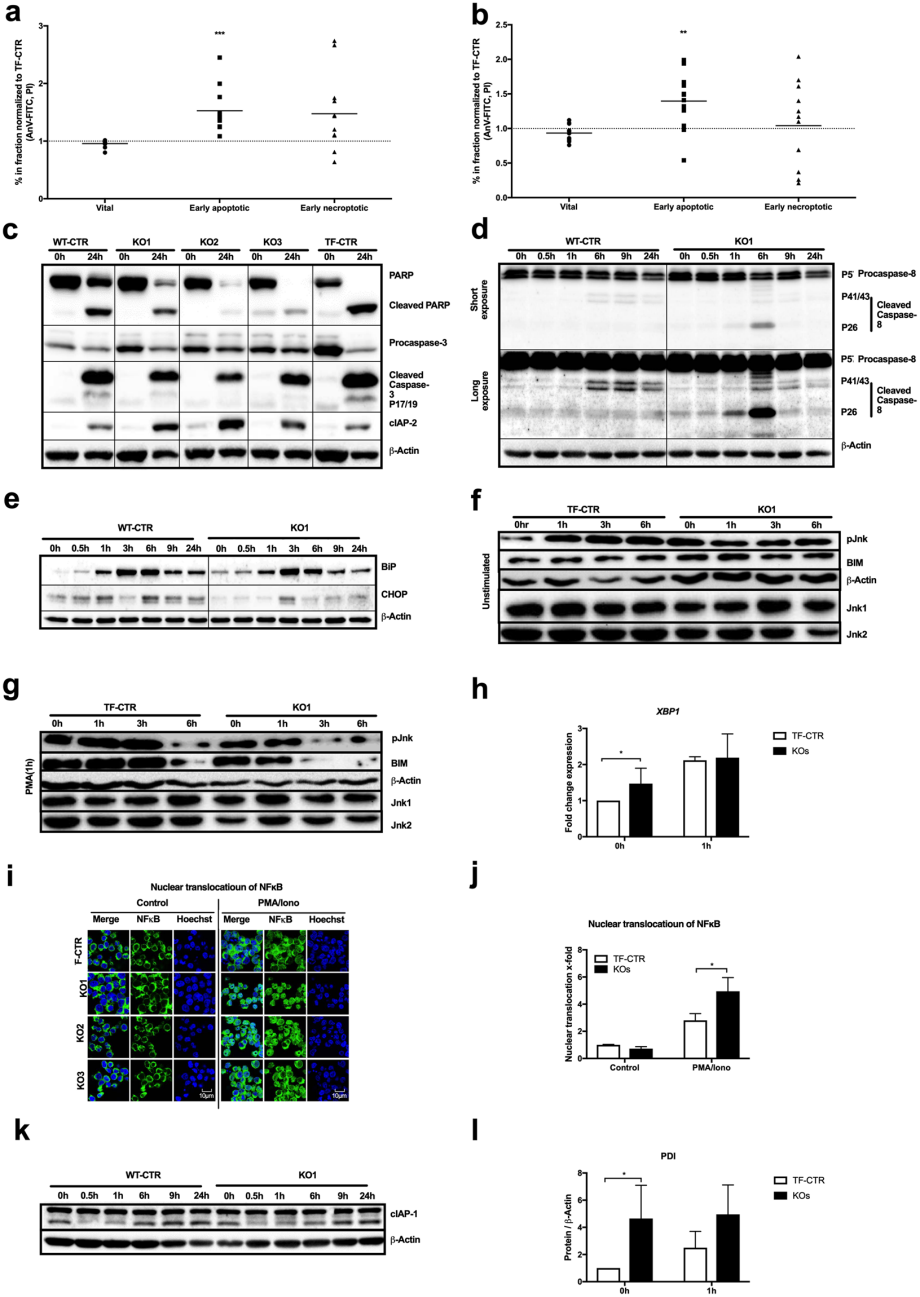
ADPGK knockout leads to apoptosis and ER stress. **(a - c)** Cytometric cell death analysis using propidium iodide (PI) and annexin V (AnV) staining in control and ADPGK KO cells (**a**) without stimulation and treated with **(b)** PMA/Ionomycin for 24 h. Individual ratios (dots) and median (line) are shown. N = 4 independent experiments; vital (AnV-/PI-), early apoptotic (AnV+/PI−), early necroptotic (AV−/PI+). **(c)** Representative immunoblot analysis of PARP-cleavage, caspase 3-cleavage and cIAP2 expression under basal conditions and after 24 h stimulation with PMA/Iono. **(d)** Expression and cleavage of caspase 8 and **(e)** expression of the ER stress marker proteins CHOP and BiP in control and KO1 cells upon different activation periods with PMA/Iono. **(f,g)** Immunoblotting for phospho-Jnk and Bim expression upon addition of the protein synthesis inhibitor cycloheximide (20 µg/ml) for 0, 1, 3 and 6 hours (**g**) without and with (**h**) PMA pre-stimulation (1 h). For normalization β-actin and for baseline Jnk-expression Jnk1/2 are shown. **(h)** RT-qPCR-analysis of spliced XBP1 with and without 1 h PMA/Iono stimulation. N = 3 independent experiments. **(i,j)** Nuclear translocation of NFκB upon PMA/Iono stimulation shown as **(i)** representative confocal single-plane micrographs stained for NfkB and nuclear counterstaining with Hoechst dye alongside **(j)** quantification plot. N = 3 independent experiments. **(k)** Immunoblots of cIAP-1 in control and KO1 cells upon different periods of stimulation with PMA/Iono. **(l)** Immunoblot analysis of PDI expression in control and ADPGK KO cells with and without 1 h PMA stimulation. All immunoblots are representative images of N = 3 independent experiments. If not stated otherwise, mean of CTRs (TF-CTR and WT-CTR) and KOs (KO1, KO2, KO3) are shown. In all experiments stimulation was induced as described in the text using 10 ng/mL PMA and 10 µM Ionomycin. * p < 0.05, ** p < 0.01, ***p < 0.001. All images of blots represent cropped blots of appropriate protein size. For full length blots see Supplemental Fig. 3.

We generated APDGK-deficient Jurkat T cells via CRISPR/Cas (clustered regularly interspread palindromic repeats) 9 technology and decided to target exons 2 and exon 4 for this approach. While exon 2 is the functionally most relevant site also responsible for glucose binding, mutations in exon 4 will impact correct protein folding. After single cell sorting we acquired three clones with mutations in exon 2 (KO1, KO2, KO4) and one with a mutation in Exon 4 (KO3). KO1, KO2, KO4 displayed a complete loss and KO3 a residual protein content in immunoblots (Fig. 1c). In all KO Jurkat T cells (KO cells) ADPGK activity was not detectable (Fig. 1d). Next, we decided to compare control and ADPGK-mutated cells under resting and stimulatory conditions. To this end we applied two chemical compounds to mimic T-cell receptor (TCR) stimulation: PMA (phorbol 12-myristate 13-acetate, mimicking diacelyglycerol/DAG, leading to PKC/protein kinase C activation) and Iono (Ionomycin mimicking IP3/Inositol-1,4,5-triphosphate, triggering ER-calcium release and subsequent NFAT (Nuclear factor of activated T cells)). Later experiments partly only made use of PMA since this is already sufficient to activate ADPGK in T cells. Stimulation of KO1 and control cells for 1 h and 24 h led to clear morphological changes in electron microscopy, particularly apoptotic features such as membrane blebbing, disintegration of organelles and chromatin condensation. PMA/Iono stimulation for 24 hours induces activation induced cell death (AICD). Indeed, we found AICD (measured via cytometric Annexin V and periodide staining), but markedly higher in KO1 cells with dying cells showing features of apoptosis and autophagy (Fig. 1e; detailed analysis Fig. 2a–c). Flag-tagged ADPGK-overexpressing Jurkat T cells that were generated previously showed reduced AICD within the same experimental setting^5^ (Suppl. Fig. 1a). We proceeded with analyzing mitochondrial and ER morphology. After PMA/Iono 1 h stimulation mitochondrial area, length, and width measured in histographs decreased in both cell lines, as shown by Röth *et al*.^10^. Upon 24 h stimulation, only in KO1 cells mitochondrial area and length further decreased (Suppl. Fig. 1b–d), suggesting mitochondrial dysfunction. Control cells responded to PMA/Iono stimulation by elongation of ER structures, showcasing physiological unfolded protein response (UPR) activation and ER biogenesis. In KO1 cells stimulation resulted in dilated, short ER structures suggesting pathophysiological ER stress, which was most prominent 24 hours after stimulation (Fig. 1f).

### ADPGK-deficiency, ER stress and apoptosis

We first followed up on the increase of cell death in KO1 cells and performed FACS (fluorescent-activated cell sorting) analysis of control and KO cells (KO1, 2, 3) stimulated with PMA/Iono for 24 h, labeled with annexin V and propidium iodide (Fig. 2a–c; representative contour plots Suppl. Fig. 1e). Even untreated KO cells showed increased apoptosis compared to controls (Fig. 2a). We found apoptosis induction in all cell lines after 24 h PMA/Iono stimulation indicated by annexin V positive cells (Fig. 2b), but KO cells responded with increased apoptosis compared to controls. Cell death was blocked by the pan caspase inhibitor Z-VAD (carbobenzoxy-valyl-alanyl-aspartyl-[O-methyl]- fluoromethylketone) (25 µM) in all tested cell lines (Suppl. Fig. 1f,g). Stimulation of control and KO cells with PMA/Iono resulted in the expected Caspase 3 and PARP (Poly(ADP-ribose) polymerase) cleavage in immunoblotting. Surprisingly, levels of cleaved Caspase 3 were decreased and PARP cleavage was unchanged in KO cells compared to stimulated controls (Fig. 2c), whereas kinetics of their cleavage did not differ (Suppl. Fig. 1h). Overall, PARP protein levels strongly decreased in KO cells (Fig. 2c, Suppl. Fig. 1h) suggesting PARP degradation by a second, concurrent cell death mechanism. We next analyzed Caspase 8 cleavage showing a transient increase of 8 cleavage after 6 h PMA/Iono stimulation in KO cells, but no difference between controls and KO cells was detected at later time points of activation (Fig. 2d).

Electron micrographs of activated KO1 suggested increased ER stress. UPR and ER stress can induce bothsurvival or apoptosis^11^. T cell stimulation strongly activates adaptive UPR for protein translation and secretion. Indeed, we found increased expression of the ER stress markers BiP (binding immunoglobulin protein) and CHOP (C/EBP homologous protein), peaking after 3 h–6 h of PMA/Iono stimulation in control and KO cells (Fig. 2e). KO cells showed increased basal (0 h) BiP (Fig. 2e) and protein disulfide isomerases (PDI, Fig. 2l) expression, indicative of chronic ER stress. Cell death induction by ER stress can occur via Caspase 3 or CHOP activation, both being diminished in KO cells (Fig. 2c,f), as well as JNK (c-Jun-N-terminal kinase)-mediated BIM stabilization^11^. In addition, PMA-induced ROS production in T cells can lead to MKP-7 (mitogen-activated protein kinase phosphatase) inactivation and prolonged Jnk phosphorylation, leading to Bim (BCL-2 interacting mediator of cell death) stabilization and cell death^12^. We compared stability of JNK phosphorylation and Bim in control and KO1 cells after 1 h PMA stimulation and under addition of cycloheximide (CHX; Fig. 2f,g). Control cells displayed the expected increase in JNK phosphorylation and Bim stabilization upon PMA treatment. In KO1 cells lifetime of p(phospho)JNK and Bim upon activation was decreased (Fig. 2h), virtually excluding this pathway as underlying mechanism of increased AICD in KO cells. Interestingly, we found increased XBP1 (X-box binding protein 1) splicing in KO cells under basal conditions, suggesting an ER stress-mediated pro-survival signal (Fig. 2h). An important mediator of this signal is NFκB (Nuclear factor of kappa-B-light-chain-enhancer of activated B cells)^13^. Indeed, nuclear translocation of NFκB upon 1 h PMA/Iono treatment was enhanced in all KO cells compared to controls (Fig. 2i,j). This increase of nuclear NFκB translocation could also result from an exaggerated activation of T cell signaling. Using an antibody against c-JUN (cellular Jun), we found similar nuclear translocation of AP-1 (activator protein 1) in control and KO cells upon 1 h PMA/Iono treatment (Suppl. Fig. 1i) linking enhanced NFκB-activation in KO cells to increased ER stress. Inhibitor of apoptosis proteins (IAPs) are important NFκB-target genes and inhibit caspase activity with cIAP 1 / 2 targeting Caspase 3^14,15^. Accordingly, we found increased expression of cIAP2 in all KO clones compared to control cells after 24 h PMA/Iono stimulation explaining reduced cleavage of Caspase 3 in these cells (Fig. 2c; kinetics, Suppl. Fig. 1h) while cIAP1 expression remained unchanged (Fig. 2k).

In summary, KO cells display enhanced activation of ER stress response that induces an adaptive but not apoptotic response. However, upon activation only control cells had the capacity to increase activation of the ER stress response, i.e. XBP1 and PDI (Fig. 2h,l) being in line with the failure of KO cells to activation-dependently remodel ER morphology.

### ADPGK-deficiency induces an anti-Warburg phenotype leading to a metabolic catastrophe

**Figure 3:**
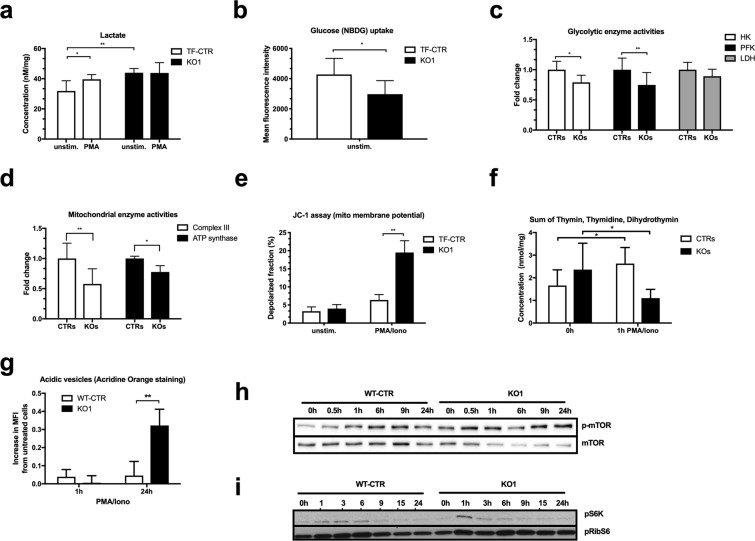
ADPGK regulates energy metabolism. (**a)** Lactate concentrations (n = 4 independent experiments) and **(b)** glucose uptake (in 1 hour; n = 3 independent experiments) in control and KO1 cells. **(c)** Changes of enzymatic activities of Hexokinase (HK), Phosphofructokinase (PFK) and Lactate dehydrogenase (LDH) in ADPGK KO clones compared to control cells. N = 3 independent experiments. **(d)** Changes of respiratory chain complex III and ATP synthase activities in ADPGK KO clones compared to control cells. N = 3 independent experiments. **(e)** Mitochondrial membrane potential measured in control and ADPGK KO cells after 24 h stimulation with PMA/Iono using JC-1 dye. N = 3 independent experiments. **(f)** Sum of thymidine degradation intermediates (thymin, thymidine, dihydrothymine) in lysates of control and ADPGK KO cells treated with PMA/Iono for 0 h or 1 h. N = 3 independent experiments. **(g)** Acridine Orange staining of control and KO1 cells upon 1 h and 24 h PMA/Iono stimulation. N = 3 independent experiments. **(h)** Representative immunoblots for mTOR phosphorylation after different PMA/Iono activation periods of control and KO1 cells. N = 3 independent experiments. **(i)** Immunoblots of pS6K and pRibS6 after different PMA/Iono activation periods in control and KO1 cells. In all experiments stimulation was induced as described in the text using 10 ng/mL PMA and 10 µM Ionomycin. *p < 0.05, **p < 0.01. All images of blots represent cropped blots of appropriate protein size. For full length blots see Supplemental Fig. 3.

We proceeded to study the metabolic phenotype of KO cells. In particular, we asked whether ADPGK-depletion would impair induction of the Warburg phenotype hallmarks glucose uptake, hexokinase activity, and lactate production. While KO cells showed higher basal lactate concentrations indicative of metabolic stress, only control cells increased lactate production upon 1 h PMA stimulation (Fig. 3a), measured photometricaly. In line with our previous finding of increased glucose uptake in ADPGK-overexpressing cells^5^, ADPGK-knockout resulted in a reduced uptake of fluorescently labeled glucose (NBDG) (Fig. 3b). ATP content measured via HPLC (high pressure liquid chromatography) with and without stimulation did not differ between control and KO cells (Suppl. Table 2). Next, we monitored activities of glycolytic pacemaker enzymes and mitochondrial respiratory chain in coupled enzyme assays. KO cells displayed reduced activities of hexokinase and phosphofructokinase whereas pyruvate kinase and lactate dehydrogenase activities were unchanged (Fig. 3c). Analysis of respiratory chain complexes revealed decreased activities of complex III and ATP synthase in KO cells (Fig. 3d). Using the voltage-dependent dye JC-1 we observed strong depolarization of mitochondrial membrane potential in KO cells after 24 hours PMA/Iono activation, functionally reflecting mitochondrial dysfunction (Fig. 3e). Overall, ADPGK deficiency results in an anti-Warburg phenotype and respiratory chain dysfunction.

Next, we performed targeted metabolic analysis of pathways that are linked to glucose 6-phosphate generation via HPLC. Analysis of nucleoside mono-, di-, and triphosphates, NAD/NADH, NADP/NADPH (nicotinamide adenine di-nucleotide-(phosphate)), purine and uridine metabolites, and the Krebs cycle markers acetyl-CoA, succinyl-CoA, and malonyl-CoA did not reveal any major changes KO cells (Suppl. Table 2). Interestingly, control cells responded to PMA/Iono stimulation (1 h) with increased thymidine catabolism (Fig. 3f), which is in line with previous reports^16^. Thymidine is used as alternative energy supply under glucose deprived conditions^17^, being an anaplerotic precursor for glycolysis and Krebs cycle. Intermediates of thymidine catabolism were strongly depleted in activated KO cells (Fig. 3f) suggesting a drain of metabolic precursors. Further highlighting impaired metabolism, acridine orange staining of KO cells revealed increased content of acidic vesicles compared to controls after 24 h PMA/Iono activation (Fig. 3g) indicating increased autophagy as seen in electron microscopy (Fig. 1e). Supporting the notion of activation-induced remodeling of energy metabolism, control cells displayed enhanced phosphorylation of mTOR (mechanistic target of rapamycin) (Fig. 3h) as well as of S6 kinase (pS6K) and its substrate ribosomal protein S6 (pRibS6), peaking upon 6 h PMA/Iono stimulation (Fig. 3i). Stimulation of KO cells resulted in activation of mTOR signaling peaking 1 h after PMA/Iono stimulation. Already after 3 h pS6K strongly decreased indicating inactivation of mTOR signaling and activation of autophagy (Fig. 3i).

Overall, our metabolic findings suggest that the increase of cell death in KO cells upon stimulation compared to control cells, i.e. the second cell death hit amplifying the classical AICD pathway, is induced by a so-called metabolic catastrophe^18^, the inability to produce sufficient energy for survival.

### ADPGK-deficiency and glycosylation

**Figure 4:**
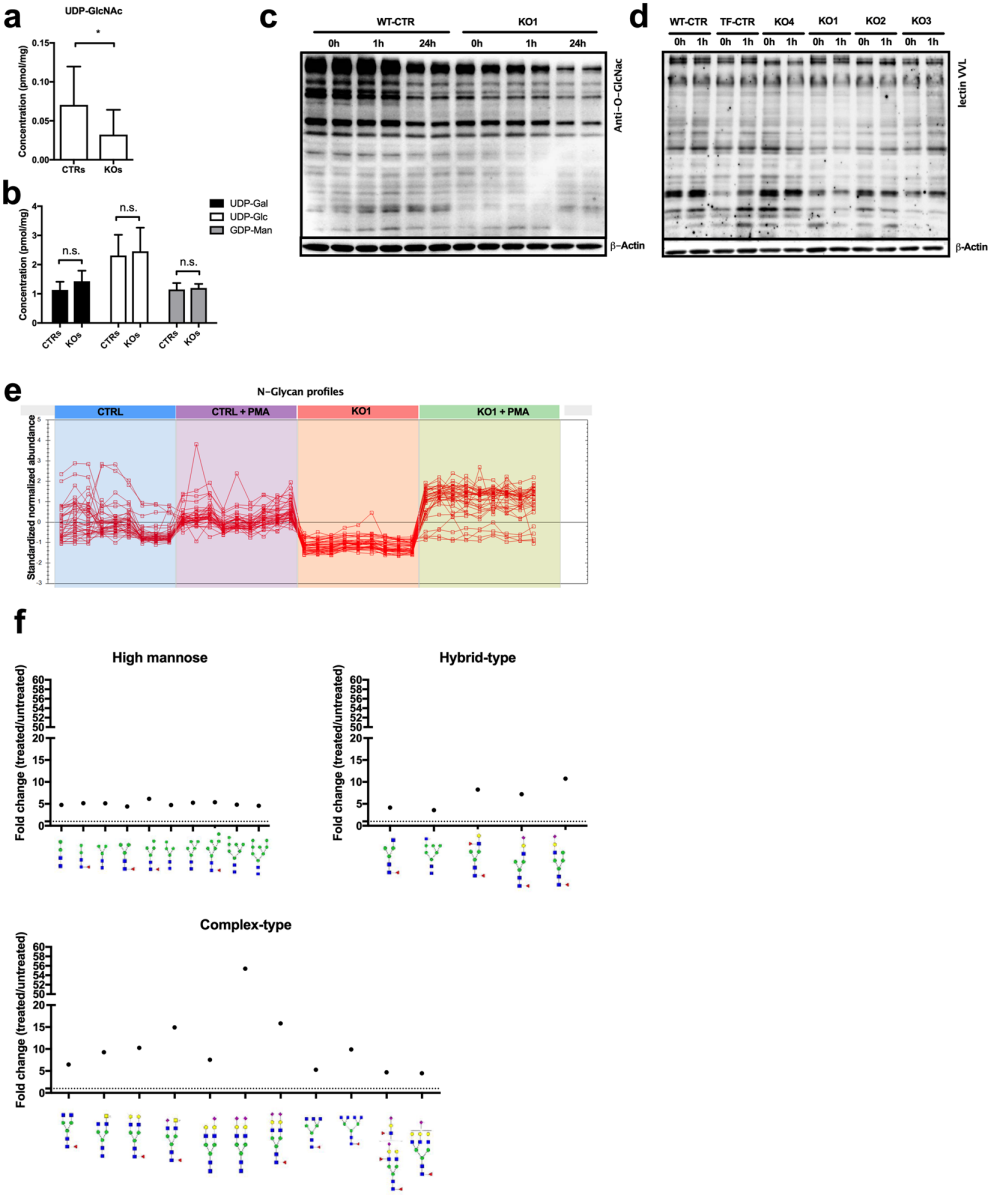
ADPGK finetunes glucose flux into O- and N-glycosylation. **(a,b)** Sugar precursors (UDP-GlcNAc, UDP-Gal, UDP-Glc, GDP-Man) of glycosylation. N = 4 independent experiments. **(c, d)** Representative immunoblots for GlcNAc-residues via monoclonal antibody against **(c)** O-GlcNAc and **(d)** the lectin VVL in lysates of control and ADPGK KO cells stimulated with PMA for different time periods. N = 3 independent experiments. **(e)** Abundance profile of changes in N-glycans in control and KO1 cells upon 24 h PMA/Iono stimulation and **(f)** scheme of changes in high mannose glycans, complex- and hybrid-type glycans in KO1 cells upon 24 h PMA/Iono activation analyzed via HILIC-FLR-MS. Mean of CTRs (TF-CTR and WT-CTR) and KOs (KO1, KO2, KO3) are shown. In all experiments stimulation was induced as described in the text using 10 ng/mL PMA and 10 µM Ionomycin. *p < 0.05. All images of blots represent cropped blots of appropriate protein size. For full length blots see Supplemental Fig. 3.

We next followed up on the notion that ADPGK provides precursors for glycosylation. Indeed, uridine diphosphate N-acetylglucosamine (UDP*-*GlcNAc) levels measured via HPLC were decreased in KO cells (Fig. 4a), whereas the remaining precursors (U(uridine)DP-galactose, UDP-glucose, G(guanosin)DP-mannose) remained unaffected (Fig. 4b). O-GlcNacylation is a crucial signaling mechanism for T cell-activation^19^. Using a GlcNac-specific antibody, we studied activation-induced (PMA) changes in O-GlcNacylation in a time-dependent manner (Fig. 4c). Control cells and KO cells alike showed a reduction of GlcNacylation after 1 hr PMA-activation, which was even more pronounced 24hrs after activation. However, signal intensity in KO cells was markedly lower over all tested conditions (no stimulation, 1 hr, 24 hrs). We confirmed these findings in co-stimulation experiment using PMA/Iono (Suppl. Fig. 1j) as well as in the remaining KO cell lines (Fig. 4d). A tendency of small molecular proteins to increase GlcNac-signal intensity in control cells could also not be observed in KO cells (Fig. 4c,d). Overall, we observed diminished UDP-GlcNac levels in homogenates of whole KO cells (Fig. 4a) as well as a general decrease of protein O*-*GlcNAcylation signal in KO compared to control cells, particularly upon activation (Fig. 4d). This suggests a role of ADPGK in glycosylation, possibly via glucose-handling or -sensing at the ER.

Next, we analyzed the activation-induced changes of N-glycosylation by mass spectrometry. Without stimulation, the overall N-glycan content in KO1 cells was decreased compared to control cells. Upon 24h PMA stimulation, both cell lines up-regulated N-glycan formation (Fig. 4e). Studying the underlying glycan structures, we found accumulation of complex, bi-antennary structures in KO cells, whereas the numbers of tri- or quaternary-branched N-glycans remained even below control cells without stimulation (Fig. 4f, Suppl. Table 3). This finding indicates that in KO cells especially the branching of complex N-glycans in the Golgi apparatus is impaired.

### *Adpgk* knockdown in zebrafish leads to aberrant body axis development

**Figure 5:**
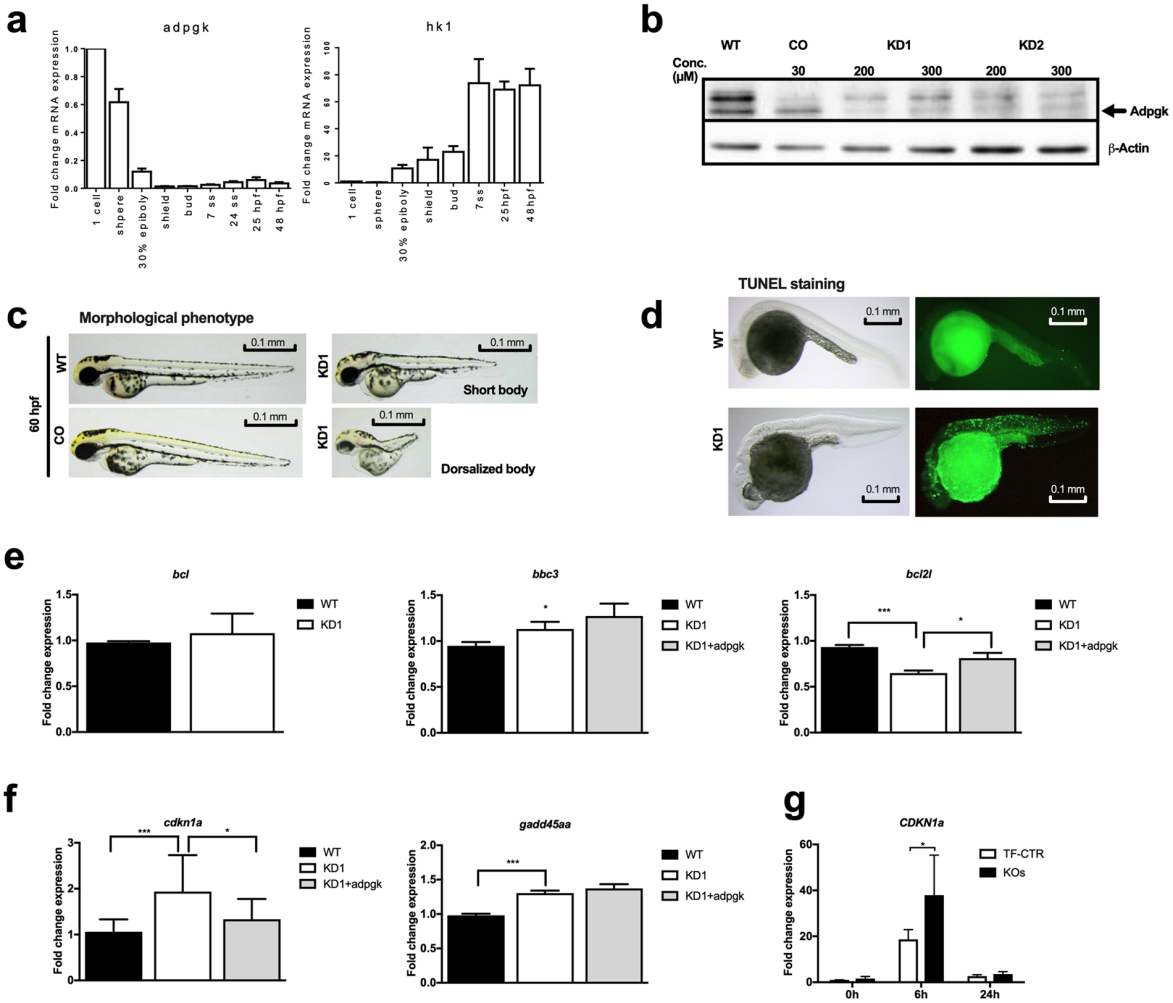
Knockdown of Adpgk in embryonic zebrafish results in aberrant body axis development and cell death. **(a)** RT-qPCR analysis of *adpgk* and *hexokinase 1 (hk1)* expression in wild type embryos collected at different developmental stages. N = 4 independent experiments. **(b)** Immunoblotting of Adpgk after blocking correct splicing (KD1) or translation (KD2) of *adpgk* mRNA by morpholino (MO) technology in zebrafish embryos, compared to wild type and control MO treated animals. Representative images, N = 3 independent experiments. **(c)** Representative images of aberrant body axis development in KD1 zebrafish ranging from shortened body to strong dorsalization (stage 60 hpf; lateral views with anterior to the left). Hypomorphic zebrafish are compared to age matched wild type and control MO treated animals. **(d)** Representative images of TUNEL staining in wild type and KD1 embryos (stage 22 hpf; lateral views with anterior to the left). **(e)** RT-qPCR analysis of *bcl, bbc3* and *bcl2l* expression in wild type and KD1 embryos as wells as KD1 embryos rescued with *adpgk* mRNA (stage 8 hpf). N = 5 independent experiments. **(f)** RT-qPCR analysis of the cell cycle checkpoint genes *cdkn1a* and *gadd45aa* in wild type and KD1 embryos as well as KD1 embryos rescued with *adpgk* mRNA (stage 8 hpf). N = 5 independent experiments. **(i)** RT-qPCR analysis of *CDKN1A* expression in control and ADPGK-deficient Jurkat T cells after different periods of stimulation with and without PMA. N = 3 independent experiments. * p < 0.05, ** p < 0.01, ***p < 0.005. All images of blots represent cropped blots of appropriate protein size. For full length blots see Supplemental Fig. 3.

Next, we tested whether *adpgk* regulates energy metabolism *in vivo* using zebrafish. Zebrafish *adpgk* shares 64% similarity with the human orthologue. We first screened *adpgk* expression at different developmental stages by RT-qPCR (reverse transcriptase quantitative polymerase chain reaction). *adpgk* expression was highest at the very early stages suggesting it to be maternally provided. Expression decreased and remained low during further embryonic development (Fig. 5a). In contrast, Hexokinase 1 (HK1) was weakly expressed at early developmental stages and peaked at 25 hpf (hours post fertilization) (Fig. 5a). Whole mount *in situ* hybridization (WISH) showed ubiquitous *adpgk* expression in zebrafish embryos at blastula stage, becoming more defined to the dorsal side during gastrulation and notochord during segmentation stage. At pharyngula stage *adpgk* expression becomes weak yet ubiquitous (Suppl. Fig. 2a).

We used morpholino (MO) technology to block correct splicing of exon 2 (MO1; knockdown (KD) 1) and translation of *adpgk* (MO2; KD2). MO1 targets the boundary of exon 2 and intron 2, and was predicted to create a 275-nucleotide exon excision. We found expression of the corresponding truncated *adpgk* transcript (Suppl. Fig. 2b) and a more than 2 fold decrease of overall *adpgk* mRNA expression (Suppl. Fig. 2c). MO1 and MO2 resulted in diminished *adpgk* protein synthesis (Fig. 5b). Phenotypically, *adpgk* knockdown by MO1 or MO2 led to abnormal body axis development and irregularly shaped somites. The phenotypic severity was MO concentration-dependent ranging from short body axis to dorsalization of the embryo (Fig. 5c; Suppl. Fig. 2d,e,h). Overexpression of wild-type human or zebrafish *adpgk* mRNA similarly resulted in dorsalized body phenotype (Suppl. Fig. 2f). Expressing wild-type zebrafish or human *adpgk* mRNA (messenger ribonucleic acid) in KD1 or KD2 hypomorphs rescued the short body axis phenotype (Suppl. Fig. 2d,e). The finding that human mRNA rescued *adpgk* hypomorphs suggests a conserved function over species boundaries.

### *Adpgk* hypomorphs show increased apoptosis and activated cell cycle checkpoints

We continued to screen the major embryonic signaling pathways (wnt (wingless-related integration site), sonic hedgehog, notch) via PCR to detect aberrant gene expression. KD1 embryos showed consistent changes in expression of genes important for cell cycle (*cdkn1a (cyclin dependent kinase inhibitor 1a)*, *gadd45a (growth arrest and deoxyribonucleic acid damage inducible)*) and apoptosis (*bbc3 (bcl-2binding component 3)*, *bcl2l(b-cell-lymphoma 2)*). Hypothesizing an imbalance of coordinated apoptosis during embryogenesis as a cause for the observed developmental phenotype of *adpgk* hypomorphs, we next investigated cell death in embryos at 24 hpf by Acridine orange (Suppl. Fig. 2g) and TUNEL (terminal deoxynucleotidyl transferase dUTP nick end labeling) staining (Fig. 5d). Both experiments revealed increased numbers of apoptotic cells in KD1 embryos. In line with increased apoptosis, albeit unchanged *bcl* expression, KD1 embryos showed elevated expression of the pro-apoptotic factor *bbc3* and reduced expression of the anti-apoptotic gene *bcl2l*. Reduced *bcl2l* expression was rescued by injecting zebrafish *adpgk* mRNA (Fig. 5e). Increased expression of *cdkn1a* and *gadd45aa* in KD1 embryos suggested additional activation of cell cycle checkpoints. Expression of *cdkn1a* could be normalized when supplying zebrafish *adpgk* mRNA (Fig. 5f). P53 up-regulation is one of the most common triggers of cdkn1a^20^. However, MO-mediated suppression of p53 (tumor protein 53) expression in KD1 or KD2 embryos even exacerbated cell death (Suppl. Fig. 2h), yet confirming that cell death is not due to p53-mediated MO toxicity. Of note, P53 and CDKN1A act protective under increased metabolic stress^21^. We proceeded to analyze *CDKN1A* and *GADD45* expression in Jurkat T cells. *GADD45* was only weakly expressed, but expression of *CDKN1A* was induced by PMA stimulation and higher in KO cells compared to control cells (Fig. 5g). Accordingly, cell cycle analysis revealed extension of S phase in all KO cells after 12 h and 24 h PMA/Iono stimulation (Suppl. Fig. 1k–m). Summarizing, *adpgk* knockdown in zebrafish leads to increased apoptosis and upregulation of cell cycle arrest genes.

### Disturbed glucose homeostasis and glycosylation in zebrafish embryos with Adpgk deficiency

**Figure 6:**
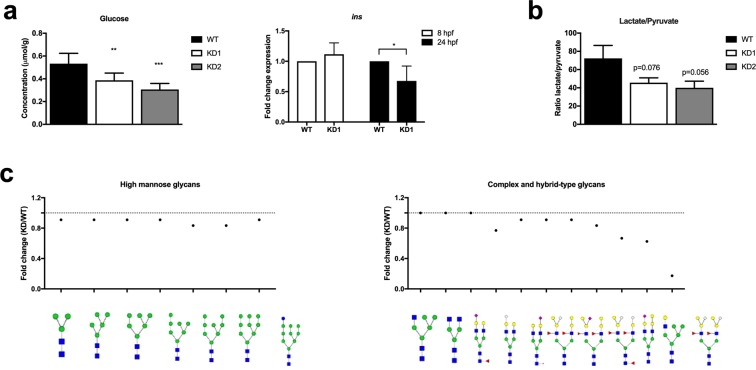
Adpgk regulates glucose metabolism in zebrafish embryos. (**a)** Quantification of whole body glucose content and insulin expression in wild type, KD1, and KD2 embryos. N = 6 independent experiments, stage 22 hpf. **(b)** Ratio of lactate to pyruvate in wild type, KD1, and KD2 embryos. N = 3 independent experiments, stage 22 hpf. **(c)** Scheme of changes in high mannose glycans, complex- and hybrid-type glycans in KD1 compared to control embryos (stage 22 hpf) analyzed by HILIC-FLR-MS. * p < 0.05, ** p < 0.01, ***p < 0.005.

We next investigated whole body glucose homeostasis after *adpgk* knockdown. KD1 and KD2 embryos had decreased free glucose levels normalized to protein content (measured photometrically) and *insulin* expression compared to WT embryos (Fig. 6a). We next assessed whether the anti-Warburg phenotype of KO Jurkat cells would be corroborated in *adpgk* hypomorphs. Indeed, *adpgk* hypomorphic zebrafish displayed reduced lactate-to-pyruvate ratio (Fig. 6b). Coupled enzyme assay analysis of glycolysis did not reveal differences between KD1 and wild type embryos (Suppl. Fig. 2i). Similar to KO cells, KD1 embryos displayed decreased electron flow from complex I to III and lower ATP synthase activity, the latter effect being a trend (Suppl. Fig. 2j).

A major finding in KO cells was aberrant O-and N-glycosylation. Immunoblots for O-GlcNAc from KD1 and KD2 embryos lysates confirmed decreased O-GlcNAcylation of proteins with a size between 26 kDa to 55 kDa compared to control embryos (Suppl. Fig. 2k). Mass spectrometric N-glycan analysis showed an overall decrease of high mannose glycans in KD1 embryos compared to wild type fish (Fig. 6c; Suppl. Table 4). Specific complex- and hybrid-type glycans were stronger diminished than it would be expected from the decrease of high mannose precursors (Suppl. Table 4). In particular, HexNAc_4_Hex_7_Fuc_2_NeuGc_2_ was decreased by 5.85 fold and GlcNAc_2_Man_5_GlcNAcGal was decreased by 1.6 fold in the KD1 embryos (Fig. 6c). This pronounced effect of *adpgk* knockdown on complex glycans in zebrafish is highly reminiscent of the N-glycan pattern found in KO cells.

## Discussion

The aim of our study was to pinpoint the role of ADPGK in metabolism. Based on our previous findings [5], we hypothesized that ADPGK regulates induction of Warburg phenotype in activated T cells. Failure of T cells to reprogram their metabolism towards an anabolic phenotype^6,22^ leads to T-cell dysfunction and cell death^23^. Indeed, activation of KO cells with PMA/Iono resulted in increased AICD. Intriguingly, this phenotype was associated with weaker activation of Caspase 3, but increased degradation of PARP, suggesting activation of an additional cell death mode. Since cell death was blocked by pan-caspase inhibitor Z-VAD, this second hit is dependent on induction of the classical AICD route. Analysis of different metabolic pathways suggests that failure to metabolically adapt to the increased bioenergetic and anaplerotic needs upon activation is the amplifier of AICD in KO cells. ADPGK deficiency resulted in severe disturbance of energy metabolism, namely (1) reduced glucose uptake (2) diminished activities of hexokinase, phosphofructokinase, and respiratory chain complexes, (3) activation-induced depletion of thymidine metabolism intermediates, and (4) enhanced activation of autophagy. In addition to impaired energy metabolism, KO cells displayed aberrant O-GlcNAcylation and N-glycosylation.

The surprising reduction of Caspase 3 cleavage in activated KO cells resulted from ER stress induced cIAP2 activity via XBP1/NFκB signaling. NFκB activation and autophagy are commonly seen as pro-survival mechanisms, yet they can context-dependently support or even induce apoptosis. The interplay of autophagy and apoptosis is still incompletely understood in mammals^24^. There is increasing evidence that autophagy can enhance apoptotic signals, especially when these signals are impaired^25^ such as it seems to be the case in ADPGK KO cells. Even NFκB can promote apoptosis under certain cancer therapies^26^, and a recent study reports on ER stress-induced autophagy and apoptosis depending on NFκB signaling^27^.

*adpgk* hypomorphic zebrafish corroborated the *in vitro* findings. Treated embryos showed a dorsalization of body axis, which was associated with increased apoptosis and activation of cell cycle check points. Metabolically, we observed altered glucose handling characterized by depletion of free glucose levels, decreased insulin expression and reduced lactate production. ADPGK hypomorphic embryos further displayed respiratory chain dysfunction. Importantly, ADPGK knockdown also led to aberrant O-GlcNAcylation and N-glycosylation.

Our study provides novel evidence for a yet poorly understood role of the ER in energy sensing and glucose handling in extrahepatic tissue. Generation of glucose in hepatic gluconeogenesis and glycogenolysis is catalyzed by the ER-membrane-associated complex of glucose-6-phosphate translocase (G6PT) and glucose-6-phosphatase (G6PC1). Similar to ADPGK, this complex is found in the rough ER^28^. Mutations of G6PC1 or G6PT cause glycogen storage disease type I and result in impaired hepatic regulation of glucose homeostasis. There is increasing evidence that a complex of G6PC3/G6PT regulates glucose metabolism in immune cells. Lymphopenia and reduced activation-induced immune cell glycolysis have been described in patients with G6PT deficiency^29^. Mutations in G6PC3 or G6PT lead to severe neutropenia and agalactosylated complex type N-glycans in neutrophils^30,31^. Murine G6PC3-deficient neutrophils and macrophages display reduced uptake and metabolism of glucose^32^. The mechanism underlying regulation of energy homeostasis by the ER glucose 6-phosphate system remains speculative. It has been suggested that this system regulates glucose 6-phosphate availability in the cytosol^32^. Glucose generated by the G6PC/G6PT complex can be released from the ER via vesicular transport^33^ or GLUT transporters in the ER membrane^34^. Considering the phenotypic similarities to G6PC3/G6PT-deficient immune cells^29–31^, ADPGK could possibly be part of an ER glucose/glucose 6-phosphate system involved in retention of glucose in this compartment via phosphorylation.

Notably in the context of this hypothesis, N-glycan branching is impaired upon deficiency of ADPGK, but also G6PC3 and G6PT^30,31^. This process is mediated by N-acetylglucosaminyltransferases (GNT I-V) in the Golgi apparatus. GNTs are characterized by an intriguing Km pattern for their substrates UDP*-*GlcNAc and glycoprotein, i.e. the more complex the N-glycan the higher the Km of the responsible GNT for UDP*-*GlcNAc but the lower for the glycoprotein^35^. Strong dependence of tri- and tetra-antennary structures on high cellular UDP*-*GlcNAc concentrations works therefore as a cellular energy sensor. The reduced UPD-GlcNAc content of ADPGK KO cells can explain the strong increase of bi-antennary N-glycans upon activation. Supporting this notion, deficient formation of tri-antennary, complex N-glycans by GNT-IV knockout in mice results in accumulation of bi-antennary N-glycans and impaired glucose metabolism^36^. Mutations in the Golgi UDP-GlcNAc transporter (SLC35A3) have first been described in Holstein cattle and lead to complex vertebral malformations^37^. Severe mutations of the human orthologue similarly result in skeletal dysplasia^38^. This phenotype resembles ADPGK knockdown zebrafish with shortened body axis and irregular somites, which will give rise to vertebrae.

Overall, our study shows that ADPGK regulates cellular energy metabolism *in vitro* and *in vivo* by being part of an ER localized glucose/glucose-6-phosphate system modulating protein glycosylation.

## Materials and Methods

### Cell culture and cell stimulation

Jurkat T lymphocytes J16–145 were maintained in RPMI including Glutamine (Thermo Fisher Scientific), 10% fetal calf serum (FCS) and 1% Penicillin/Streptomycin. HEK293T cells in DMEM (Thermo Fisher Scientific) with the same supplements. CRISPR/Cas9-mediated knockout of ADPGK was performed using plasmids expressing guide-RNA under an U6-promotor and Cas9 as well as GFP under a CMV-promotor (GMA CRISPR Plasmid Sigma Aldrich). Target sites were GCTTATCGTGCGGCCAGTCCGG (Exon 2), GTCAATGCATGTGTTGATGTGG (Exon 2) and GTTCCACCAGAGTCATTGCAGG (Exon 4). As controls we used wild type Jurkat T cells (WT-CTR) and Jurkat T cells transfected with a GFP-plasmid (“Amaxa SE Cell Line 4D Nucleofector Kit”, Lonza) in parallel to KO cells (TF-CTR). Jurkat cells were transfected using “Amaxa Human T Cell Nucleofector Kit” (Lonza) according to the manufacturer’s protocol before being sorted by FACS. DNA of single cell clones was purified using “DNeasy Blood & Tissue Kit” (Qiagen) according to the manufacturer’s protocol, the respective DNA sequence amplified using a Q5® High-Fidelity DNA Polymerase (New England BioLabs), and the PCR product was sequenced at Microsynth SEQLAB (Balgach, Switzerland). For heterozygous mutations, the amplified DNA was subcloned in DH5α E. coli using Zero Blunt® TOPO® PCR- cloning kit (ThermoFisher Scientific) and at least 10 colonies were picked for sequencing. ADPGK overexpressing cells (OE) and their controls (CTRL-OE) were generated previously^5^. Stimulation of Jurkat T lymphocytes was induced by PMA (10 ng/ml) or PMA and Ionomycin (10 µM) to mimic anti-CD3 stimulation of T-cell receptors. HEK293T cells intended for EM immunostaining were transfected as previously reported^5^, using PEI (Polyethylenimine). 2.6 mg of plasmid encoding human ADPGK tagged with turboGFP on the C terminus (ADPGK-tGFP; pCMV6-AC, provided by Origene; Rockville, MD, USA) diluted in 200 ml of serum-free DMEM was mixed with PEI (0.74 ml of PEI diluted in 200 ml DMEM). After incubation for 10 min the mixture was added to the cells drop by drop, 6 hours later medium was changed. 24 hours later cells were fixed and processed for electron microscopy immunostaining.

For remaining materials and methods, see supplementary information.

### Data analysis

Statistical analysis was performed using SPSS statistics (22.0) and GraphPad Prism (7.0). If not stated otherwise in the text, data were analyzed by unpaired, two-sided t-Tests and are presented as mean ± standard deviation.

### Ethical statement

All experiments were performed in accordance with the regulations of the ethics committee of the medical faculty of the university of Heidelberg (DIN EN ISO 9001). Zebrafish housing and experiments were performed in accordance with all international and national laws and obligations as registered at the Regierungspräsidium Karlsruhe (Az. 35-9185.81/G-85/16).

## Supplementary information

Supplementary information
