# Supplementary material for: ADP-dependent glucokinase regulates energy metabolism via ER-localized glucose sensing
Source: Sci Rep. 2019 Oct 3;9:14248. doi: 10.1038/s41598-019-50566-6 (PMC6776650; doi:10.1038/s41598-019-50566-6)

ADP-dependent glucokinase regulates energy metabolism via ER-localized glucose sensing

# - Supplementary Information -

Imle Roland[1]*, Wang Bei-Tzu[1]*, Stützenberger Nicolas[1]*, Birkenhagen Jana[1], Tandon Amol[1], Carl Matthias[11,12], Himmelreich Nastassja[1], Thiel Christian[1], Gröne Hermann-Josef[2], Poschet Gernot[3], Völkers Mirko[4,5], Gülow Karsten[6,7], Schröder Anne[6], Carillo Sara[8], Mittermayr Stefan[8], Bones Jonathan[8,9], Kaminski Marcin Mikolaj[10], Kölker Stefan[1], Sauer Sven Wolfgang[1 #]
*contributed equally
#corresponding author, Sven77sauer@icloud.com, +4915226764047

1 Division of Child Neurology and Metabolic Diseases, Centre for Child and Adolescent Medicine, University Hospital Heidelberg, Im Neuenheimer Feld 430, D-69120, Heidelberg, Germany.
2 Department of Cellular and Molecular Pathology, German Cancer Research Center, Heidelberg, Germany.
3 Centre for Organismal Studies (COS), Heidelberg University, Heidelberg, Germany.
4 German Centre for Cardiovascular Research (DZHK), partner site Heidelberg/Mannheim, Heidelberg, Germany.
5 Department of Cardiology, Heidelberg University, Heidelberg, Germany.
6 German Cancer Research Center, 69120 Heidelberg, Germany.
7 University Hospital Regensburg, Internal Medicine I, Regensburg, Germany
8 Characterisation and Comparability Laboratory, The National Institute for Bioprocessing Research and Training (NIBRT), Foster Avenue, Mount Merrion, Blackrock, Co. Dublin, A94 X099, Ireland.
9 School of Chemical and Bioprocess Engineering, University College Dublin, Belfield, Dublin 4, D04 V1W8, Ireland.
10 Department of Immunology, St. Jude Children's Research Hospital, 262 Danny Thomas Place, Memphis, Tennessee 38105, USA.
11 Heidelberg University, Medical Faculty Mannheim, Department of Cell and Molecular Biology, 68167 Mannheim, Germany
12 University of Trento, Center for Integrative Biology (CIBIO), Laboratory for Translational Neurogenetics, 38123 Trento, Italy

Supplementary Material and Methods

Density gradient preparation of Endoplasmic Reticulum fractions

Preparation of ER fractions for immunoblotting was performed with the “Endoplasmic Reticulum Isolation Kit“ (Sigma Aldrich) according to the manufacturer’s protocol. In brief, cells were pelleted, washed and homogenized by passing through a 27 G needle. Centrifugation at 1,000 g for 10 minutes at 4°C removed cell debris. Supernatants were further centrifuged at 11,000 g at 4°C for 15 minutes with 11,000 g. Respective supernatants were regarded as post-mitochondrial fractions (PMF). These PMFs were further centrifuged at 100,000 g at 4°C for 1 hour. Pellets were regarded as crude microsomes, resuspended in isotonic extraction buffer and passed through a 27 G needle until homogenous. Homogenous PMFs were adjusted to 20% (w/v) Optiprep sucrose solution in a total volume of 400µl and layered in the center of a sucrose solution sandwich with 533µl of 30% at the bottom and 1067µl of 15% on top. A final unltracentrifugation step at 150,000 g for 3 hours at 4°C was carried out to separate organelles within the sucrose density gradient, which was separated into 13-16 fractions and subjected to SDS-Page and western blotting.

Rough ER was also isolated from PMFs by calcium chloride precipitation: PMFs were stirred by a magnetic spin bar while calcium was gradually added to a final concentration of 7mM, before rER was pelleted at 8,000 g for 10 minutes at 4°C.

Electron micrographs

Under basal or stimulatory conditions 5×10^7 Jurkat cells were collected and fixed with 2.5% glutaraldehyde + 1.5% Osmiumtetroxide (both dissolved in 0.05 M cacodylate buffer) for 30 minutes at 4°C. After rinsing with buffer the cells were fixed again with Osmium tetroxide for 2 hours at 4°C. Cells were dehydrated via an ascending ethanol line (50%, 80%, 96% and 100%) and then incubated with Propylenoxide/Araldite-mixture 1:1 overnight at 4°C. Cells were then incubated with fresh araldite for 4 hours and polymerized for 24 hours at 60°C.

Samples were embedded in Araldite (SERVA), contrasted with uranylacetate and lead citrate and sectioned with Ultracut 70nm (Leica; Solms, Germany). For immunostaining vs. GFP, ADPGK-turboGFP expressing HEK293T cells were used and stained with polyclonal rabbit anti-TurboGFP (Evrogen AB513) for 2 hours followed by a gold-labeled secondary antibody (GAM, AURION, 10nm) overnight. Images were acquired with a Zeiss EM910 microscope.

Cell death analysis

Cell death was determined using "FITC Annexin V Apoptosis Detection Kit" (BD Pharmingen) according to the manufacturer's protocol using a cytometer (FACSVerse, Becton Dickinson). The assay distinguishes between AnnexinV-FITC-only-positive cells (detecting phosphatidylserine residues flipped to the outside of the plasma membrane, indicative of early apoptosis), periodide/PI-only positive cells (indicative plasma membrane permeability in advanced stages of cell death/necrosis) and double-positive cells (indicative of advances stages of apoptosis).

Cells were harvested, washed twice with ice-cold PBS, resuspended in binding buffer at densities of around 1 x 10^6 cells per ml. 5 µl of Annexin-FITC-staining solution and 5µl of PI-staining solution were added to 100µl of each cell solution and stained at room temperature for 15 minutes in the dark before measurement. Cells treated with 20mM potassium cyanide (KCN) for 40 minutes were included as positive and untreated cells as negative controls. For optimal fluorophore compensation control cells were stained with FITC-only, PI-only and combinational staining. For selected experiments pan-caspase inhibitor zVAD was used at a concentration of 25µM.

Determination of mitochondrial morphology

Mitochondrial area, length, and width were determined under different stimulatory conditions via distance measurement using Fiji software. For each experiment, at least 5 different cells were measured in all dimensions for each cell line and condition. SPSS was used for statistical analysis applying repeated measures analysis.

SDS-PAGE and western blotting:

Immunoblotting was performed as described previously [1]. In brief, protein quantification was carried out using Lowry protein assay. Protein was loaded in SDS-gels of percentage adjusted to protein size of interest. Blotting was performed with a semi-dry blotting system and remaining gels were stained with Commassie to guarantee complete / even transfer. Blots were typically blocked with milk in TBS-Tween or PBS-Tween for 1 hour, washed three times and inoculated over night with first antibodies before washing and treatment with horseradish-peroxidase-coupled secondary antibodies and another washing step the following day before detection.

Western blots were analyzed using a Fusion-SL Advance 4.2 MP system (PeqLab). Antibodies used include anti-β-Actin (Santa Cruz C4), anti-ADPGK (Sigma-Aldrich HPA045194), anti-Calreticulin (Abcam FMC 75), anti-Bim/BOD (Enzo Life Sciences ADI-AAP-330-E), anti-Caspase-8 (R&D Systems AF1650-SP), anti-Digoxigenin-AP Fab fragments (Roche), ER Stress Antibody Sampler Kit (New England Biolabs 9956s), IAP Family Antibody Sampler Kit (New England Biolabs 9770T), Biotynilated DSL (Vector Labaratories), chicken anti-mouse IgG H&L (Abcam ab6814), chicken anti-rabbit IgG H&L IgG (Abcam ab6829), chicken anti-rat IgG (Abcam ab6836), anti-IP3R (Abcam ab108517), anti-O-GlcNAC (Cell signaling 9875S), Procaspase Antibody Sampler Kit (New England Biolabs 12742s), Phospho-SAPK/JNK (Cell signaling 9255S), Streptavidin-HRP anti-biotin IgG (Vector Laboratories), Stress and Apoptosis Antibody Sampler (New England Biolabs 8357T).

Lowry protein assay

Protein quantification was determined in a modified form of the calorimetric assay, originally described by Lowry et al, using the Protein Assay Reagents A and B (Biorad). For quantification, a standard curve was generated using bovine serum albumin (BSA) at concentrations ranging from 0.0 to 2.0 mg/ml in 1% SDS. 25µl of reagent A (alkaline copper tartrate solution, allowing for complex binding of copper ions and peptide bonds of proteins) was added to samples and standards. After brief shaking, 200µl of reagent B was added, followed by 15 minutes of incubation on a plate shaker at room temperature. Measurement was performed in quadruples at 750nm wavelength on a standard spectrophotometer (Spectramax Plus Microplate reader, Molecular Devices, CA, USA).

Photometric lactate and pyruvate measurement

Lactate and pyruvate content was determined as previously described [5] using an Olympus AU400 system (Olympus, Tokyo, Japan). For lactate measurement cell or zebrafish homogenate was mixed with NADH and LDH. For pyruvate measurement cell or zebrafish homogenate was mixed with reaction buffer 1 containing NADH, 1.5M Tris Base and 0.2 % $HClO_4$ and then with reaction buffer 2 containing LDH.

Glucose uptake

Glucose uptake was measured cytometricaly with the "Glucose Uptake cell-based Assay Kit" (Cayman Europe) as uptake of fluorescently labeled deoxyglucose 2-NBDG (2-(N-(7-nitrobenz-2-oxa-1,3-diazol-4-yl)amino)-2-deoxyglucose) according to the manufacturer's protocol on a FACSVerse cytometer (Becton Dickinson).
One day after 1:2 dilution with fresh medium, cells were counted and seeded into 6 well plates in glucose-free RPMI (Thermo Fisher Scientific). After 3 hours of glucose-deprivation, 100µg/ml of 2-NBDG was added and cells were stimulated with PMA (10 ng/ml) or PMA + Iono (10 µM) for 1 hour. For measurement cells were washed and resuspended in Cell-Based Assay. Cells were gated towards viable cells in forward-/site-scatter distribution. Unstained cells and cells treated with 0.1 mM Apigenin in DMSO to inhibit Glucose uptake were included as negative controls.

Quantification of adenosine, nucleotides and nucleotide sugars

Metabolite analysis in cell and zebrafish lysates was performed according to previously published protocols by reversed-phase HPLC electrospray ionization tandem mass spectrometry [2] [3]. Briefly, metabolites were extracted from 30 zebrafish embryos snap-frozen or about 40 × 10^6 cells per condition with 0.9% NaCl. Cells were split into three parts (adenosine analysis, nucleotide analysis and protein quantification), pelleted and snap-frozen and stored at -80°C until analysis at the the Metabolomics Core Technology Platform at the University of Heidelberg.
AMP, ADP and ATP were derivatized with chloroacetaldehyde at 80 ^oC, followed by injecting into an Acquity H-class UPLC system for separation. The UPLC was carried out by an Acquity BEH C18 150 mm × 2.1 mm, 1.7 µm with mobile phase A (5.7 mM TBAS, 30.5 mM KH_2PO_4 pH 5.8) and B (2/3 acetonitrile in 1/3 buffer A) at a flow rate of 0.45 ml/min. The separated derivates were detected by fluorescence (Acquity FLR detector, Waters, excitation: 280 nm, emission: 410 nm, gain: 100) and quantified using ultrapure standards. The Adenosine nucleotides were also analysed by HPLC: Acquity BEH C18 column (150 mm × 2.1 mm, 1.7 µm) connected to an Acquity H-class UPLC system was used for seperation. Following separation, adenosines were detected by fluorescence using an Acquity FLR detector (Waters, excitation: λ = 280 nm, emission: λ = 410 nm, gain: 100) and quantified using ultrapure standards. The Empower3 software suite (Waters) was used to acquire and process all data.

Subcellular fractionation and coupled enzyme assays

Enzyme activities in lysates and subcellular fractions of Jurkat T lymphocytes were monitored according to previously published protocols and normalized to protein content, determined via Lowry assay [1].

Subcellular fractionation:

In brief, a 27 3 1/200 needle was used to disrupt cells in ice-cold ETC (electron transport chain) buffer (250mM sucrose, 50mM KCl, 5mM $MgCl_2$, 20mM Tris/HCl, pH 7.4) and the homogenates were centrifuged at 4°C at 600g for 10 min. For preparation of the ‘‘high g’’ mitochondrial (pellet) as well as cytosolic (supernatant) fraction the 600g supernatant was centrifuged at 4°C at 11,000 g, for 20 min. For preparation of the ‘‘mitochondria-enriched’’ fractions, the 600xg supernatant was centrifuged at 4°C for 10 min at 3,500 g (pellet). Subsequently, for preparation of the ‘‘ER-enriched fraction’’ the 3,500xg supernatant was centrifuged at 4°C for 20 min at 11,000 g.

Enzyme measurements:

In brief, appropriate lysates (cytosolic fractions for glycolytic enzymes, mitochondrial fractions for respiratory chain measurements and GPD2, endoplasmic reticulum fractions for G6PDH, ADPGK) were measured in triplicates for each individual experiment in a 96well format in a spectrophotometer (Spectramax Plus Microplate reader, Molecular Devices, CA, USA). Mostly at 340/400nm. To allow subtraction of background signals, all measurements were acquired with and without addition of substrates. Temperature was 37°C if not otherwise stated. All enzymatic measurements were normalized to protein concentration, determined by a Lowry protein assay.

- ADP-dependent glucokinase (ADPGK) was measured as NADP reduction in ETC buffer containing 1 mM ADP, 1 mM glucose, 0.5 mM NADP, 5 mM Ap5A, and 0.05 U/ml glucose-6-phosphate dehydrogenase at pH 6.0 and 42°C.
- Hexokinase (HK) activity was assayed as NADP reduction in ETC buffer containing 1 mM ATP, 1 mM glucose, 0.5 mM NADP, 0.05 U/ml glucose 6-phosphate dehydrogenase.
- Phosphofructokinase (PFK) activity was detected as NADH oxidation in ETC buffer containing 4 mM fructose 6-phosphate, 2 mM ATP, 0.5 mM NADH, 0.2 U/ml aldolase, 0.8 U/ml TPI, 0.1 U/ml GPD1.

- Enolase (ENO) was detected as NADH oxidation in ETC buffer containing 1 mM 2-phosphoglycerate, 1 mM ADP, 0.5 mM NADH, 2 U/ml LDH and 2 U/ml PK.
- Pyruvate kinase (PK) was quantified as NADH oxidation in ETC buffer containing 1mM phosphoenolpyruvate, 1mM ADP, 0.5 mM NADH and 2 U/ml LDH.
- Lactate dehydrogenase (LDH) activity was recorded as NADH oxidation in ETC buffer containing 1mM pyruvate and 0.5mM NADH.
- ETC complex I / NADH dehydrogenase: 0.05 % laurylmaltoside, 200 µM NADH, 60 µM DBQ (ubiqinone) , measurement at 340/400nm
- ETC-complex III / Cytochrome c reductase: 100 µM oxidized Cytochrome c, 0.05% Triton X-100, 50 µM DBH (reduced ubiquinone), 2 mM KCN, measurement at 540/550nm
- ETC complex IV / Cytochrome C oxidase: 75 µM reduced cytochrome c, 250 mM sucrose, 120 mM KH_2PO_{4m} 0,05 % laurylmaltoside (at 25°C) , measurement at 540/550nm
- ETC: complex V / ATP-Synthase: 250 µM NADH, 1 mM PEP, 2,5 U/ml LDH, 2 U/ml pyruvate kinase, 2 mM ,1 µM DQA (2-decyl-4-quinazolinyl amine = complex I inhibitor), measurement at 340/400nm
- Electron flux from complex I to III: 0.05% digitonin-permeabilized cells were incubated with 150 µM oxidized cytochrome c, 200 µM NADH, 2 mM KCN, measurement at 540/550nm
- Electron flux from complex II to III: 0.05% digitonin-permeabilized cells were incubated with 150 µM oxidized cytochrome c, 30 µM succinate, 2 mM KCN, measurement at 540/550nm

Mitochondrial membrane potential / JC-1-assay

Mitochondrial membrane potential was measured cytometrically with “JC-1 Mitochondrial Membrane Potential Assay Kit”' (Cayman Europe) according to the manufacturer’s protocol. In brief, the fluorescent dye JC-1, which exhibits an emission shift from green (~529 nm) to red (590 nm) upon depolarization of mi-tochondria was used to stain cells and red/green-fluorescence-intensity-ratio was determined. One day after 1:2 dilution with fresh medium, cells were counted and plated into 6 well plates. After one hour of acclimatization time, cells were stimulated

with PMA (10 ng/ml) or PMA + Iono (10 µM) for 24 hour. After stimulation, 100µl of 1:10 diluted JC-1 staining solution were added per 1 ml of cell suspension and cells were incubated for staining in a cell culture incubator for another 30 minutes. Stained cell lysates were washed with assay buffer, transferred into tubes and directly subjected to cytometric analysis on a FACSVerse cytometer (Becton Dickinson).

Acridine orange staining of cells

Determination of acidic vesicles was performed by staining cells with Acridine orange as described previously [4]. Cells were harvested and washed in colorless RPMI 1640, incubated with 1µg/ml Acridine orange at 37°C for 20 minutes and washed with ice-cold colorless RPMI for several times to rinse away any acridine orange residues. Stained cells were analyzed using a FACSVerse cytometer (Becton Dickinson) utilizing the FITC channel for green and the PERCP-CY5.5 channel for red. Cells were gated for viability in FSC and SSC. The ratio of red mean fluorescence intensity, indicative of polymerized acridine orange in acidic vesicles, to green mean fluorescence intensity, indicative of unpolymerized dye, was determined for each sample. Increase in the ratio of MFI (Red/Green) was determined using the following formula: MFI-Increase (Red/Green)t = ((MFI(Red)t) / MFI (Green)t) - (MFI(Red)0h) / MFI(Green)0h)) for time point t.

Zebrafish husbandry

Wild-type AB (Danio rerio) zebrafish lines were maintained at 28.5°C on a 14-hour light/10-hour dark cycle. Embryos collected from natural mating were cultured in embryo E3 medium (150 mM NaCl, 0.5 mM KCl, 1.0 mM $CaCl_2$, 0.37 mM, KH_2PO_4, 0.05 mM Na_2HPO_4, 2.0 mM $MgSO_4$, 0.71 mM $NaHCO_3$ in deionized water, pH 7.4) and staged according to [42]. Zebrafish were held in accordance with all international and national laws and obligations as registered at the Regierungspräsidium Karlsruhe (Az. 35-9185.81/G-85/16).

Real time-quantitive PCR

Jurkat T Cell RNA and zebrafish RNA was prepared using “RNeasy kit” with on column-DNA-digestions (Qiagen) and Trizol® reagent with chloroform/isopropanol, respectively. The purified RNA was reverse-transcribed into cDNA using “Maxima First Strand cDNA Synthesis Kit” (Thermo Fisher Scientific). Quantitative real time

PCR was performed using "SensiFast SYBR Hi-ROX Kit" (Bioline) and a CFX Connect Real Time system (Bio-Rad). Human genes were normalized to 18S rRNA except for metabolic genes, which were normalized to GAPDH. Zebrafish genes were normalized to *ef1alpha*. A list of RT-qPCR primer is in suppl. table 5.

Whole mount in-situ hybridization (WISH)

DIG-labelled in situ hybridization antisense mRNA probes were synthesized and transcribed from a linearized PCRII dual promotor (ThermoFisher) vector. Sense probes were used as control. For respective primers see suppl. table 5. WISH was performed as shown by [5]. Briefly, Zebrafish embryos were collected at the desired stages and fixed with 4 % cold paraformaldehyde (PFA). After hydrating and dehydrating the embryos with a serial incubation of methanol and PBST, the embryos were digested with 10 ug/ml of Proteinase K in PBST and then hybridized with the *in situ* probe at 70 °C overnight. After washing and 4 hours of blocking with BSA, the embryos were incubated with anti-digoxigenin-AP, Fab fragments in blocking solution overnight at 4°C. Afterwards, the embryos were stained with NBT/BCIP in AP Buffer, washed with PBS and stored in 70% glycerol until observation.

Morpholinos and mRNA synthesis

Morpholinos (MO) were designed and provided by GeneTools LCC (Oregon, USA). Adpgk splice blocking MO1 targets the junction of exon 2 and intron 2: GTGTTCTCCAAGTTGCTCACCCCAC. Adpgk translation blocking MO2 targets the start codon: CAGCACAGCCTTCCTCCACATGA. As morpholino control (CO) a 5bp mismatch version of MO1 was used: GTATTATCCAAATTGCTAACCCAAC. The following MO concentrations were used, 300 µM for MO1, 250 µM for MO2 and 300 µM for CO. p53 MO (GCGCCATTGCTTTGCAAGAATTG) was injected at a concentration of 300 µM with or without 300 µM of MO1. The full length adpgk mRNAs used for rescue experiments were synthesized from zebrafish or human adpgk cDNA. Briefly, zebrafish or human adpgk cDNA was amplified, linearized and ligated into PCS2+ vectors. The ligates were then transformed into NEB 5-α competent E.coli. Subsequently, the purified and sequenced plasmids were used as the template and mRNAs were synthesized using MEGAscript ® T7 in vitro

Transcription Kit. Injections for adpgk overexpression experiments were carried out as with morpholinos.

TUNEL assay

Apoptosis in zebrafish embryos was detected via TUNEL assay (Roche, Basel, Switzerland). Embryos were fixed with 4 % PFA in PBS overnight. After fixation, embryos were washed twice with PBS for 5 minutes, dechorionated, and dehydrated in 50 % methanol in PBST for 5 minutes and 100 % methanol for 5 minutes. Afterwards, embryos were washed with acetone for 20 minutes and rehydrated by serial incubation in 75, 50, 25 % methanol and 100% PBST for 5 minutes. They were permeabilized with proteinase K for 5 minutes and fixed with 4% PFA for 20 minutes. The last step was repeated once. Embryos were washed 3 times with PBS-T for 20 minutes and stained with the TUNEL assay reagent for 1 hour at 37°C in the dark. Stained embryos were washed with PBS twice for 5 minutes and observed under a fluorescence microscope.

Acridine orange staining of zebrafish

Live imaging of Acridine orange staining was used to determine cell death. Embryos were stained with 5 µg/µL Acridine orange in E3 medium for 30 minutes followed by three times PBS wash. Stained embryos were observed under a fluorescence microscope.

Cell cycle analysis

Cell cycle analysis was performed with "Propidium Iodide Flow Cytometry Kit" (Abcam) according to the manufacturer's protocol using a FACSVerse cytometer (Becton Dickinson) with gating to viable cells in forward/site scatter. One day after 1:2 dilution with fresh medium, cells were counted and plated into 6 well plates. Following one hour of acclimazitation, cells were stimulated with/without PMA/Iono for various times before stimulation was terminated via washing with ice-cold PBS. Cells were then fixed and permeabilized with 66.7% ethanol in PBS and stored at 4°C until analysis. Staining was carried out with 200µl of staining solution for 30 minutes in a cell culture incubator at 37°C alongside RNAse-digestion to ensure that only DNA would be stained by periodide. Distribution between 2N (G1-phase), 2-4N (S-phase), 4N (G2-phase), < 2N and > 4N was calculated in %. Quantification of cell

cycle distribution was calculated using the Dean-Jett-Fox model in FlowJo V10.

Whole mount zebrafish glucose measurement

Zebrafish glucose content was measured by a "glucose colorimetric assay kit" (Cayman Europe) according to the manufacturer's protocol and normalized to protein content of fish. In short, 15 µL of standard solution (0, 2.5, 5, 7.5, 10, 15, 20, 25 mg/dl) or zebrafish homogenate was mixed with 85 µl of diluted assay buffer in 96 well plates. The reaction was initiated by adding 100 µl of enzyme mixture and after 10 minutes of incubation at 37 ^{o}C the absorbance was measured on a standard spectrophotometer (Spectramax Plus Microplate reader, Molecular Devices, CA, USA).

Glycan analysis

Cell lysis

Cells pellets were mixed with 8 M urea in Tris-HCl pH 8.5 (UA solution, 100 µL per $1x10^6$ cells) and disrupted by three sonication cycles (each 15 seconds). Lysed cells were centrifuged at 16,060 *g* for 20 minutes, and clarified supernatant was collected. Bradford assay was performed to determine protein concentration.

N-glycan release

8.5 mg of proteins were transferred onto a 10 kDa MWCO spin filter (Amicon Ultra-0.5 mL). DTT was added (of 10 mM DTT in UA) and incubated at RT for 30 min. DTT excess was removed by washing the filter with fresh UA solution. Alkylation was performed by adding 10% (v/v) iodoacetamide (50 mM in UA). Samples were incubated at RT for 30 min in darkness. Afterwards, samples were centrifuged for exchanging buffer to 50 mM ammonium bicarbonate (pH ~7.8). 5 µL of PNGaseF (NEB) were added to the filter and incubated in a thermomixer at 37˚C, 400 rpm, overnight. Released N-glycan were collected by spinning the filter device at max speed, 4°C for 10 min. Retained proteins were washed twice with 200 µl of milliQ water. Eluates were collected and lyophilized.

N-Glycan Labelling, Clean-up, and Enzymatic Processing

Released N-glycan were reconstituted in 50 μL of 1% formic acid, thus converted to reducing aldoses, lyophilized and derivatized for two hours at 65°C with 10 μL 2-aminobenzamide (2-AB) via reductive amination with sodium cyanoborohydride in 30% v/v acetic acid in DMSO. Removal of excess labelling agent was conducted by frontal HILIC purification using a Thermo UltiMate3000 RS UHPLC system (Thermo Fisher Scientific), monitored by fluorescence detection, $\lambda_{ex/em}$ = 330/420. Samples were loaded onto a BEH Glycan Amide, 1.7 μm, 2.1 x 50 mm column (Waters, Milford, MA) at 0.5 mL/min for 2.5 minutes in 85% acetonitrile, 15% 50 mM ammonium formate pH 4.5 v/v. Purified samples were eluted for 2.5 min in 20% aqueous acetonitrile and reduced to dryness.

For structural characterization, a set of recombinant exoglycosidases were used in 50 mM ammonium acetate buffer, pH 5.5 in a final volume of 10 μL at 37°C overnight. Amounts of enzyme used per digestion were 1 μL of neuraminidase (P0720, New England Biolabs), 2 μL of β1-4 galactosidase (P0730), 2 μL of β1-3,4 galactosidase (P0746), 1 μL of α1-3,4 Fucosidase (P0769) , 1 μL of α-Fucosidase (GKX-5006, Prozyme, Hayward, CA), 2 μL of α-galactosidase (GKX-5007), 2 μL of β-N-Acetylhexosaminidase (GK80050). The enzymes were removed using HILIC chromatography after digestion, dried *via* vacuum centrifugation and re-suspended in water for analysis by hydrophilic interaction UPLC-FLR-MSE.

Weak anion exchange separation of labeled N-glycans

Fractionation of N-glycan based upon their degree of sialylation was conducted by anion exchange chromatography using a Waters BioSuite DEAE 10 μm AXC, 7.5 x 75 mm column. Glycans were eluted by a 35 minute linear gradient of 100 mM acetate, pH 7.0 in 20% v/v acetonitrile at 0.75 mL/min.

Analysis of 2-AB labeled glycan by LC-MS

Hydrophilic interaction UPLC-FLR-MS analysis was conducted on a Waters Acquity™ UPLC system with online fluorescence detection hyphenated to a Waters Xevo G2 QToF mass spectrometer through an electrospray ionization interface. A linear gradient of 74-55 % acetonitrile in 38 minutes was applied at 0.15 mL/min and at 60 °C using a glycan BEH Amide column, 1.7 µm, 1.0 x 150 mm. 8 µL sample was injected in 75 % v/v acetonitrile. Negative ionisation mode with a capillary voltage of 1.80 kV was applied. Ion source and nitrogen desolvation gas (600 L/h flow rate) temperatures were set to 120 °C and 400 °C, respectively and the cone voltage was kept at 50 V. Full-scan MS data scan range was set to 450-2500 m/z. Data collection and processing was carried out using MassLynx 4.1. Glycan structures are presented using CFG symbol nomenclature [6]. Label free comparison of N-glycan structures was performed with ProgenesisQI software (Waters).

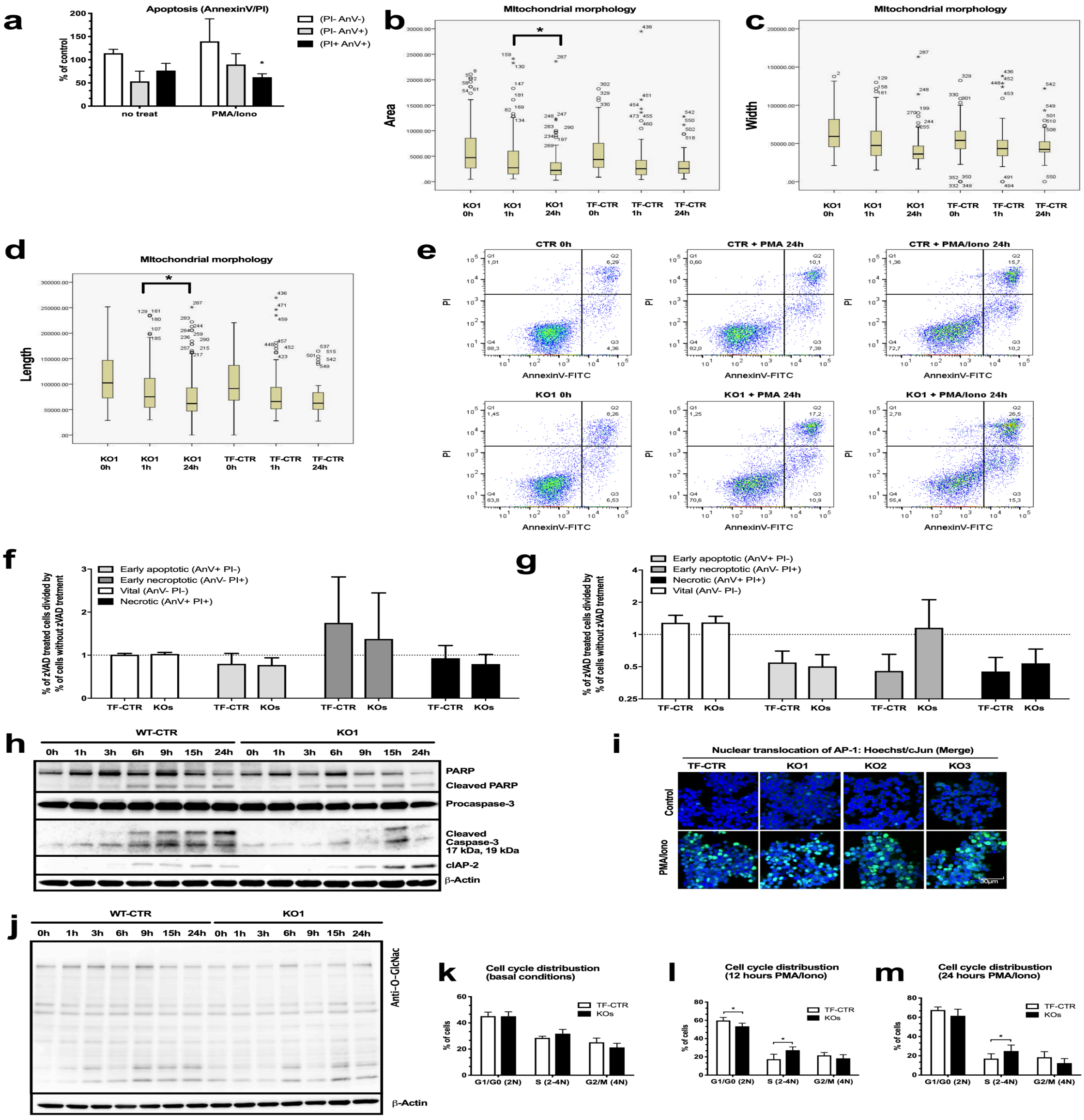

Supplementary figure 1. AICD-like cell death in ADPGK KO cells

(a) Cytometric cell death analysis via propidium iodide and annexin V staining under basal and stimulatory conditions (PMA/Iono for 24 hours) in ADPGK overexpressing cells. N=3 independent experiments. **(b – d)** Area, length and width of mitochondria after 1h and 24h of PMA/Iono stimulation, measured by Fiji software and analyzed using ANOVA with contrasts (SPSS statistics). **(e)** Representative contour plots of cytometric cell death analysis depicted in figure 2a – c. **(f – g)** Cell death analysis via propidium iodide and annexin V staining upon pre-incubation of Jurkat T cells with pan-Caspase inhibitor zVAD (20µM) **(f)** without subsequent stimulation and with **(g)** PMA/Iono stimulation for 24h. N=3 independent experiments. **(h)** Immunoblot kinetic analysis of PARP-cleavage, Caspase 3-cleavage and cIAP2 expression under basal

conditions and under stimulation with PMA/Iono. **(i)** Nuclear translocation of AP-1 detected via confocal microscopy using c-Jun-specific antibody and Hoechst counterstaining. **(j)** O-GlcNAc lysates of control and ADPGK KO cells co-stimulated with PMA/Iono for different time periods. N=3 independent experiments. **(k – m)** Cell cycle analysis via cytometric quantification of cellular DNA content after PI-staining of control and ADPGK KO cells **(j)** without stimulation as well as after **(k)** 12h and **(l)** 24h PMA/Iono stimulation. N=3 independent experiments. Mean of KO1, KO2, and KO3 are shown. * $p<0.05$.

All images of blots represent cropped blots of appropriate protein size. For full length blots see supplemental figure 3.

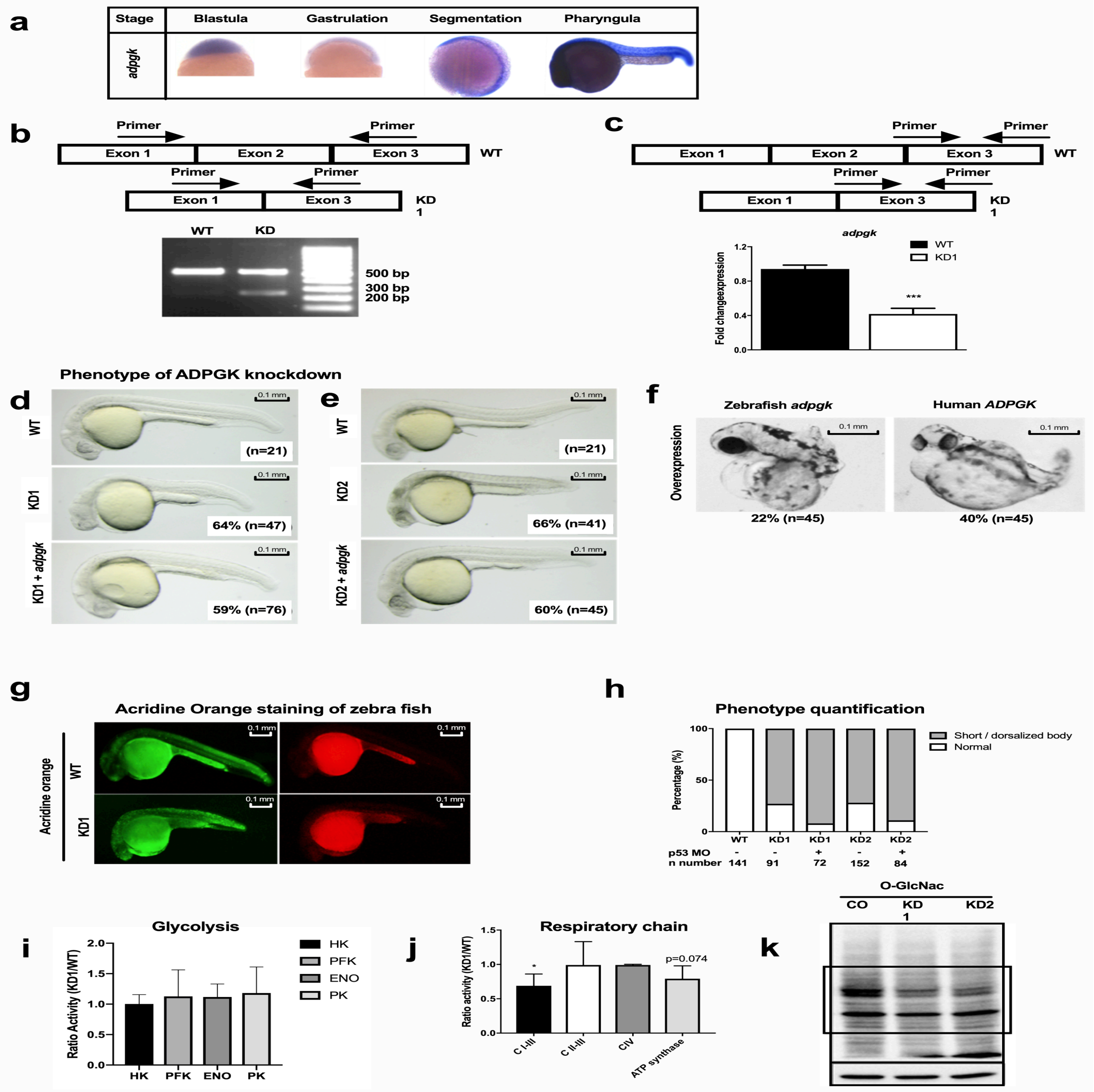

Supplementary Figure 2. Phenotype of Adpgk hypomorphic embryos

(a) WISH staining of *adpgk* in wild type embryos during different developmental stages. **(b)** Expression of a truncated *adpgk* transcript in KD1 embryos (stage 8 hpf). **(c)** RT-qPCR analyses of *adpgk* expression in wild type and KD1 embryos. N=6 independent experiments, stage 8 hpf. **(d, e)** Representative images of **(d)** KD1 and **(e)** KD2 embryos (stage 24 hpf; lateral views with anterior to the left) rescued with zebrafish Adpgk mRNA as well as total number of tested embryos and percentage of rescued embryos. **(f)** Representative images of zebrafish embryos after overexpression of zebrafish and human ADPGK mRNA (stage 72 hpf; lateral views

with anterior to the left). **(g)** Acridine Orange staining of wild type and KD1 embryos (stage 60hpf; lateral views with anterior to the left). **(h)** Quantification of the phenotypes of KD1 and KD2 embryos with and w/o additional blocked *p53* expression. **(i)** Changes of enzymatic activities of Hexokinase (HK), Phosphofructokinase (PFK), Enolase (ENO) and Pyruvate kinase (PK) in in wild type and KD1 embryos. N=4 independent experiments, stage 22 hpf. **(j)** Kinetic changes of electron transport from Complex I-III and Complex II-III as well as activity of Complex IV and ATP synthase in wild type and KD1 embryos. N=4 independent experiments, stage 22 hpf. **(k)** Representative immunoblots for GlcNAcylated residues in control, KD1 and KD2 embryos using a monoclonal antibody against O-GlcNAc. * $p<0.05$.

All images of blots represent cropped blots of appropriate protein size. For full length blots see supplemental figure 3.

Supplemental Table 1

Mutations of ADPGK KO cell lines

KO nr.	Status	Mutation
KO1	Compound heterozygous	(c.250_251insG)<br>(c.240_249del, c.250_251insCC, c.254_266del, c.269_270delinsCC)
KO2	Compound heterozygous	(c.250_251insGC)<br>(c.250_251insGCAA)
KO3	homozygous	(595_596insCCC)
KO4	homozygous	(c.250_251insCCC)

Supplemental Table 2

Metabolic characterization of control cells and ADPGK KO clones without treatment. Mean of N=4 independent experiments of CTRs (TF-CTR and WT-CTR) and KOs (KO1, KO2, and KO3) are shown. * $p<0.05$.

(nM/mg protein)	CTRs		KOs	
	**Average**	**+/- SD**	**Average**	**+/- SD**
Uridin	0.12	0.10	0.11	0.05
Guanosin	0.05	0.04	0.03	0.01
Adenosin	0.15	0.03	0.13	0.02
UDP-Gal	1.13	0.28	1.43	0.36
UDP-Glc	2.31	0.71	2.45	0.81
GDP-Man	1.15	0.22	1.20	0.14
UDP-GlcNAc	0.07	0.05	0.03*	0.03
CDP	0.84	0.48	0.69	0.26
AMP	0.62	0.34	0.50	0.15
UDP	2.25	1.20	1.77	0.67
GDP	6.22	1.83	6.34	0.80
ADP	7.03	3.09	6.48	1.87
CTP	8.57	1.69	8.87	2.44
GTP	28.01	4.25	31.03	3.07
UTP	39.13	6.65	36.51	7.63
ATP	102.87	5.75	110.14	14.57
SHC	0.41	0.09	0.39	0.09
MTA	0.49	0.16	0.48	0.15
AMP	1.05	0.62	0.66*	0.17
ADP	9.66	2.89	8.24	2.11
ATP	105.95	22.72	101.99	22.56
NAD	0.96	0.39	0.76	0.32
NADH	2.69	0.54	2.40	0.72
NADPH	1.55	0.36	1.24*	0.29
SuccinylCoA	1.42	0.57	1.41	0.49
AcetylCoA	0.92	0.58	0.93	0.47
MalonylCoA	0.65	0.44	1.00*	0.33

Supplemental Table 3

2-AB derivatized N-glycan analyses of control and KO1 cells with and without 24 PMA/Iono activation analyzed *via* HILIC-FLR-MS. Quantitative comparison of the samples was performed through label-free data analysis. All structures presented showed changes with Anova p-value < 0.05.

**Retention time (min)**	**Charge**	**Experimental mass m/z**	**Mass accuracy (ppm)**	**Identifications**	**Max Fold Change**	**Highest Mean**	**Lowest Mean**
22.27	2	1170.412	5.6	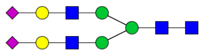	55.4	KO_stimulated with PMA	KO_unstimulated
21.93	2	1170.919	8.4	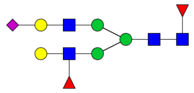	33.3	KO_stimulated with PMA	CTRL_unstimulated
11.66	2	871.323	3.1	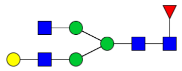	25.3	CTRL_unstimulated	KO_unstimulated
20.38	2	1199.433	5.4	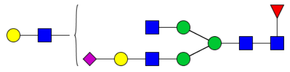	19.2	CTRL_unstimulated	KO_stimulated with PMA

23.79	2	1317.471	10.1	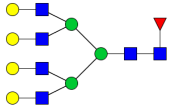	18.6	CTRL_stimulated with PMA	KO_unstimulated
15.89	2	1053.885	6.5	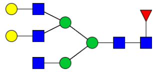	17.0	CTRL_unstimulated	KO_stimulated with PMA
23.49	2	1243.435	10.1	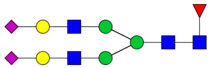	15.8	KO_stimulated with PMA	KO_unstimulated
20.85	2	1077.380	-13.8/6.1	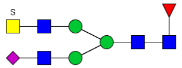 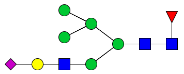	14.9	KO_stimulated with PMA	KO_unstimulated
18.04	2	996.3542	6.0	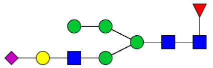	10.7	KO_stimulated with PMA	KO_unstimulated
15.24	2	952.343	9.3	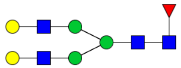	10.24941	KO_stimulated with PMA	KO_unstimulated

13.18	2	993.374	5.2	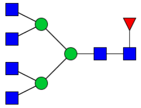	9.890761	KO_stimulated with PMA	KO_unstimulated
26.54	2	1426.003	7.5	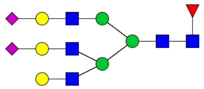	9.3	CTRL_stimulated with PMA	KO_unstimulated
15.09	2	858.8024	16.2/8.7	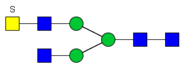 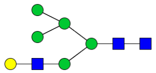	9.3	KO_stimulated with PMA	KO_unstimulated
12.86	2	842.809	7.3	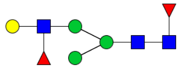	8.2	KO_stimulated with PMA	KO_unstimulated
18.13	2	1024.862	8.7	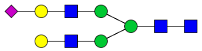	7.5	KO_stimulated with PMA	KO_unstimulated
10.82	2	891.834	6.0	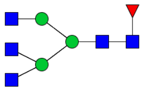	7.5	KO_stimulated with PMA	KO_unstimulated

15.41	2	915.328	6.2	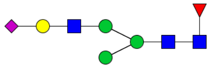	7.2	KO_stimulated with PMA	KO_unstimulated
26.71	2	1463.018	9.6	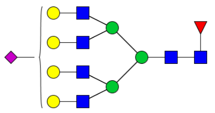	6.8	CTRL_stimulated with PMA	KO_unstimulated
9.27	2	790.294	7.0	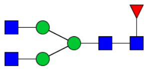	6.5	KO_stimulated with PMA	KO_unstimulated
11.61	2	749.271	2.9	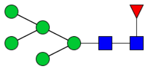	6.2	KO_stimulated with PMA	KO_unstimulated
8.34	1	1337.496	3.0	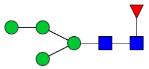	6.1	KO_stimulated with PMA	KO_unstimulated
22.13	2	1243.436	9.3	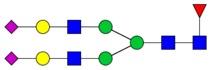	5.8	KO_stimulated with PMA	KO_unstimulated

14.86	2	830.293	7.4	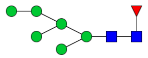	5.4	KO_stimulated with PMA	KO_unstimulated
11.70	2	891.8326	7.2	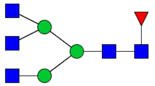	5.3	KO_stimulated with PMA	KO_unstimulated
10.08	1	1353.494	0.7	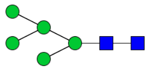	5.2	KO_stimulated with PMA	KO_unstimulated
3.61	1	1013.392	2.3	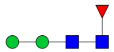	5.2	KO_stimulated with PMA	KO_unstimulated
4.71	1	1029.385	4.1	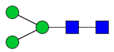	5.1	KO_stimulated with PMA	KO_unstimulated
24.2	2	1081.363	11.9	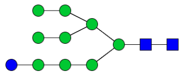	4.9	CTR_unstimulated	KO_unstimulated

16.53	2	838.2935	3.7	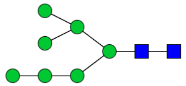	4.8	KO_stimulated with PMA	KO_unstimulated
2.82	1	867.334	2.8	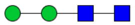	4.8	KO_stimulated with PMA	KO_unstimulated
6.90	1	1191.438	3.4	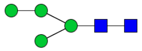	4.7	KO_stimulated with PMA	KO_unstimulated
25.32	2	1426.000	9.6	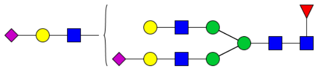	4.7	KO_stimulated with PMA	KO_unstimulated
19.57	2	919.3217	1.5	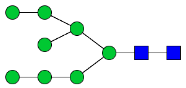	4.6	KO_stimulated with PMA	KO_unstimulated
23.09	2	1280.454	9.3	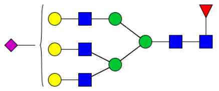	4.5	KO_stimulated with PMA	KO_unstimulated

5.85	1	1175.445	1.8	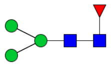	4.4	KO_stimulated with PMA	KO_unstimulated
27.49	3	1047.701	10.4	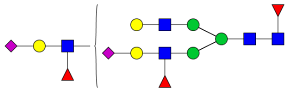	4.3	KO_stimulated with PMA	KO_unstimulated
7.55	1	1378.523	2.6	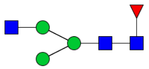	4.2	KO_stimulated with PMA	KO_unstimulated
21.91	2	1000.344	5.5	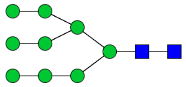	3.9	CTR_unstimulated	KO_unstimulated
16.94	2	898.7848	9.2	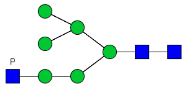	3.6	KO_stimulated with PMA	KO_unstimulated
13.19	2	757.2662	5.3	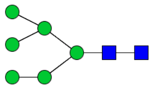	3.5	KO_stimulated with PMA	KO_unstimulated

Supplemental table 4

2-AB derivatized N-glycan analyses of wild type and KD1 embryos (stage 22 hpf) analyzed *via* HILIC-FLR-MS. Quantitative comparison of the samples was performed through label-free data analysis. Anova p-values are included in the table.

Retention time (min)	Charge	Experimental mass m/z	Mass accuracy (ppm)	Identifications	Anova (p)	Max Fold Change	Highest Mean	Lowest Mean
30.99	2	1494.524	0.2	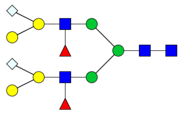	≤ 0.05	5.8	wild type	KO cells
15.09	2	858.802	9.2	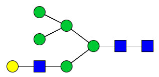	≥0.05	1.6	wild type I	KO cells
3.94	1	1070.413	2.6	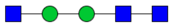	≥0.05	1.6	KD1	Control
20.98	2	1105.892	4.8	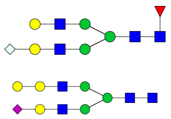	≥0.05	1.5	wild type	KO cells

19.57	2	1032.859	9.0	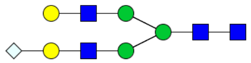	≤ 0.05	1.3	wild type	KO cells
29.50	2	1486.514	8.6	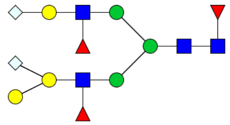	≤ 0.05	1.2	wild type	KO cells
19.55	1	1839.645	7.1	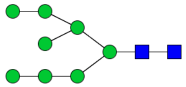	≤ 0.05	1.2	wild type	KO cells
21.93	2	1000.348	1.1	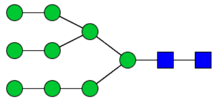	≤ 0.05	1.2	wild type	KO cells
16.55	2	838.293	4.3	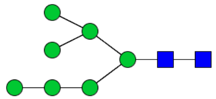	≤ 0.05	1.1	wild type	KO cells
25.81	2	1332.969	9.5	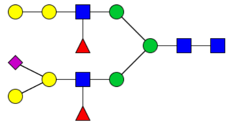	≤ 0.05	1.1	wild type	KO cells

4.713	1	1029.386	3.1	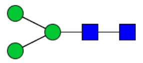	≤ 0.05	1.1	wild type	KO cells
10.09	1	1353.491	2.9	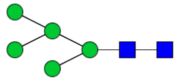	≤ 0.05	1.1	wild type	KO cells
13.23	2	757.266	5.7	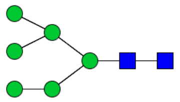	≤ 0.05	1.1	wild type	KO cells
24.20	2	1081.368	7.3	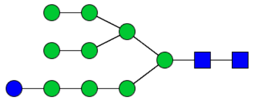	≥0.05	1.1	wild type	KO cells
18.68	2	1097.889	9.9	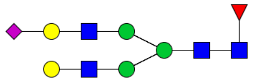	≥0.05	1.1	KD1	Control
27.44	2	1340.975	3.0	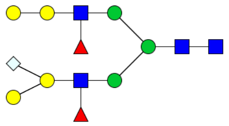	≥0.05	1.1	KD1	Control

19.09	2	1097.892	7.1	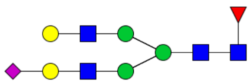	≥0.05	1.0	wild type	KO cells
7.90	2	717.267	4.7	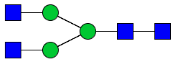	≥0.05	1.0	KD1	Control
11.87	2	777.781	3.2	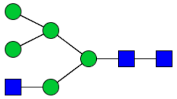	≥0.05	1.0	wild type	KO cells

Supplemental Table 5

RT-qPCR primer

Jurkat T		
18S rRNA	FW	GTAACCCGTTGAACCCCATT
	RW	CCATCCAATCGGTAGTAGCG
CDKN1A	FW	CCTCATCCCGTGTTCTCCTTT
	RE	GTACCACCCAGCGGACAAGT
GAPDH	FW	AGGGCTGCTTTTAACTCTGGT
	RE	CCCCACTTGATTTTGGAGGGA
XBP1 spliced	FW	GGTCTGCTGAGTCCGCAGCAGG
	RE	GGGCTTGGTATATATGTGG
zebrafish		
adpgk	FW	CGCTGGGGATGGGTTATCTC
	RE	GTAGCACCTCGTGGTCTCTG
bbc3	FW	ACATCCCCTCACATGATGCC
	RE	TCTGTTCCTGAATTGTCCCTGAG
bcl	FW	GGAAAATGGAGGTTGGGATGC
	RE	AAACGGGTGGAACACAGAGT
bcl2l	FW	GCAGATTGTGTTATGGGTATGAGC
	RE	TGTTGCTCGTTCTCCGATGT
cdkn1a	FW	GCAGAAGCTCAAAACATATTGTCAC
	RE	CGATGCGTCCTCCAGATCG
ef1a	FW	ACCGGCCATCTGATCTACAA
	RE	CAATGGTGATACCACGCTCA
gadd45aa	FW	GTCCCTCAATGTGGACCCTG
	RE	GTTGATGTCGTTCTCGCAGC
hk1	FW	GGTGAATTGGACGAAGGGCTTTAA
	RE	CCTCTTGATCCCCTCTCTCAGAAG
ins	FW	CTCTGTTGGTCCTGTTGGTC
	RE	CTCAAAGATGCTGCAGGGT

Primers for adpgk rescue mRNA synthesis

zebrafish adpgk rescue	FW	GAGAGGGATCCCTCAACTTCTCCACATACA
	RE	GAAGAGAATTCGGATGTTTCTTTTTATTTGAGTGA
human adpgk rescue	FW	GAGAGATCGATGTAGCGCTTGTGTCG
	RE	GAAGAGAATTCCCTGAAATGTAAATTGTTTTTAATATATTTAAGAG

References

1. Kamiński, M. M. *et al.* T cell Activation Is Driven by an ADP-Dependent Glucokinase Linking Enhanced Glycolysis with Mitochondrial Reactive Oxygen Species Generation. *Cell Rep.* **2**, 1300–1315 (2012).
2. Hartmann, S. *et al.* Comprehensive detection of disorders of purine and pyrimidine metabolism by HPLC with electrospray ionization tandem mass spectrometry. *Clin. Chem.* **52**, 1127–37 (2006).
3. Bürstenbinder, K., Rzewuski, G., Wirtz, M., Hell, R. & Sauter, M. The role of methionine recycling for ethylene synthesis in Arabidopsis. *Plant J.* **49**, 238–49 (2007).
4. Rundquist, I., Olsson, M. & Brunk, U. Cytofluorometric quantitation of acridine orange uptake by cultured cells. *Acta Pathol. Microbiol. Immunol. Scand. A.* **92**, 303–9 (1984).
5. Thisse, C. & Thisse, B. High-resolution in situ hybridization to whole-mount zebrafish embryos. *Nat. Protoc.* **3**, 59–69 (2008).
6. Varki, A. *et al.* Symbol Nomenclature for Graphical Representations of Glycans. *Glycobiology* **25**, 1323–4 (2015).

Supplemental figure 3: Full length blots and gels

In figures 1-5 and supplemental figure 1 immunoblots are depicted in cropped form, adjusted to the appropriate protein size. Here you find full length blots with appropriate size markers wherever possible.

ADP-dependent glucokinase regulates energy metabolism via ER-localized glucose sensing

Imle Roland[1]*, Wang Bei-Tzu[1]*, Stützenberger Nicolas[1]*, Birkenhagen Jana[1], Tandon Amol[1], Carl Matthias[11,12], Himmelreich Nastassja[1], Thiel Christian[1], Gröne Hermann-Josef[2], Poschet Gernot[3], Völkers Mirko[4,5], Gülow Karsten[6,7], Schröder Anne[6], Carillo Sara[8], Mittermayr Stefan[8], Bones Jonathan[8,9], Kaminski Marcin Mikolaj[10], Kölker Stefan[1], Sauer Sven Wolfgang[1 #]

*contributed equally

#corresponding author, Sven77sauer@icloud.com, +4915226764047

1 Division of Child Neurology and Metabolic Diseases, Centre for Child and Adolescent Medicine, Im Neuenheimer Feld 430, D-69120, Heidelberg, Germany.

2 Department of Cellular and Molecular Pathology, German Cancer Research Center, Heidelberg, Germany.

3 Centre for Organismal Studies (COS), Heidelberg University, Heidelberg, Germany.

4 German Centre for Cardiovascular Research (DZHK), partner site Heidelberg/Mannheim, Heidelberg, Germany.

5 Department of Cardiology, Heidelberg University, Heidelberg, Germany.

6 German Cancer Research Center, 69120 Heidelberg, Germany.

7 University Hospital Regensburg, Internal Medicine I, Regensburg, Germany

8 Characterisation and Comparability Laboratory, The National Institute for Bioprocessing Research and Training (NIBRT), Foster Avenue, Mount Merrion, Blackrock, Co. Dublin, A94 X099, Ireland.

9 School of Chemical and Bioprocess Engineering, University College Dublin, Belfield, Dublin 4, D04 V1W8, Ireland.

10 Department of Immunology, St. Jude Children's Research Hospital, 262 Danny Thomas Place, Memphis, Tennessee 38105, USA.

11 Heidelberg University, Medical Faculty Mannheim, Department of Cell and Molecular Biology, 68167 Mannheim, Germany

12 University of Trento, Center for Integrative Biology (CIBIO), Laboratory for Translational Neurogenetics, 38123 Trento, Italy

Full images of western blots of ER-fractions stained for ADPGK(Fig. 1a)

Blot 1 (developed for short period) Blot 2 (processed and developed in parallel, 60minutes)

Acquired with whole protein antibody that typically results in two bands, Sigma HPA045194, 1:250)

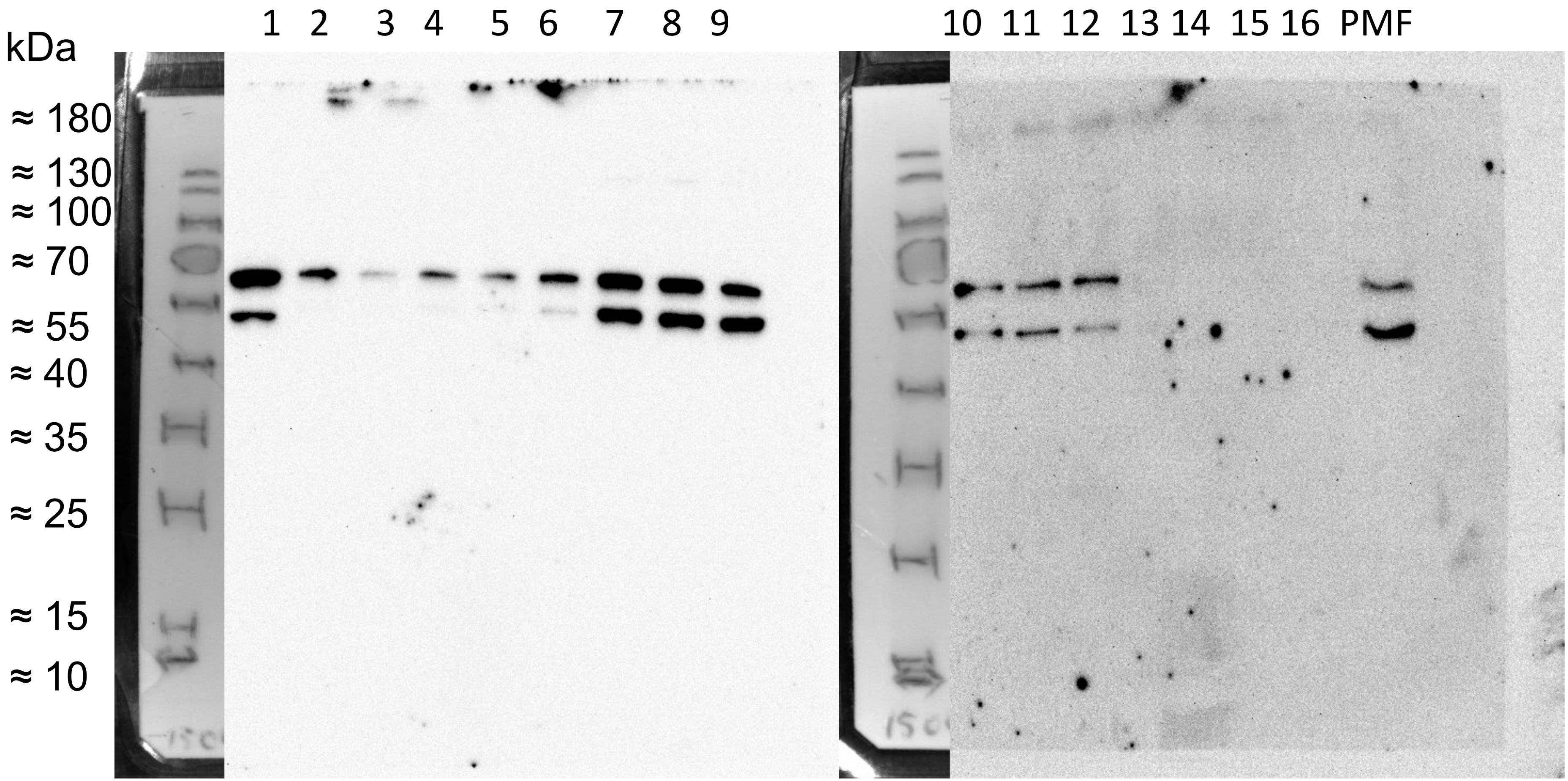

Full images of western blots of ER-fractions stained for Calreticulin (Fig. 1a)

Blot 1 Blot 2 (processed and developed in parallel)

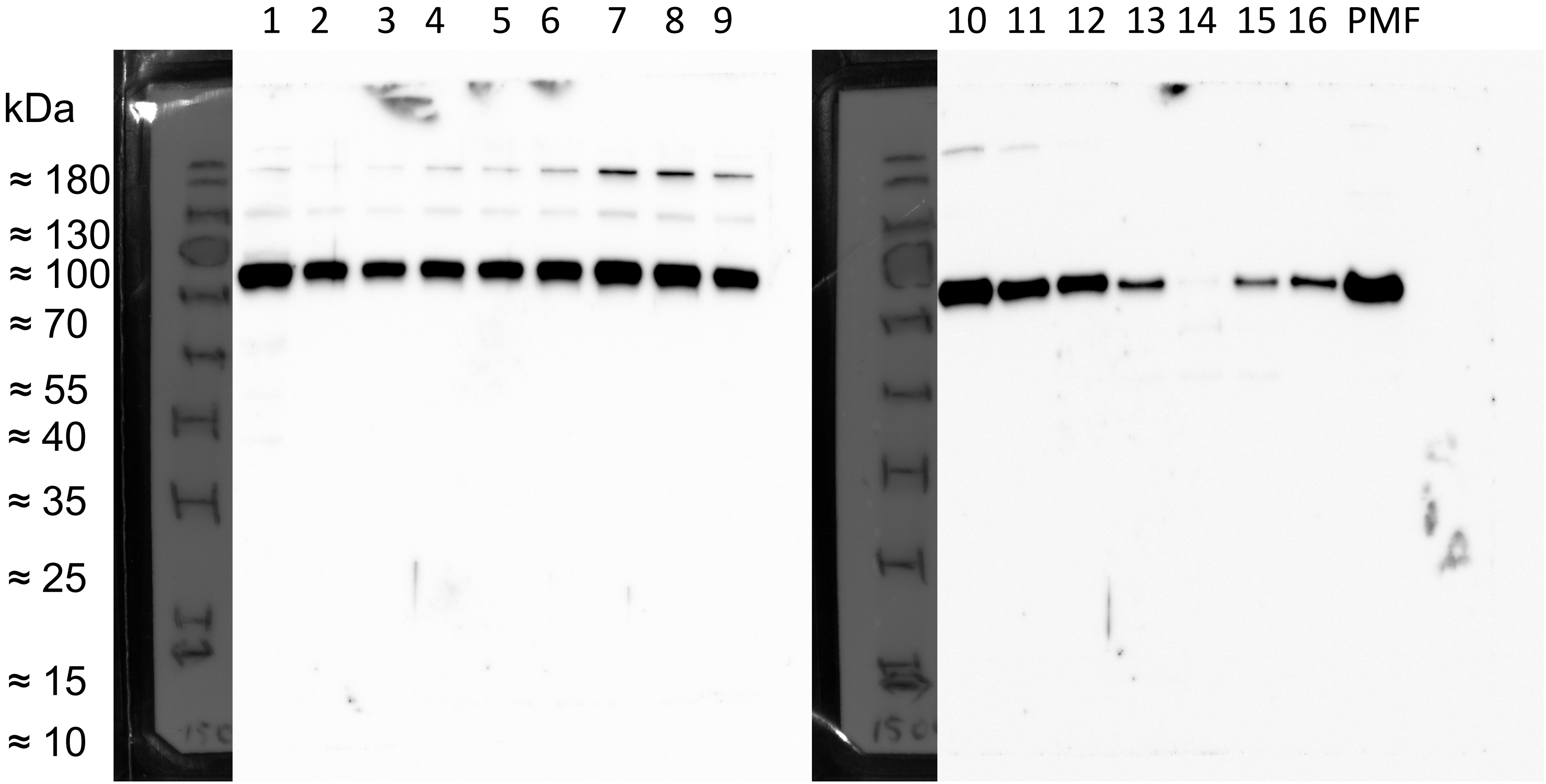

Full images of western blots of ER-fractions stained for IP3R-1(Fig. 1a)

Blot 1 Blot 2 (processed and developed in parallel)

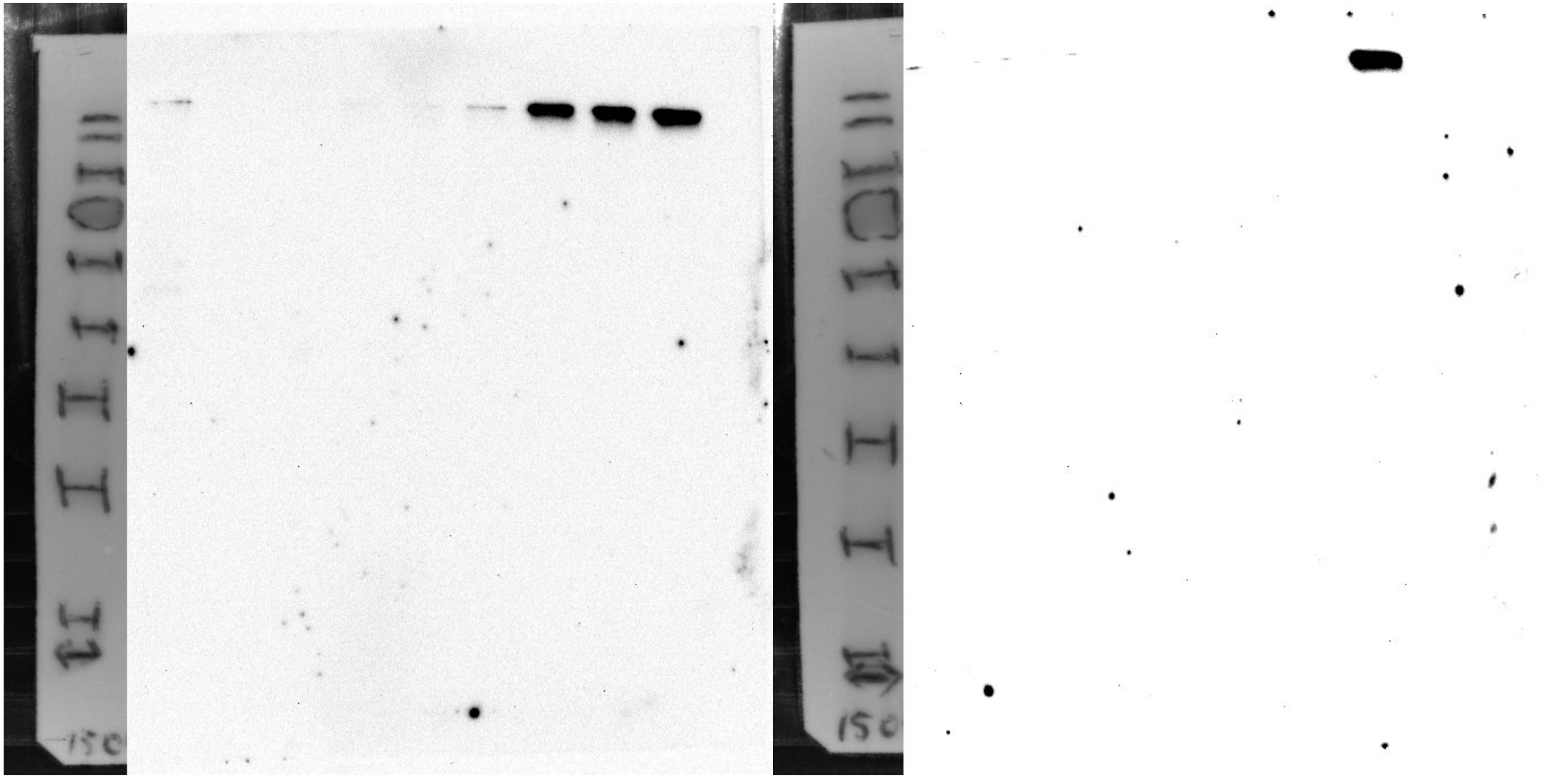

Full images of western blots of ER-fractions stained for SRPRbeta (Fig. 1a)
Size appropriate, depiction of size marker missing

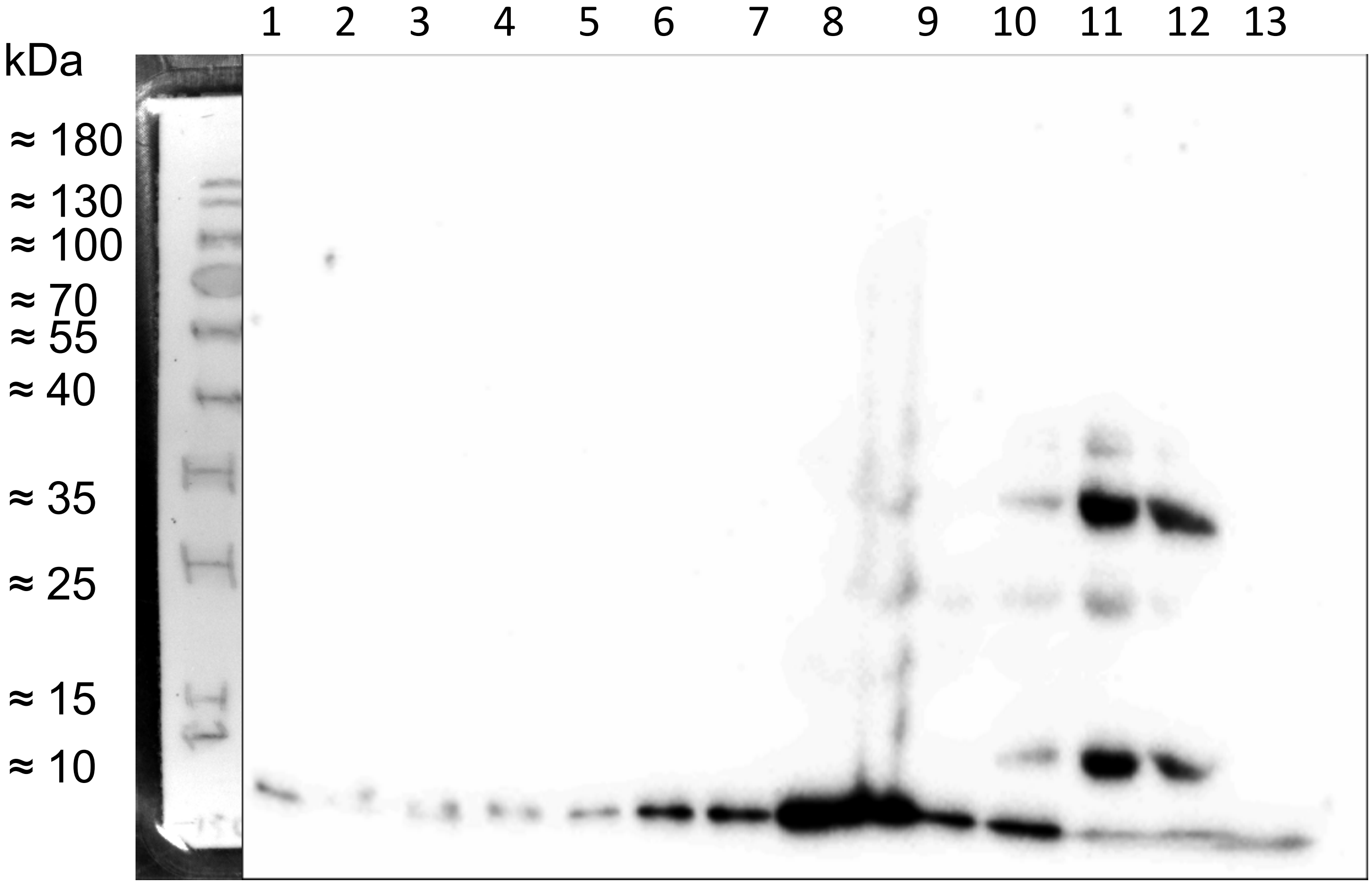

Full images of western blots of ER-fractions stained for SRPRbeta (Fig. 1a)
Size appropriate, depiction of size marker missing

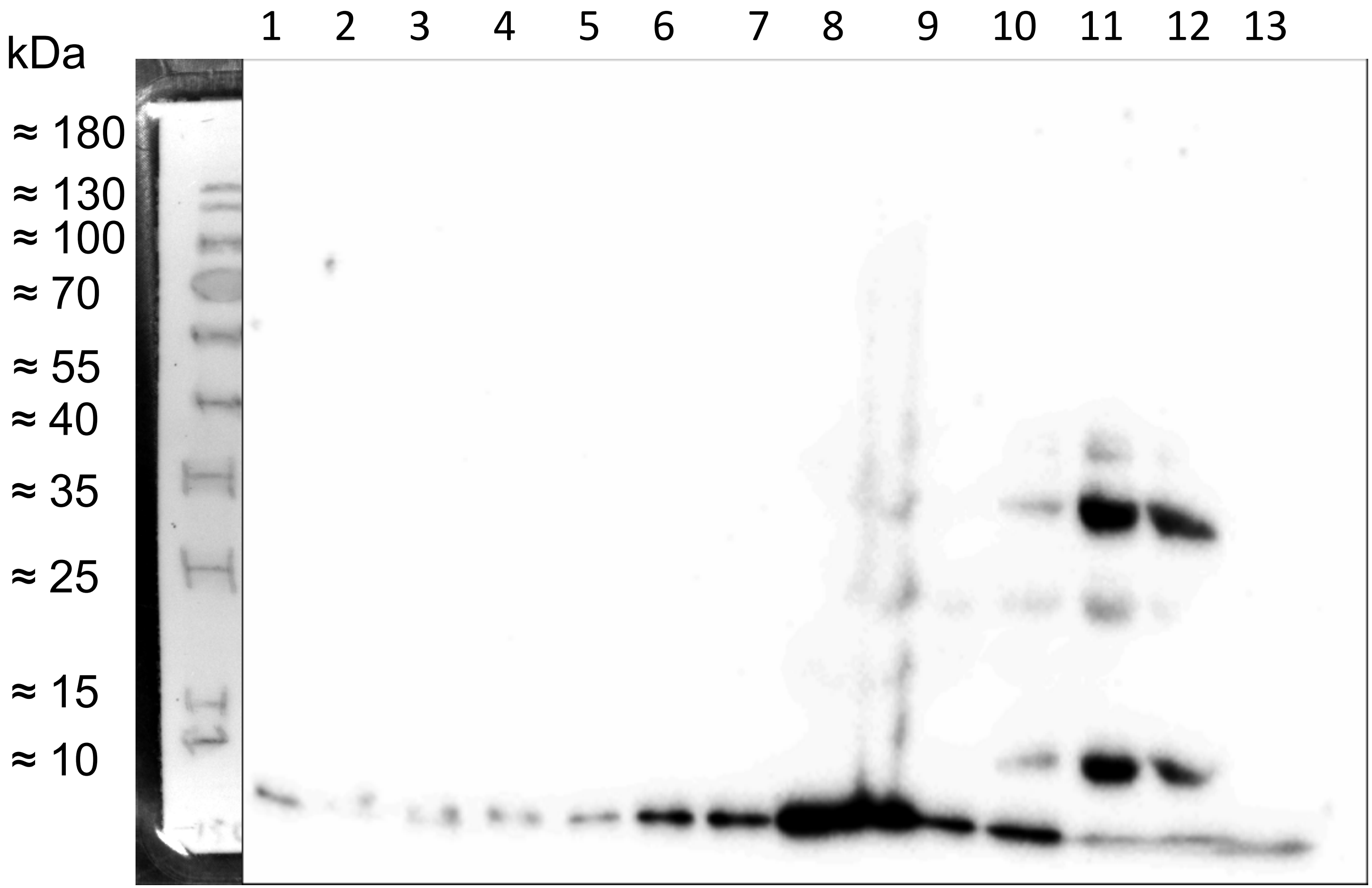

Full images of western blots of ER-fractions stained for ADPGK(Fig. 1a)
Size appropriate, depiction of size marker missing (maker from comparable blotting membrane depicted), additional bands caused by simultaneous co-staining for GFAT (Glutamine-fructose-6-phosphate transaminase 1) (78,8 kDa)

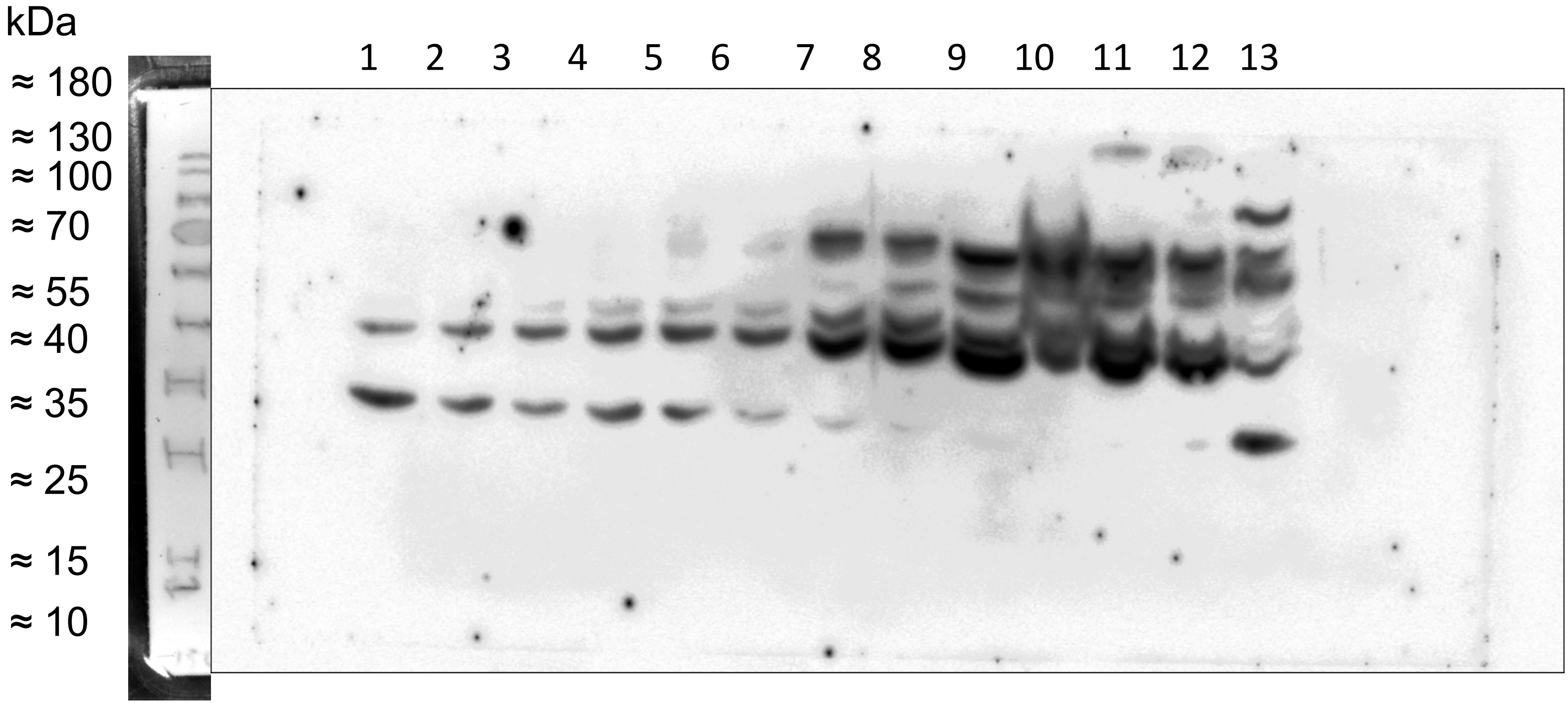

Full images of Western blots for ADPGK (Fig. 1c) on the left and β-actin (Fig. 1c) on the right.

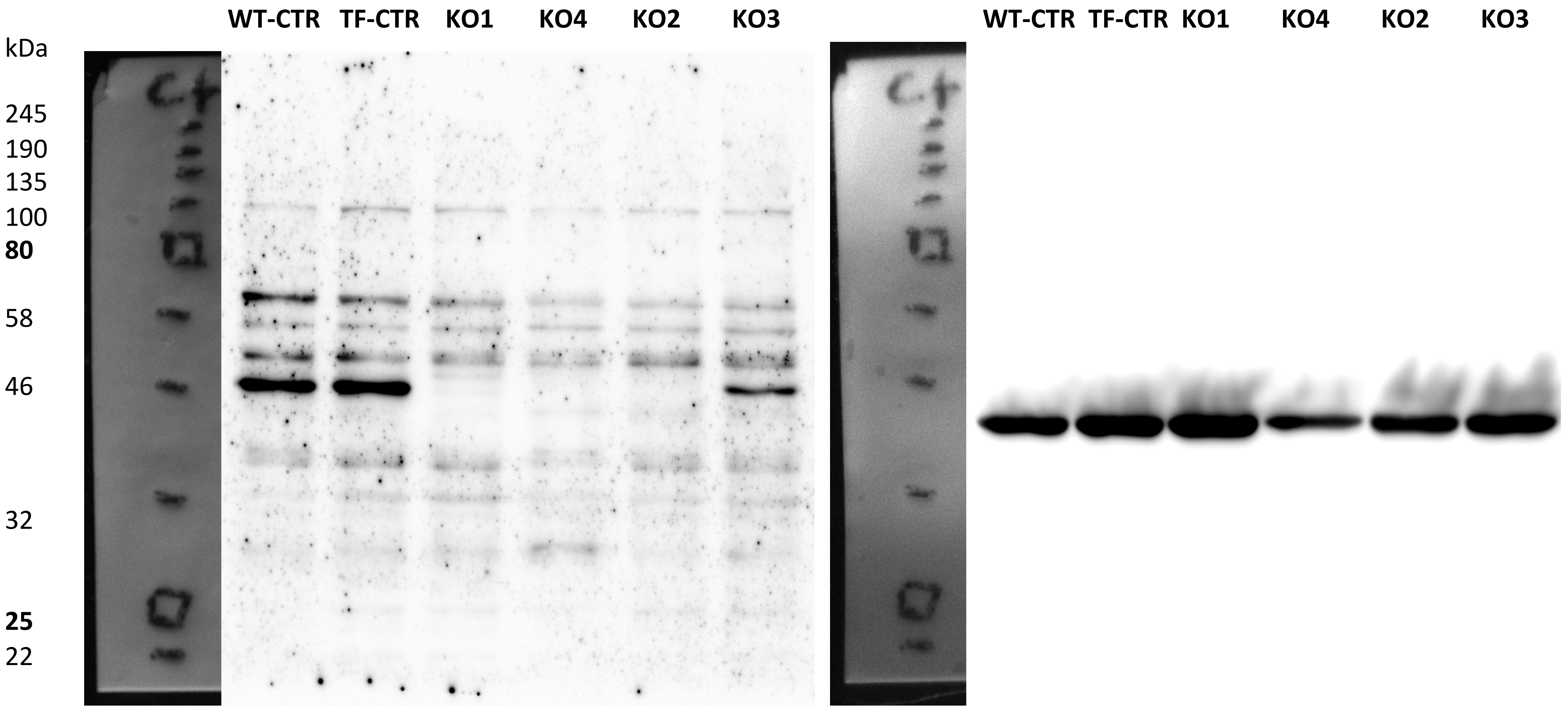

Full images of Western blots for PARP (Fig. 2c)

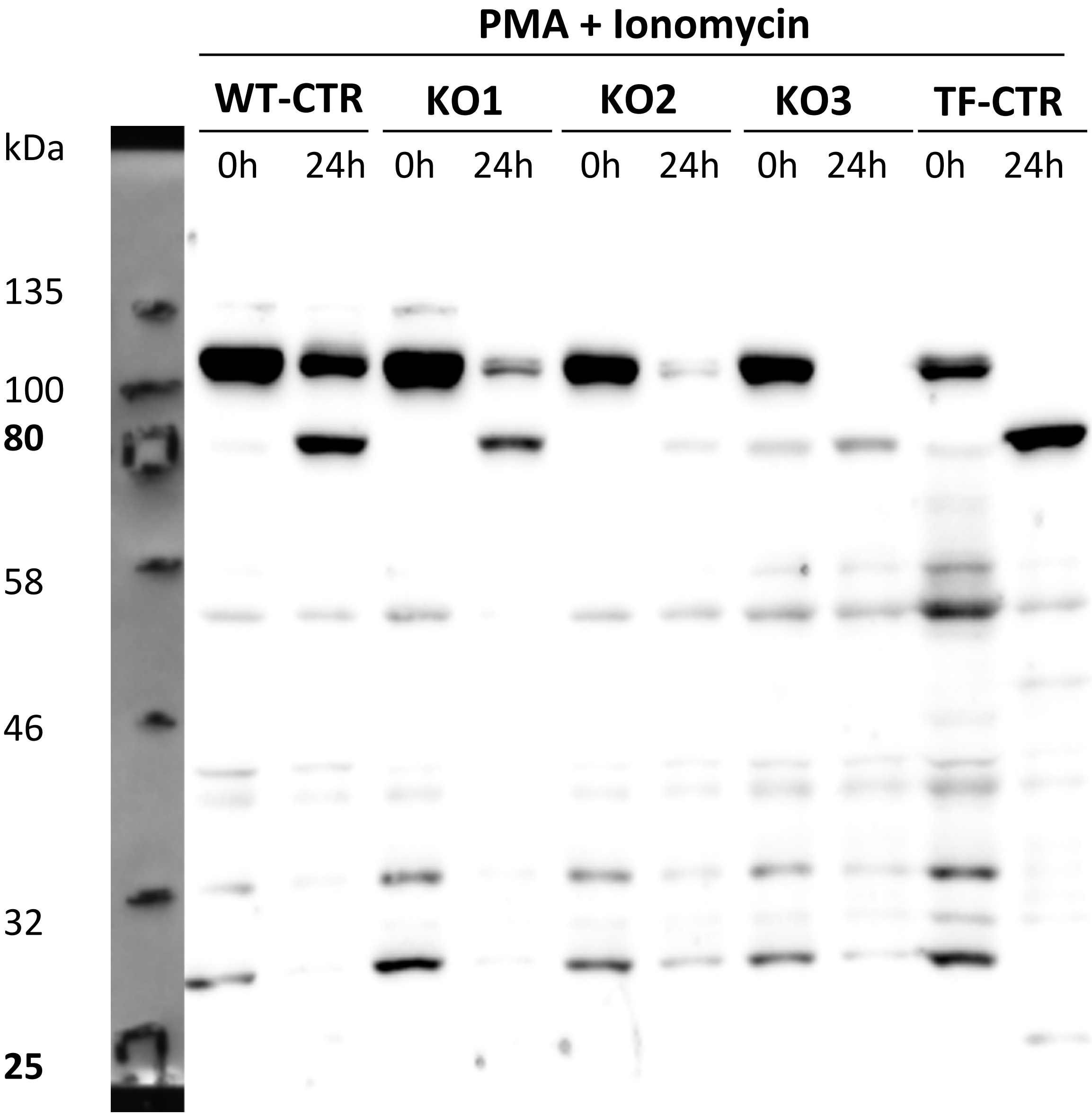

Full images of Western blots for Caspase-3 (Fig. 2c) with short exposure.

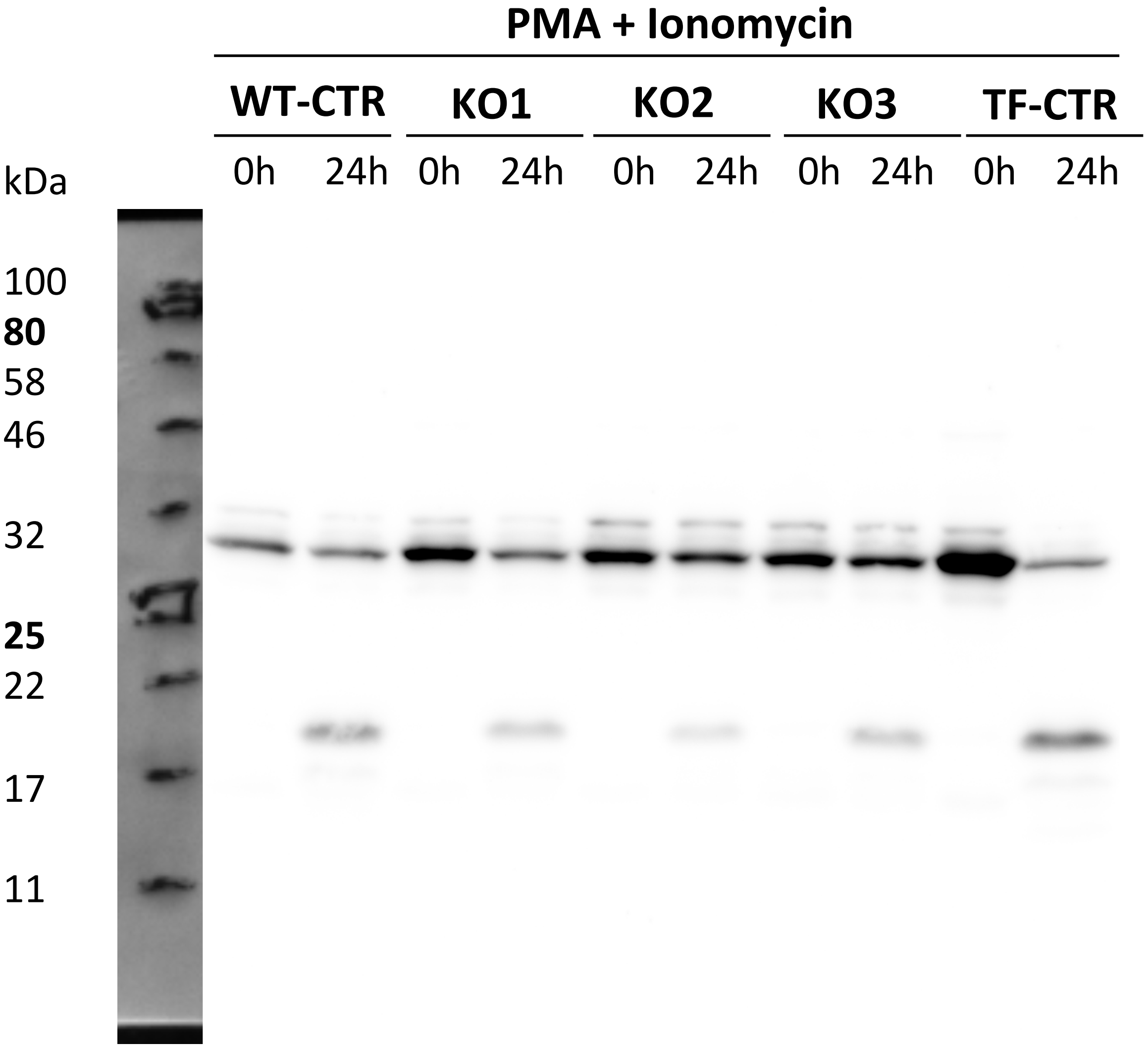

Full images of Western blots for Caspase-3 (Fig. 2c) with long exposure.

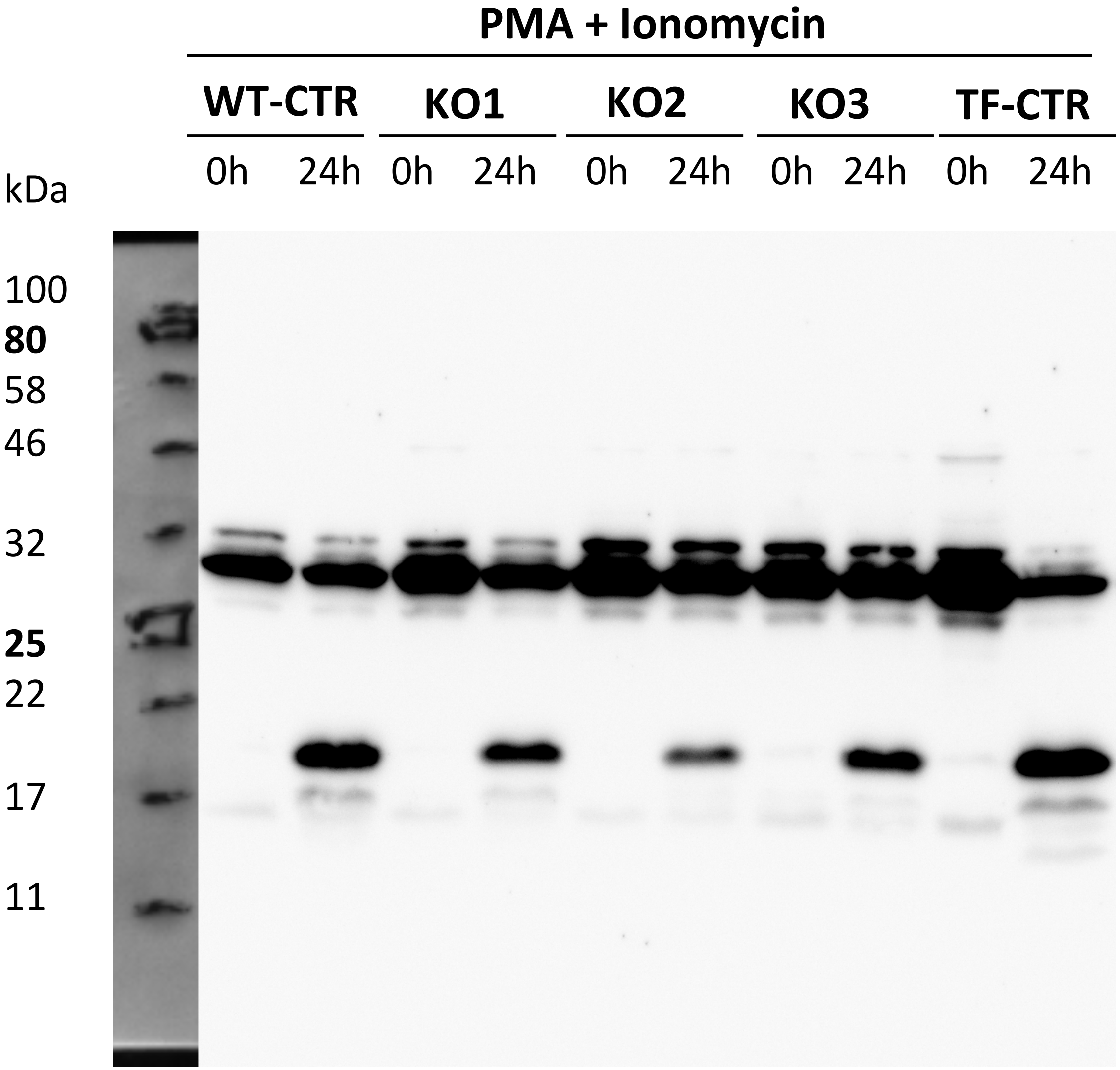

Full images of Western blots for cIAP-2 (Fig. 2c) and beta-actin (Fig. 2c)

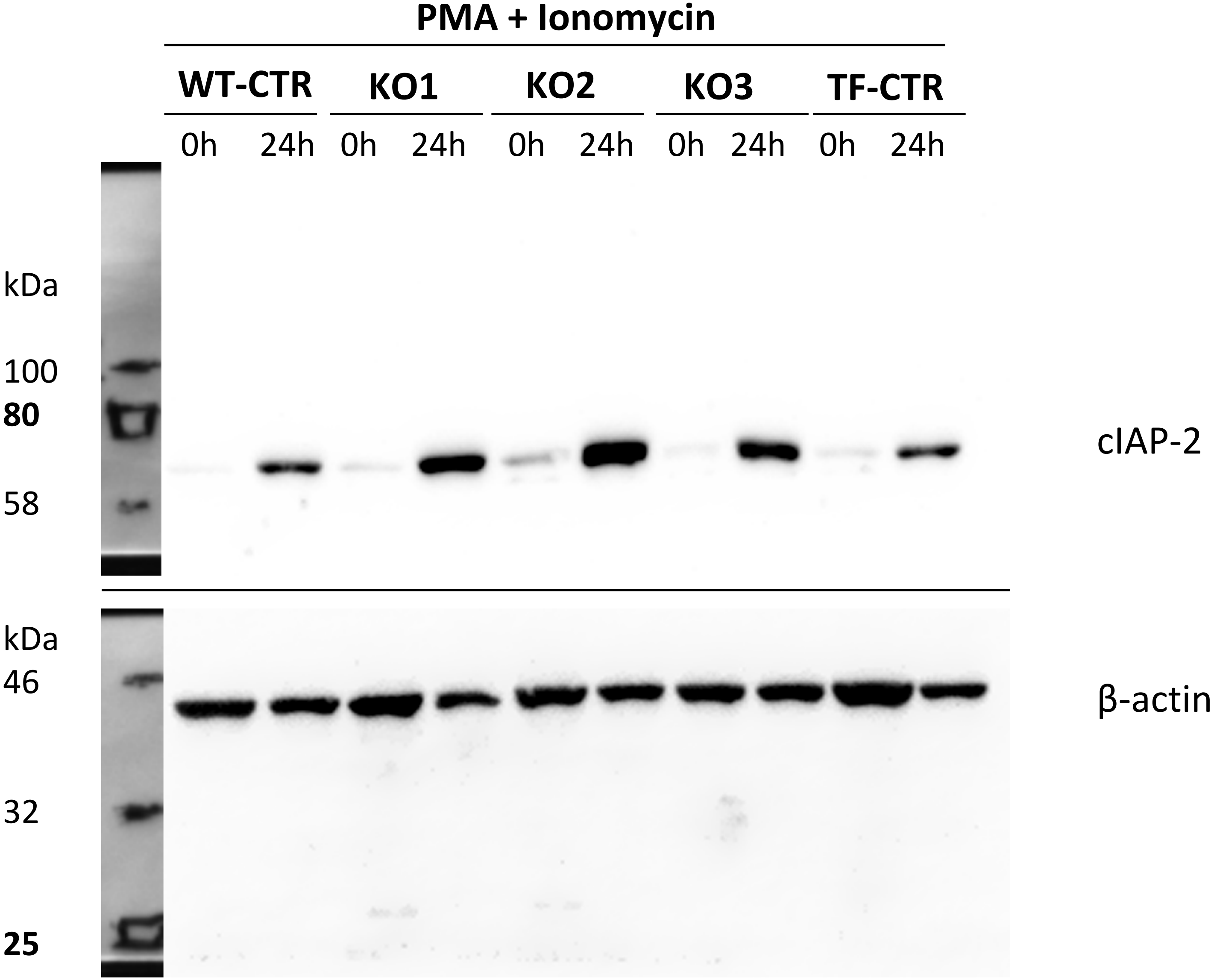

Full images of Western blots for Caspase-8 (Fig. 2d). The blot was cut in half after antibody incubation and parts were developed seperately. Upper halves are displayed here with short (upper) and long exposure (lower).

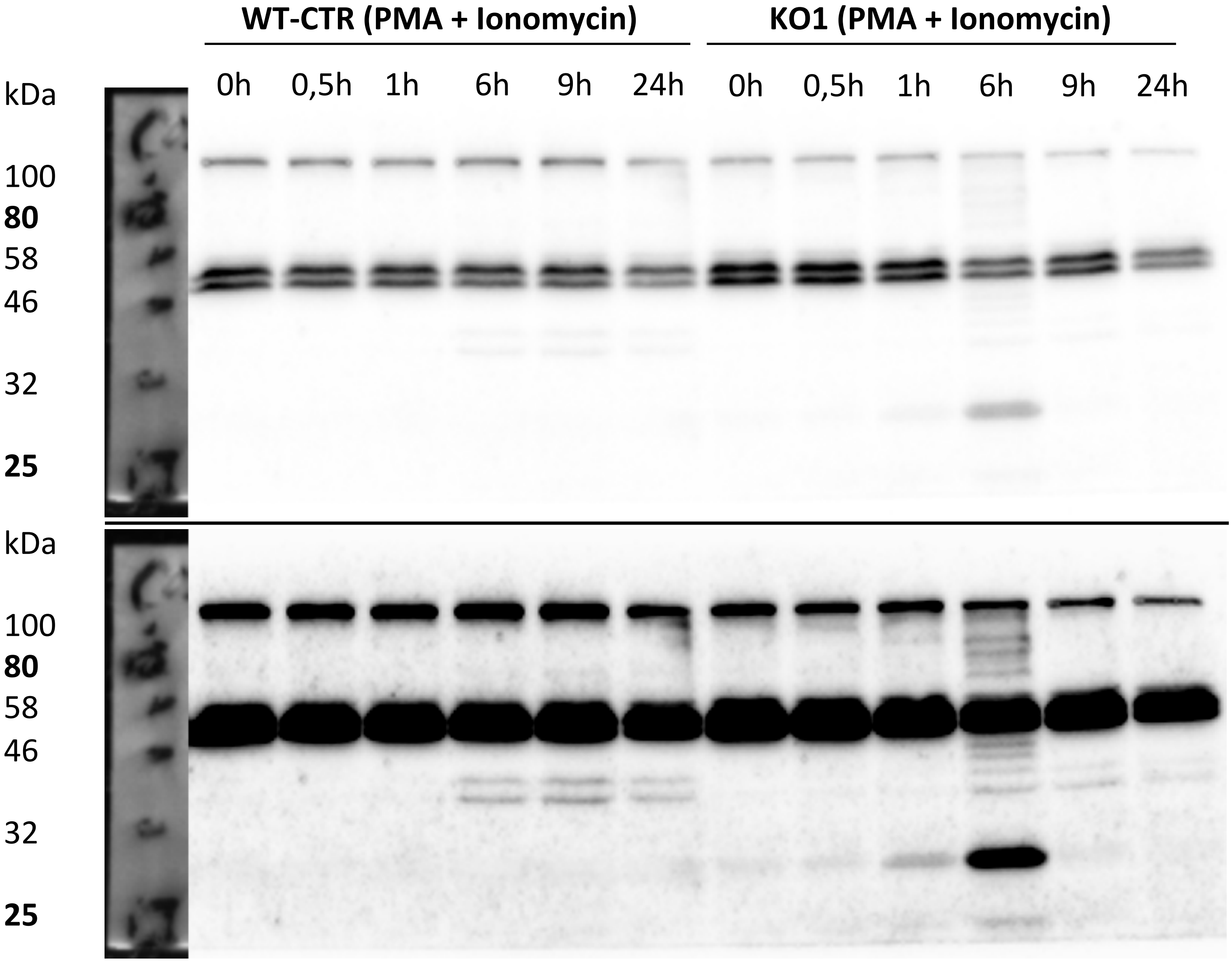

Full images of Western blots for Caspase-8 (upper) (Fig. 2d). The blot was cut in half after antibody incubation and parts were developed seperately. The lower half is displayed here.

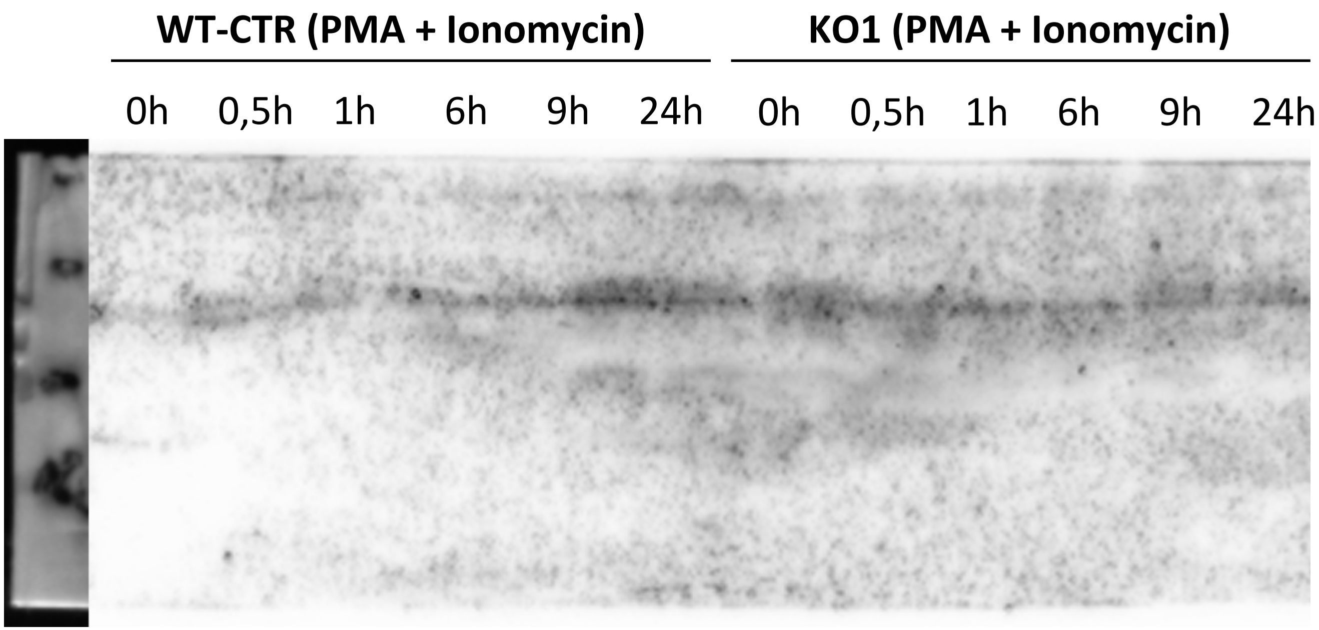

Full images of Western blots for β-actin (Fig. 2d).

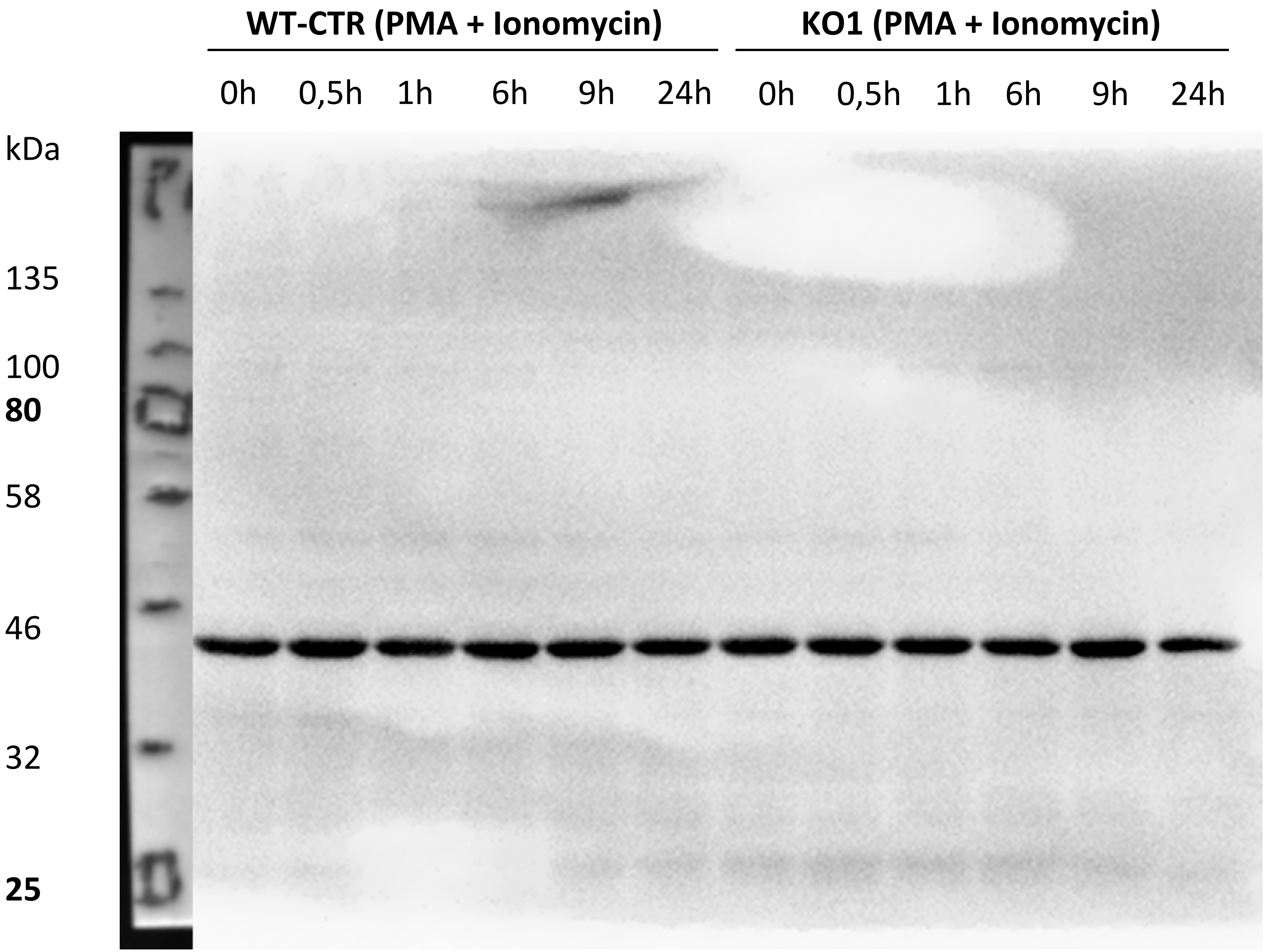

Full images of Western blots for Bip (Fig. 2e).

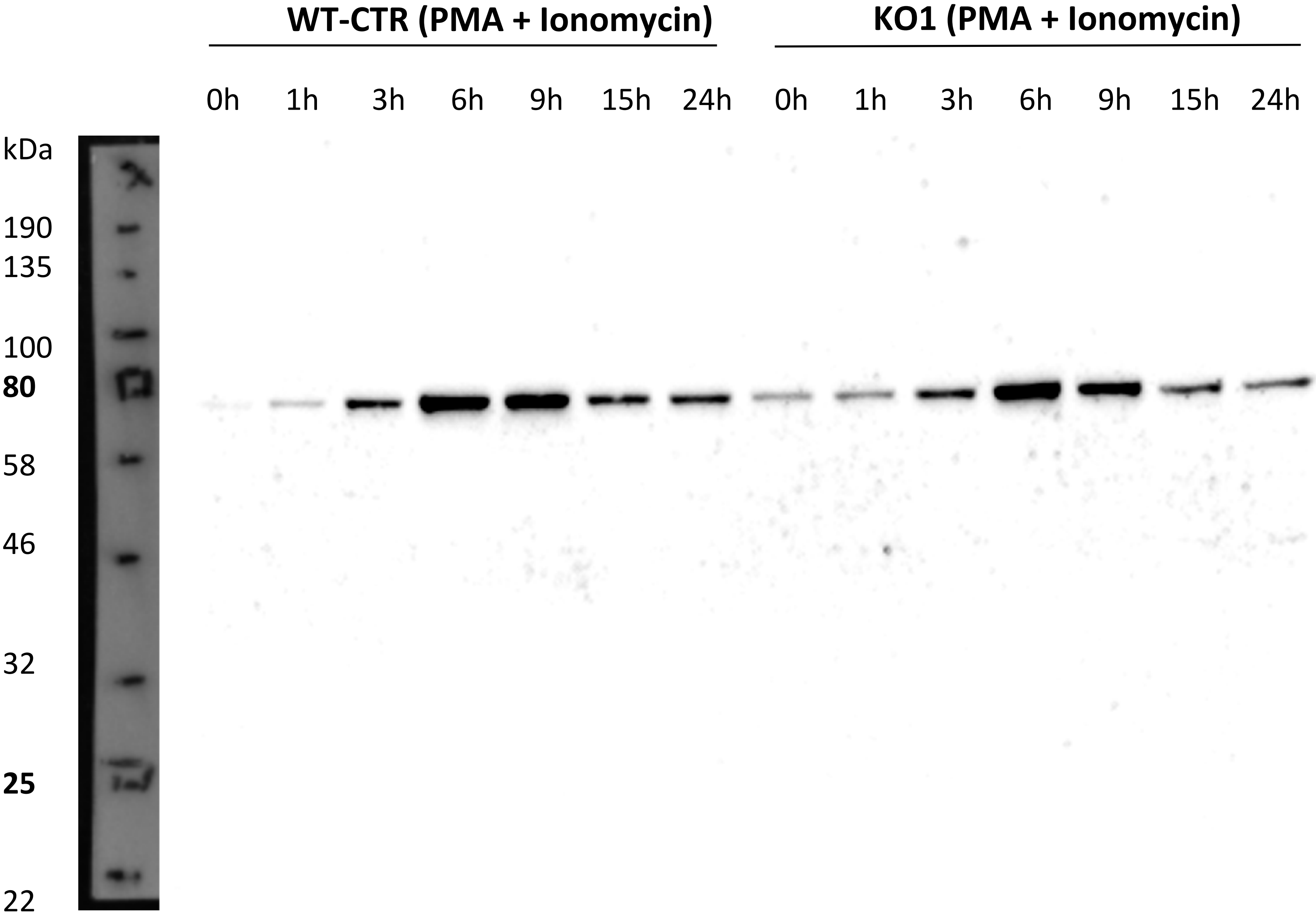

Full images of Western blots for CHOP (Fig. 2e).

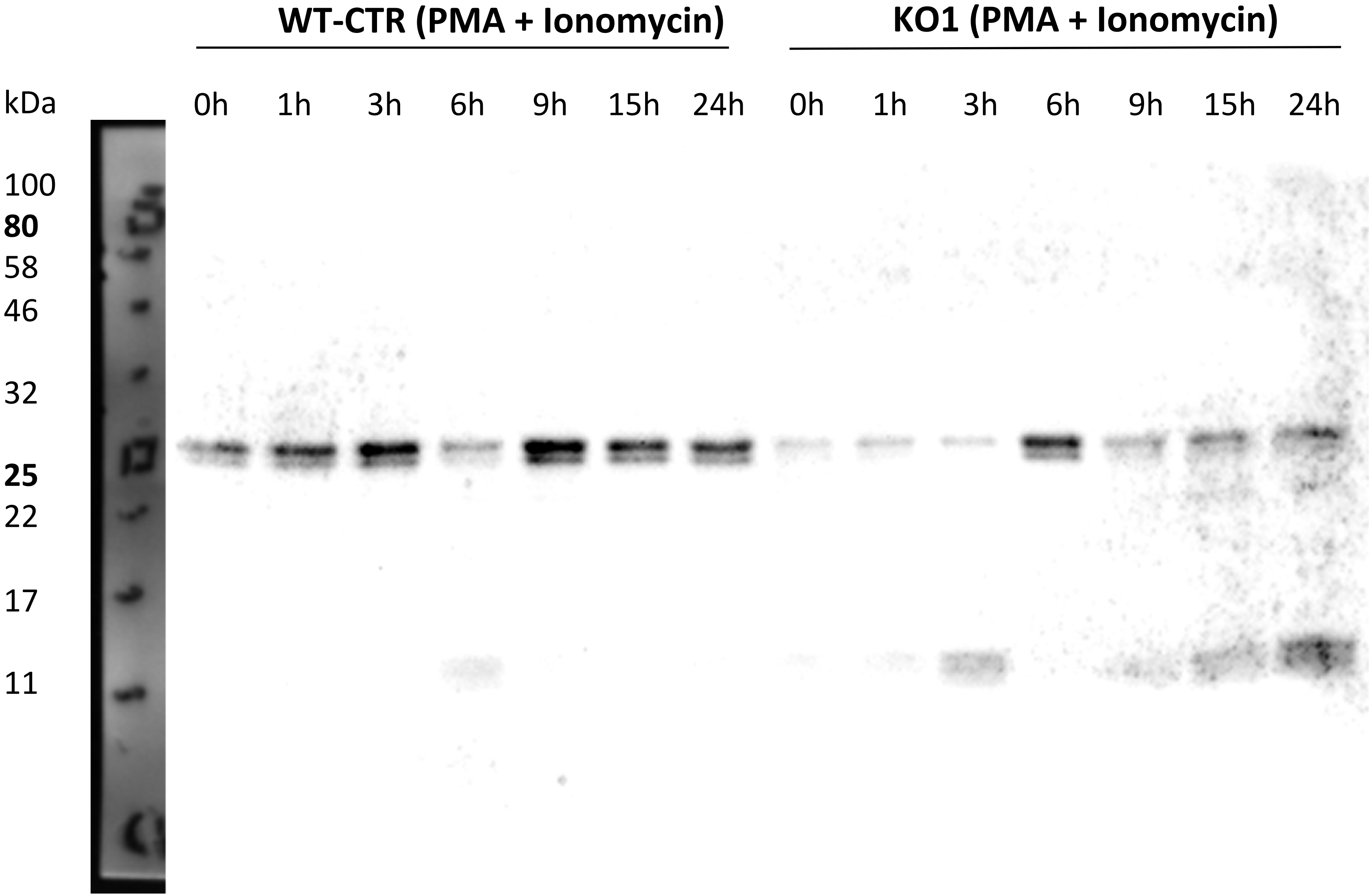

Full images of Western blots for β-actin (Fig. 2e).

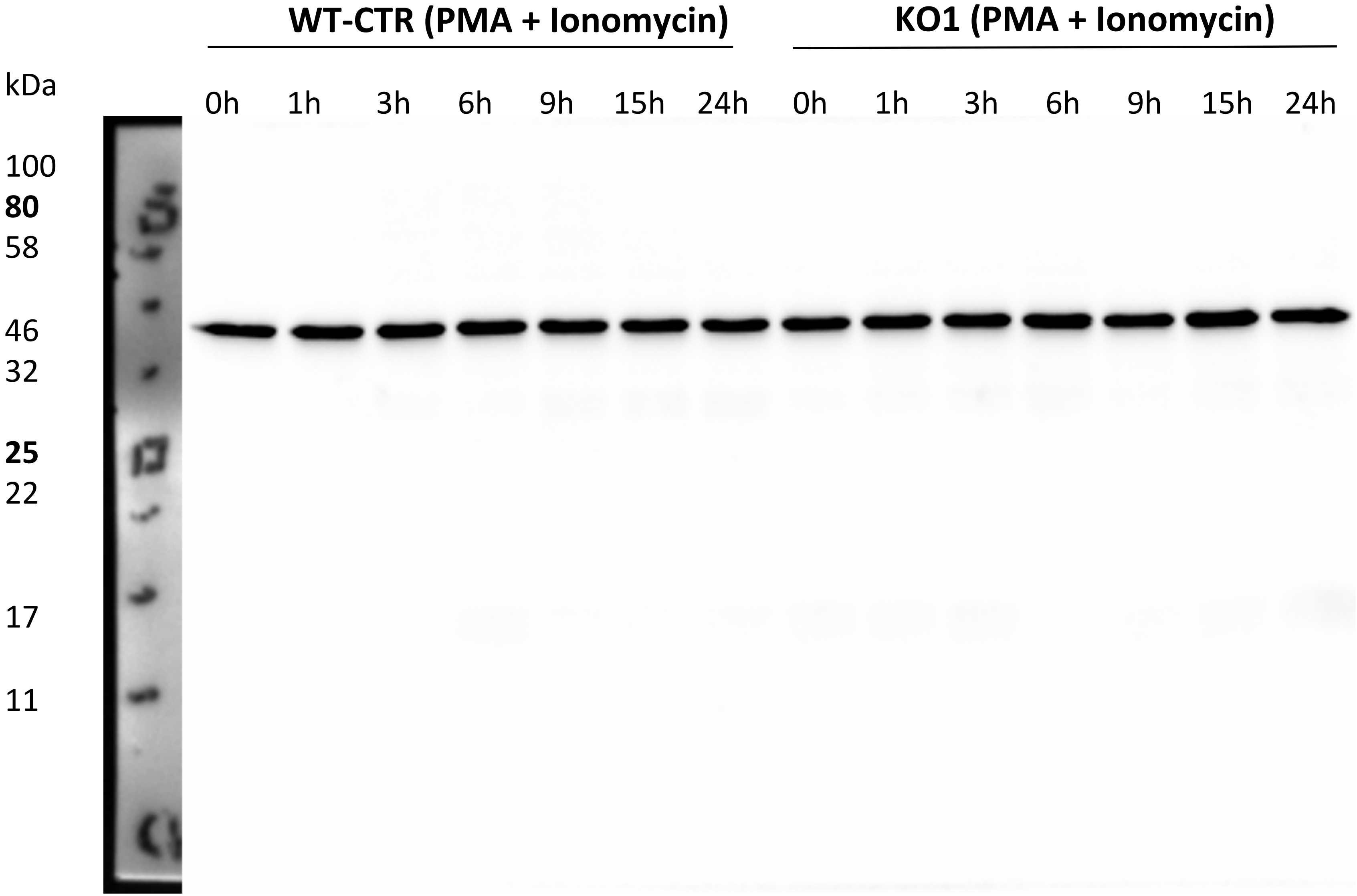

Western blot for Jnk (Jnk 1 = upper, Jnk 2 = lower band) as depicted in figure 2 f & g; 30min 3x3 binning

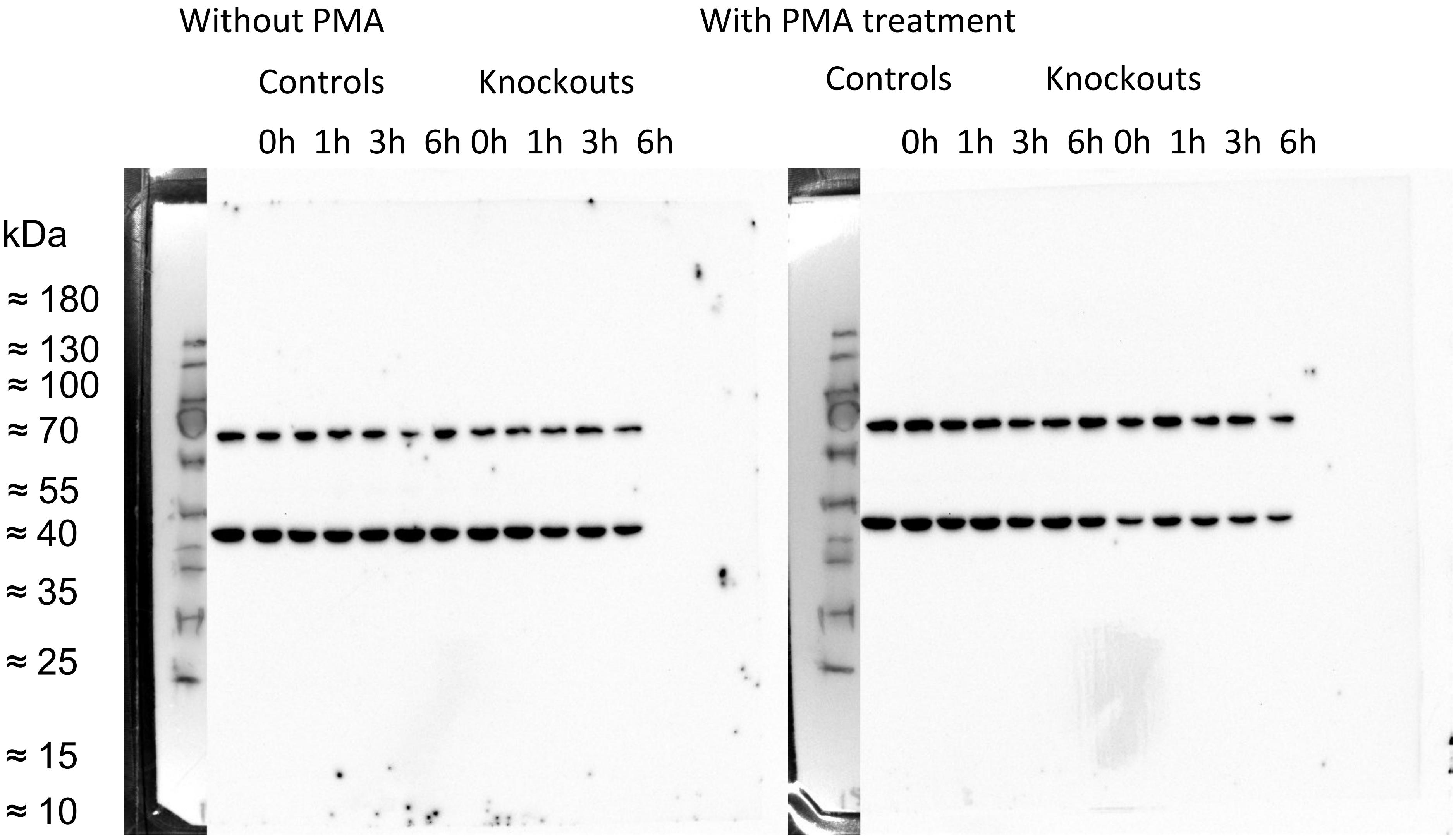

Western blot for betaActin as depicted in figure 2 f & g.
15min 3x3 binning

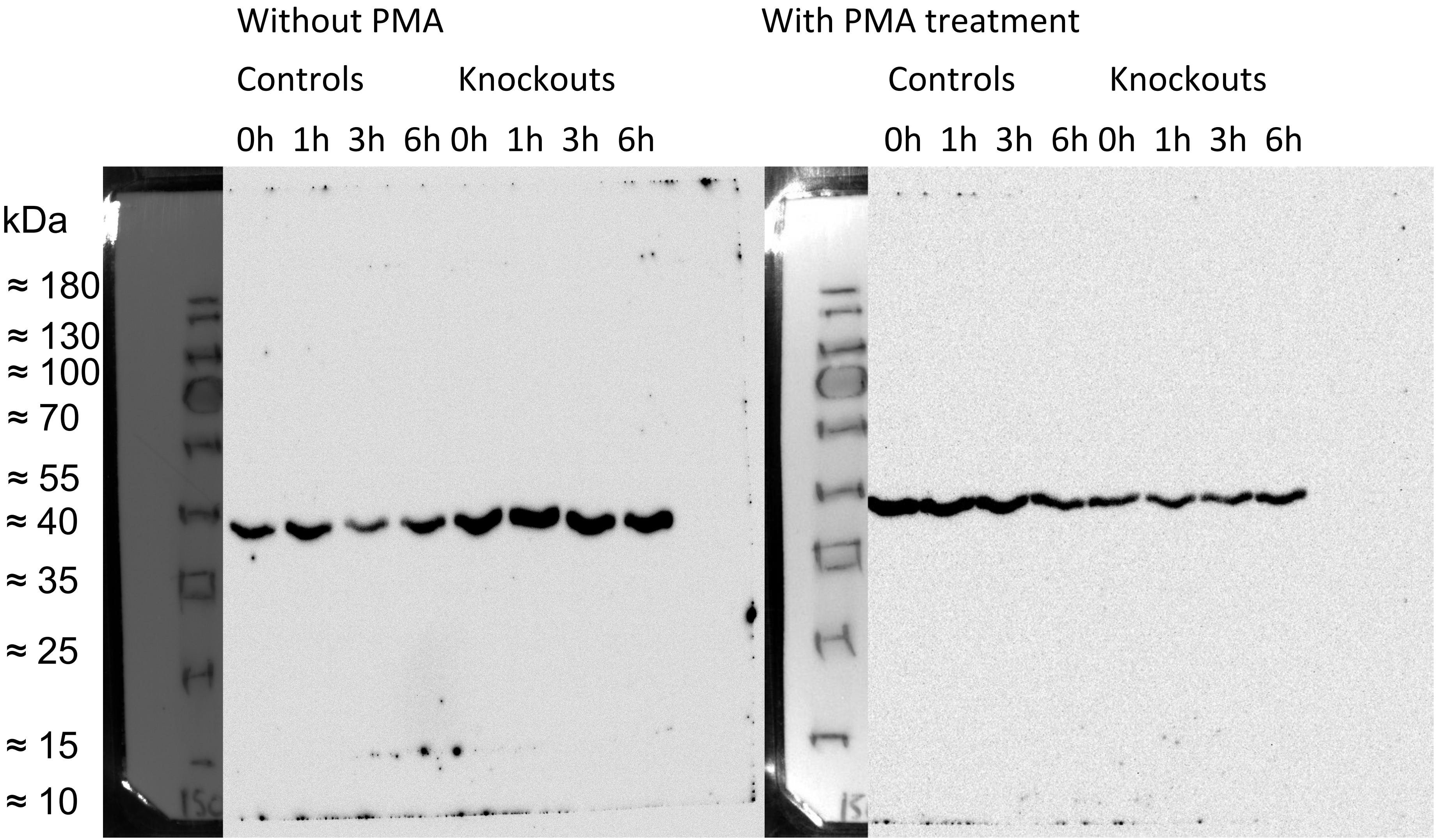

Western blot for Bim as depicted in figure 2 f & g.
30min 3x3 binning

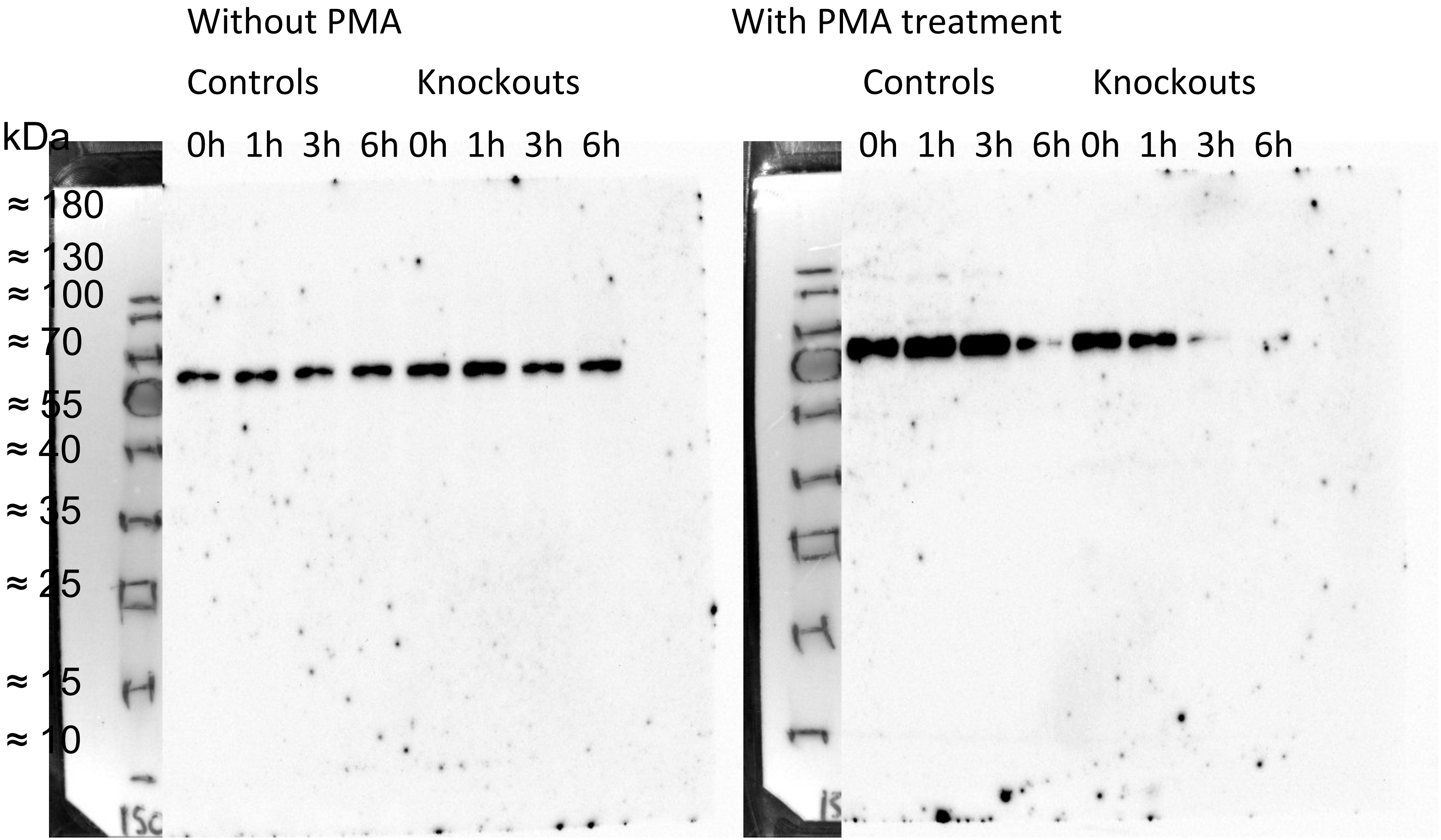

Western blot for phospho-Jnk depicted in figure 2 f & g.
30min 3x3 binning

Without PMA — With PMA treatment

Controls — Knockouts — Controls — Knockouts

0h 1h 3h 6h 0h 1h 3h 6h — 0h 1h 3h 6h 0h 1h 3h 6h

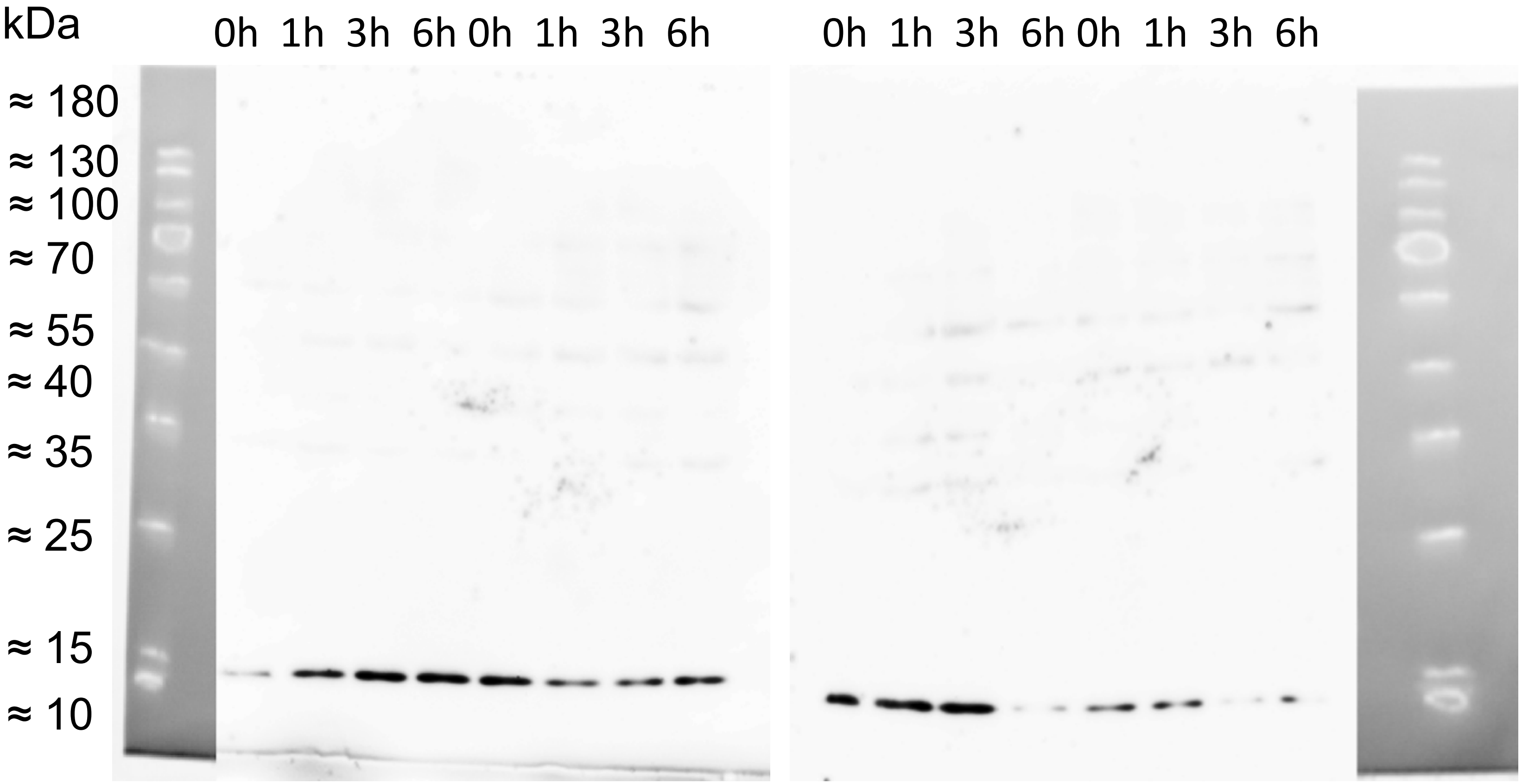

Full images of Western blots for cIAP-1 (Fig. 2k).

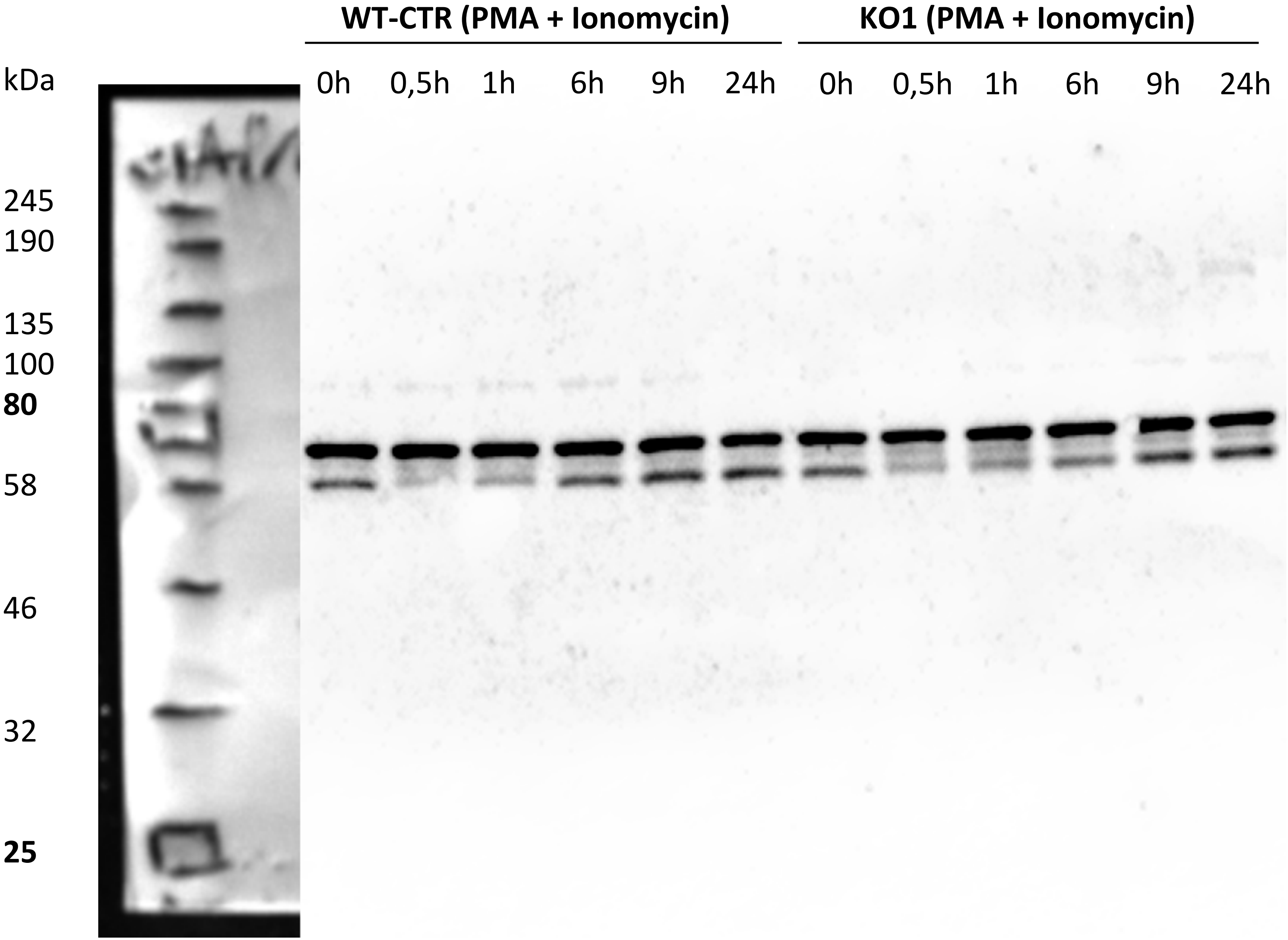

Full images of Western blots for β-actin (Fig. 2k).

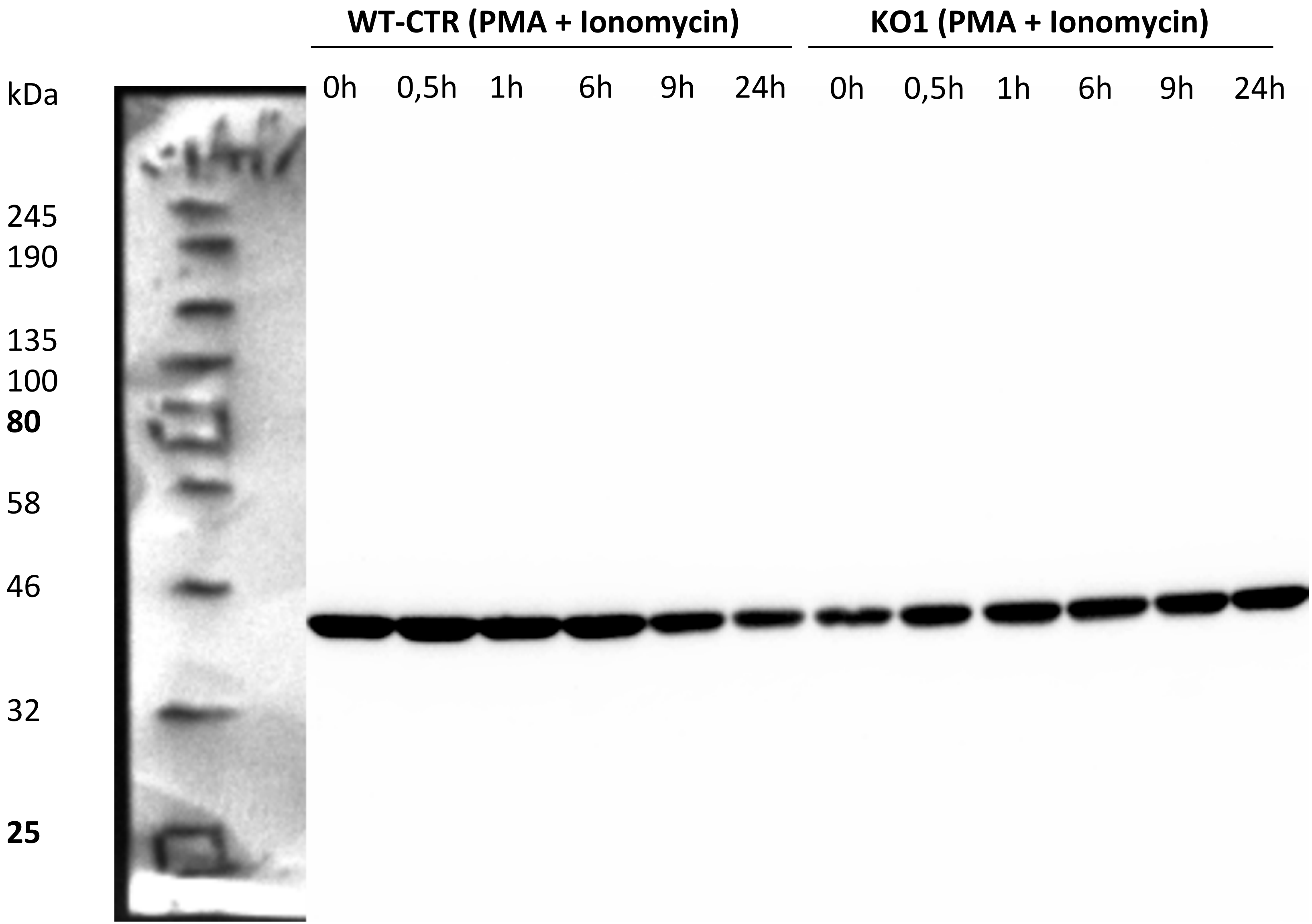

Full images of Western blots for phospho-mTOR (Fig. 3h).

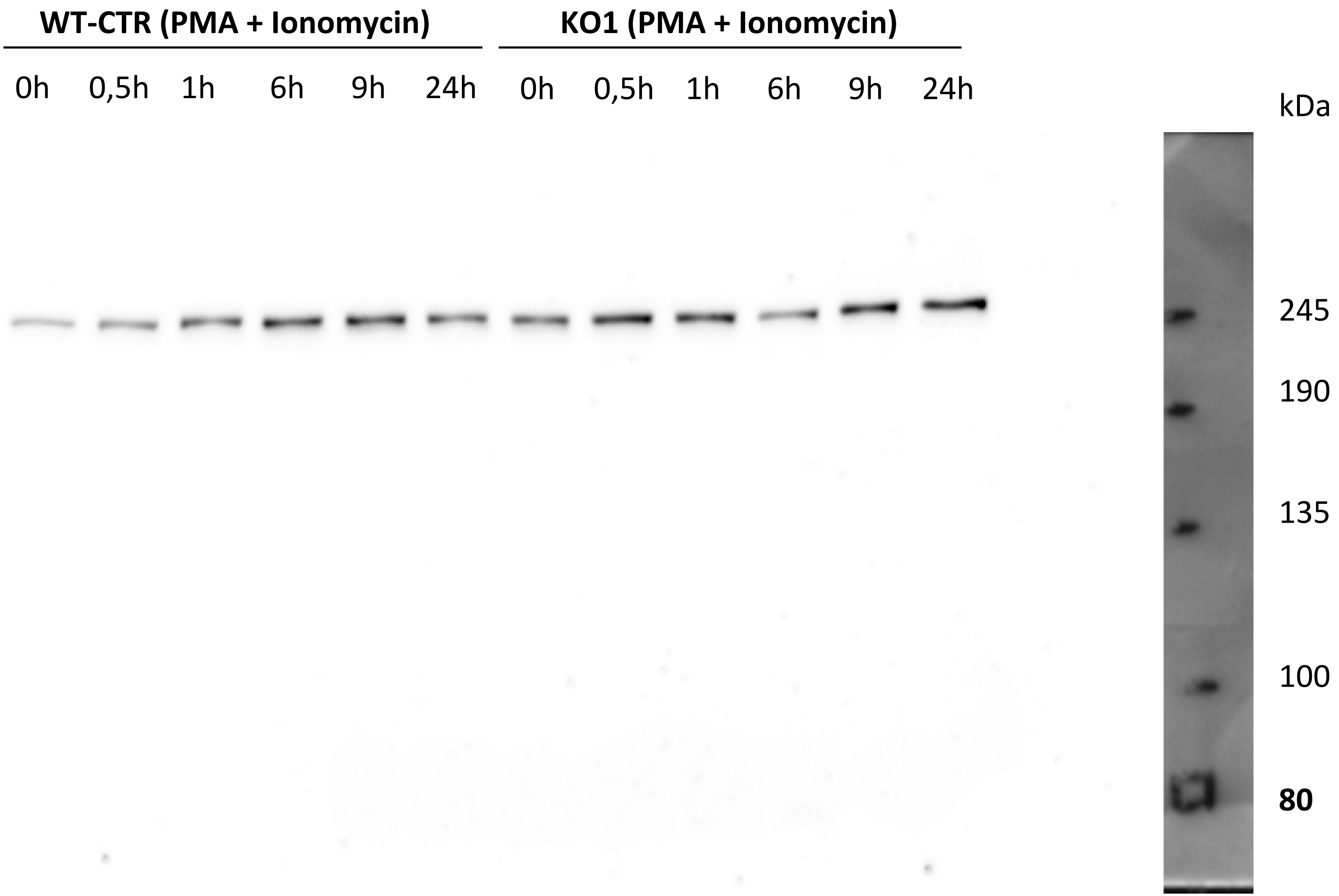

Full images of Western blots for mTOR (Fig. 3h).

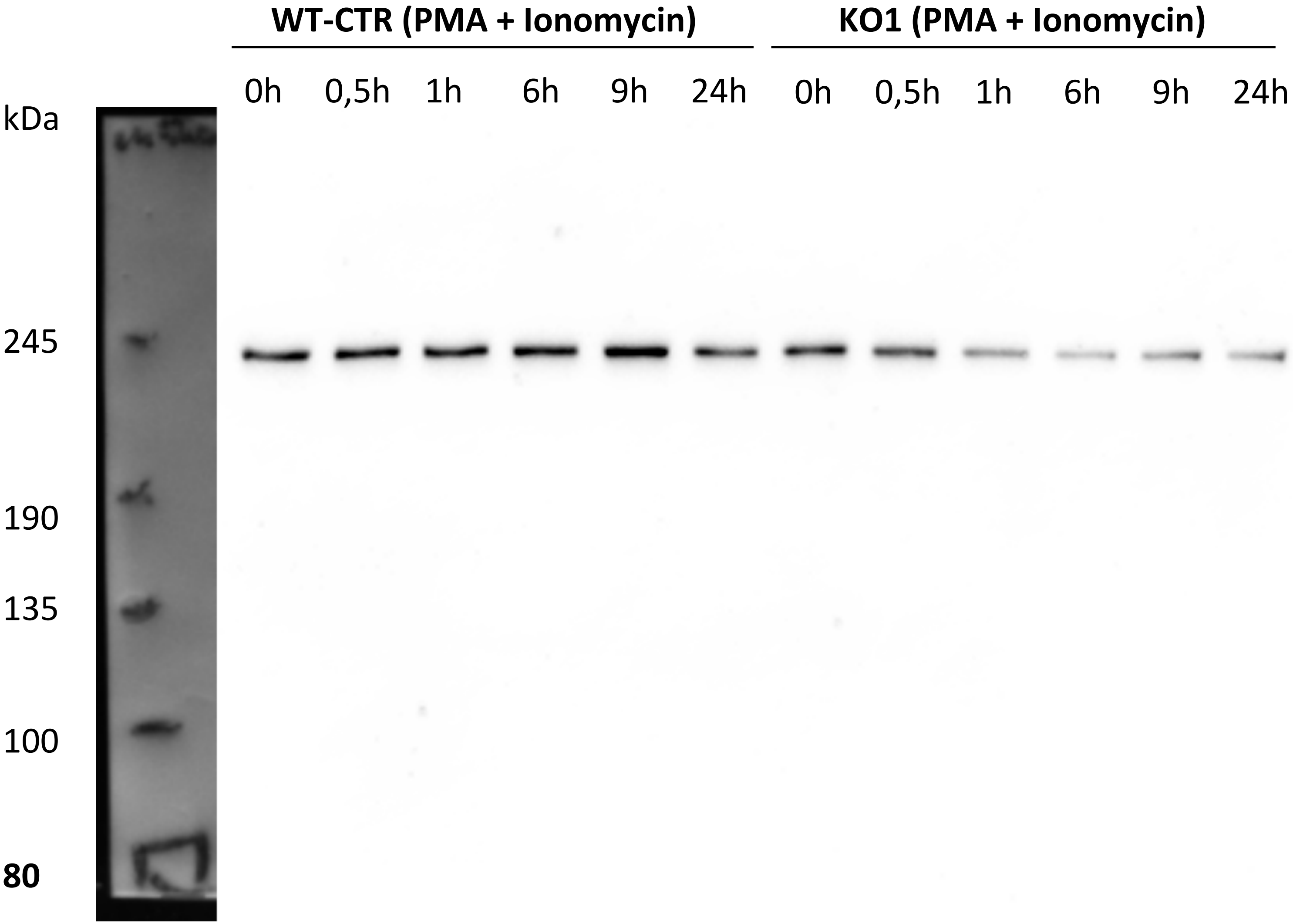

Full images of Western blots for phospho-S6-kinase (pS6K) on top and phospho-RibS6 (pRibS6) on bottom (Fig. 3i).

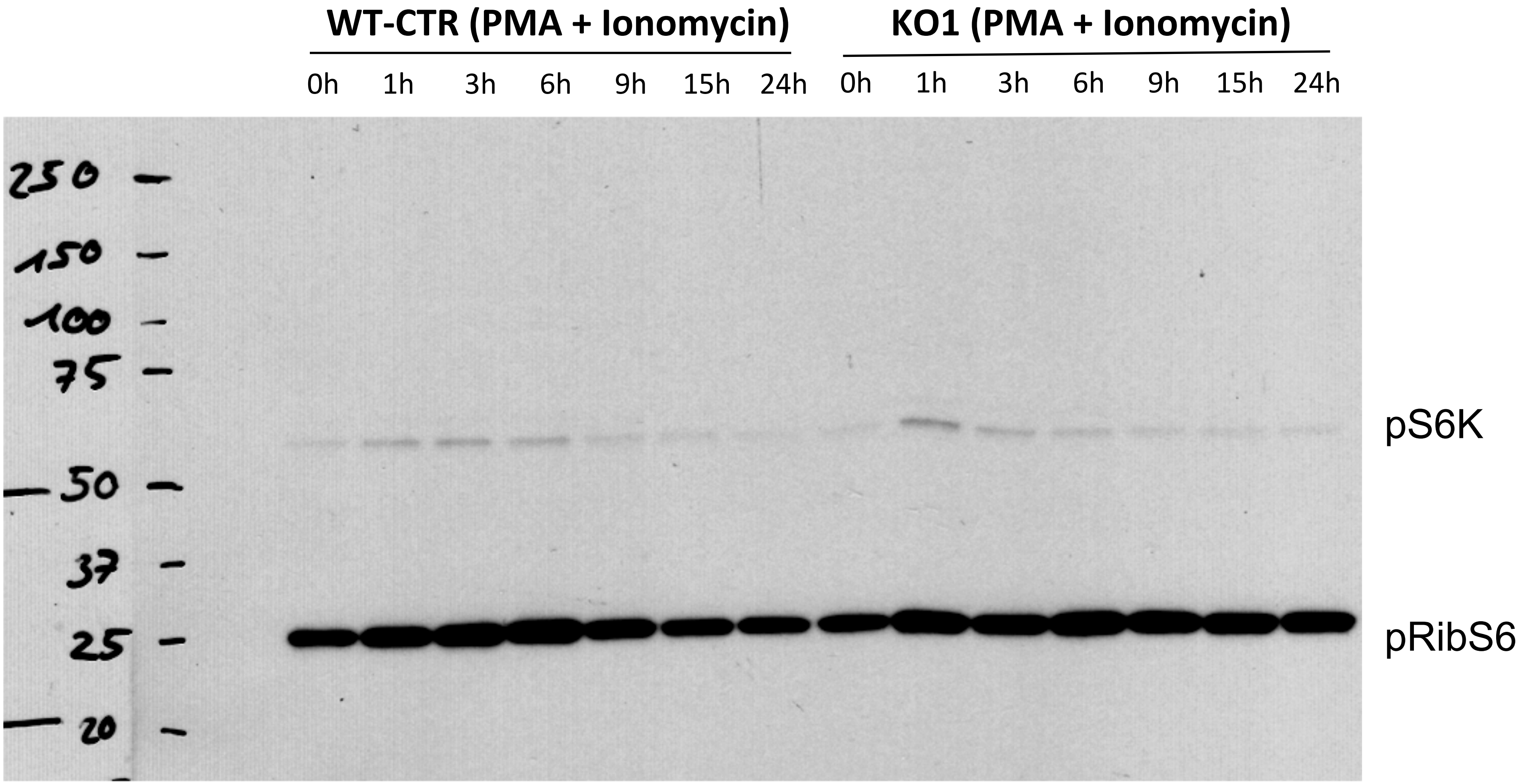

Full images of Western blots for O-GlcNAc residues on proteins (Fig. 4c).

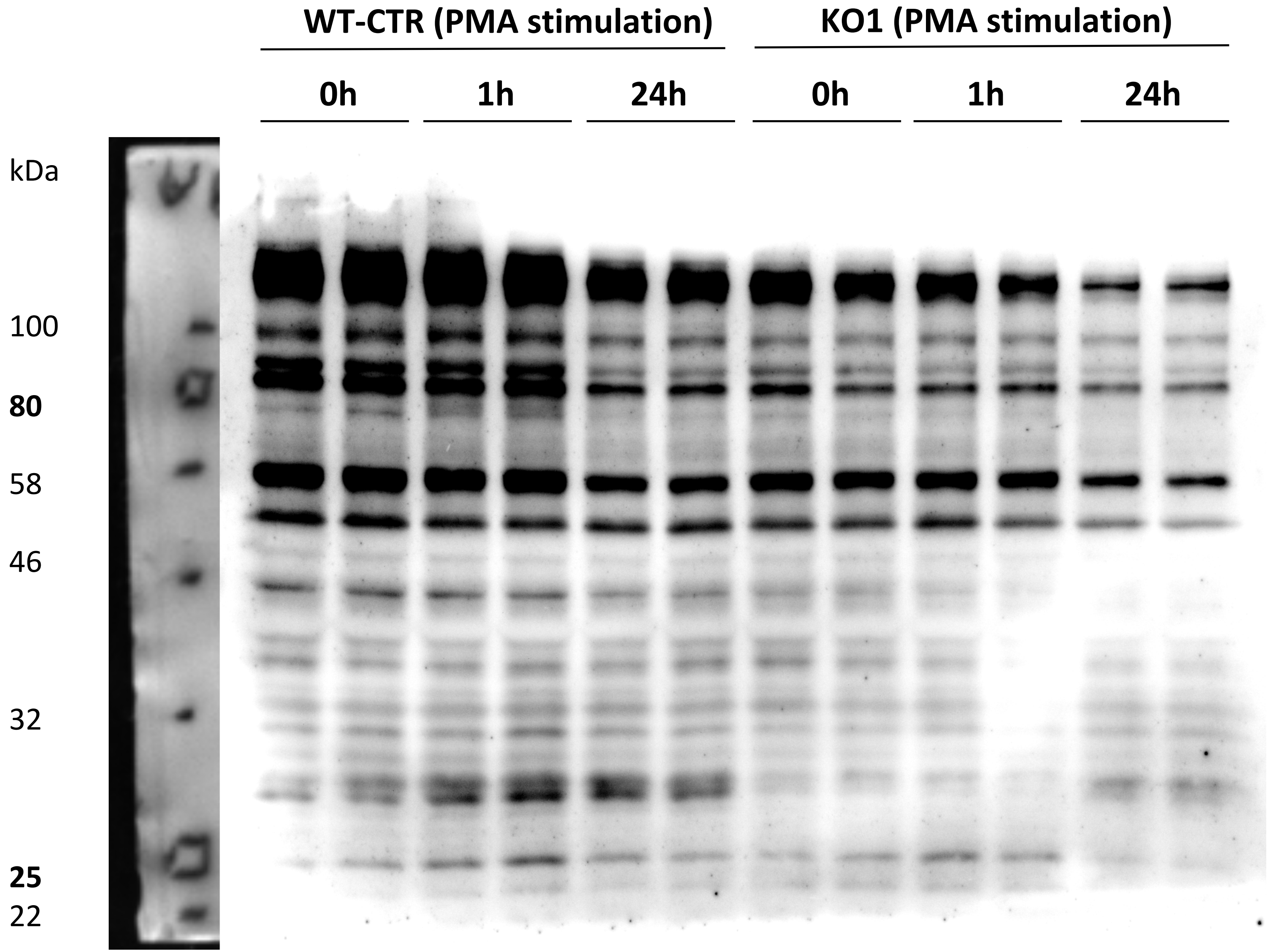

Full images of Western blots for β-actin (Fig. 4c). Additional bands are residues of the anti-O-GlcNAc prior to stripping the membrane.

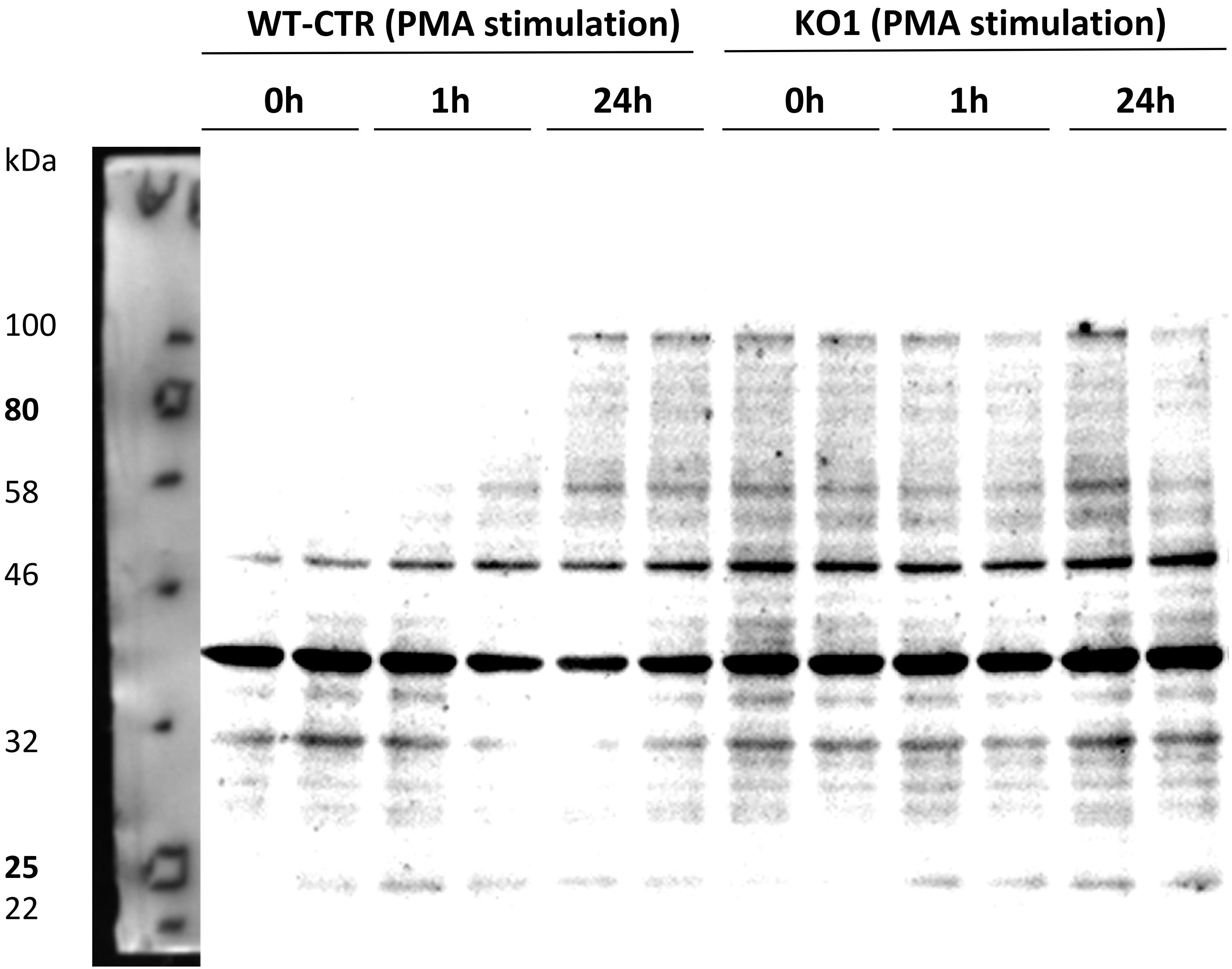

Full images of Western blots for α- or β-linked terminal *N*-acetylgalactosamine with VVL (Fig. 4d). Additional bands are residues of the anti-O-GlcNAc prior to stripping the membrane.

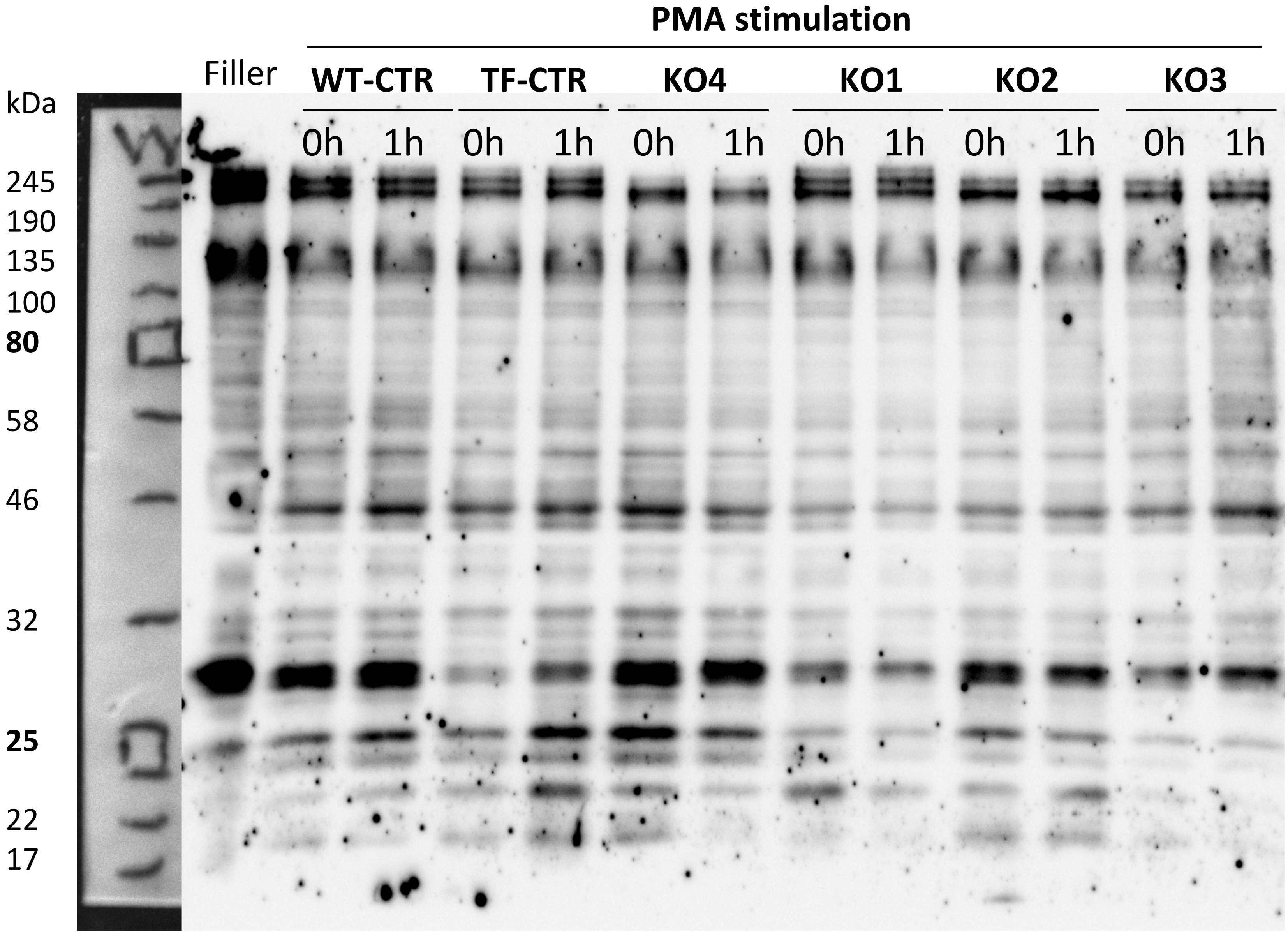

Full images of Western blots for β-actin (Fig. 4d).

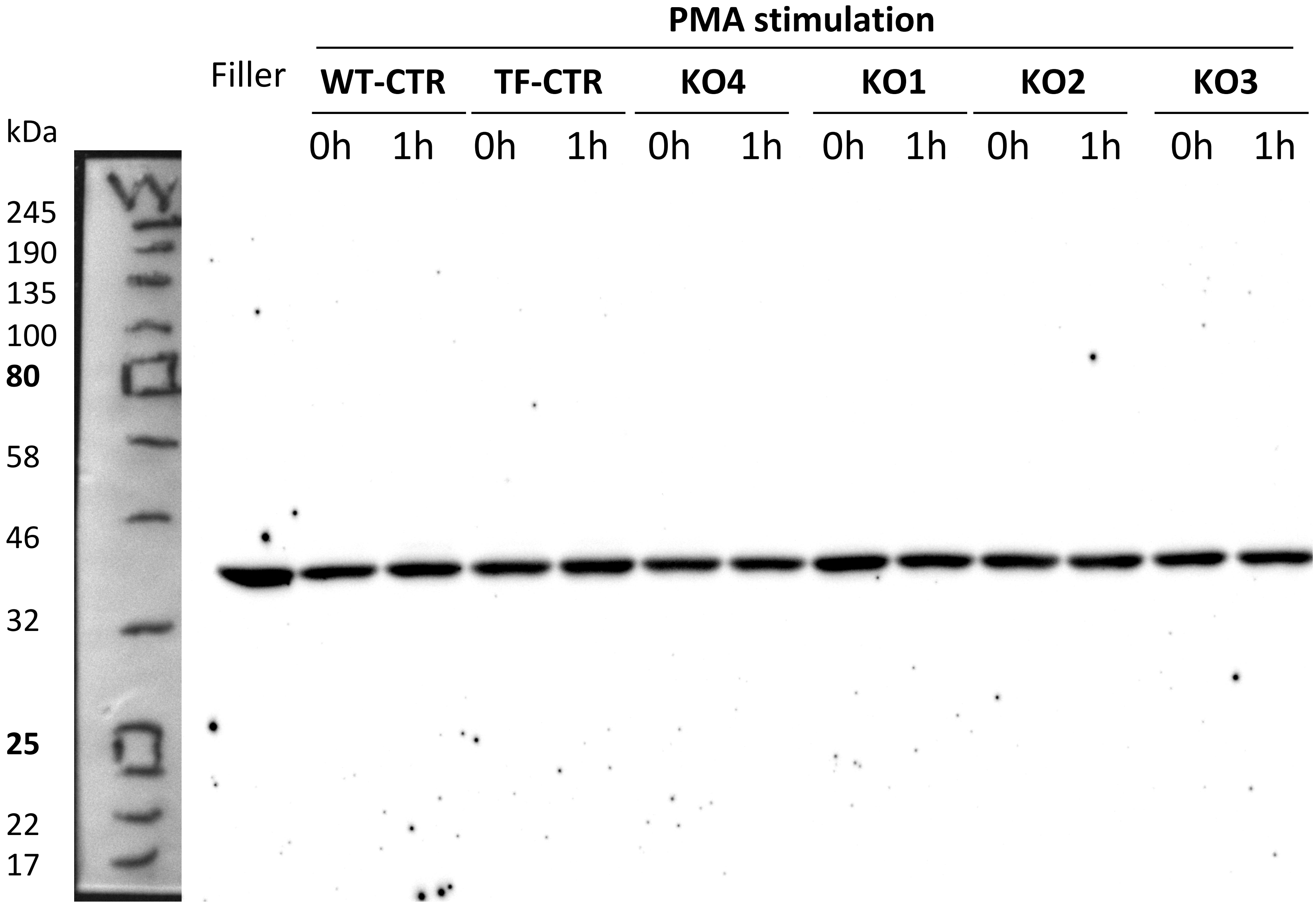

Full images of Western blots for ADPGK (Fig. 5b), size appropriate.

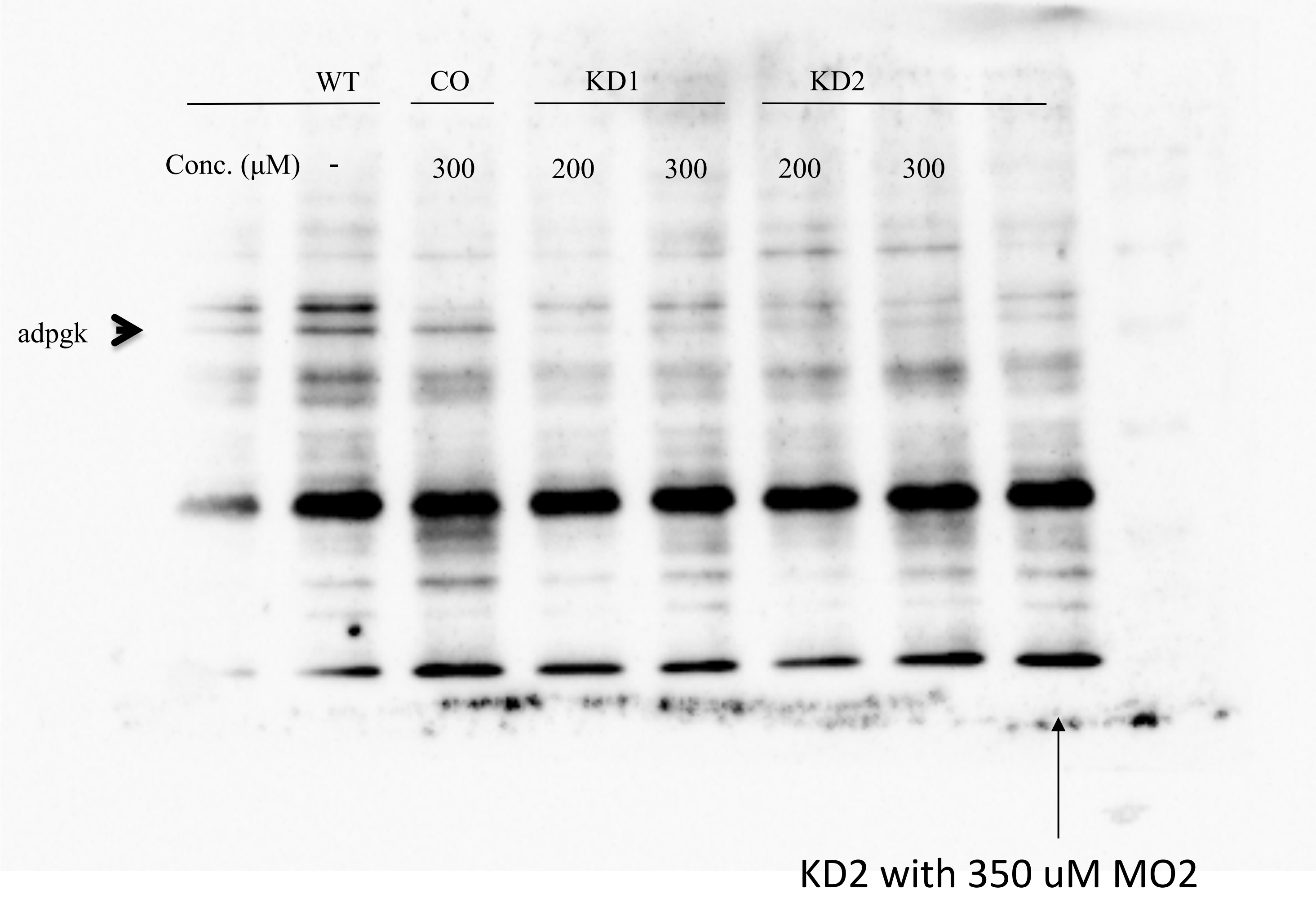

Full images of Western blots for Actin (Fig. 5b), , size appropriate.

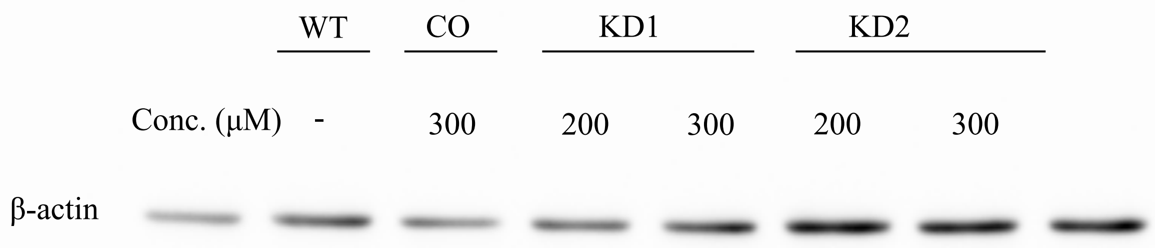

Full images of Western blots for PARP (Suppl. Fig. 1 h).

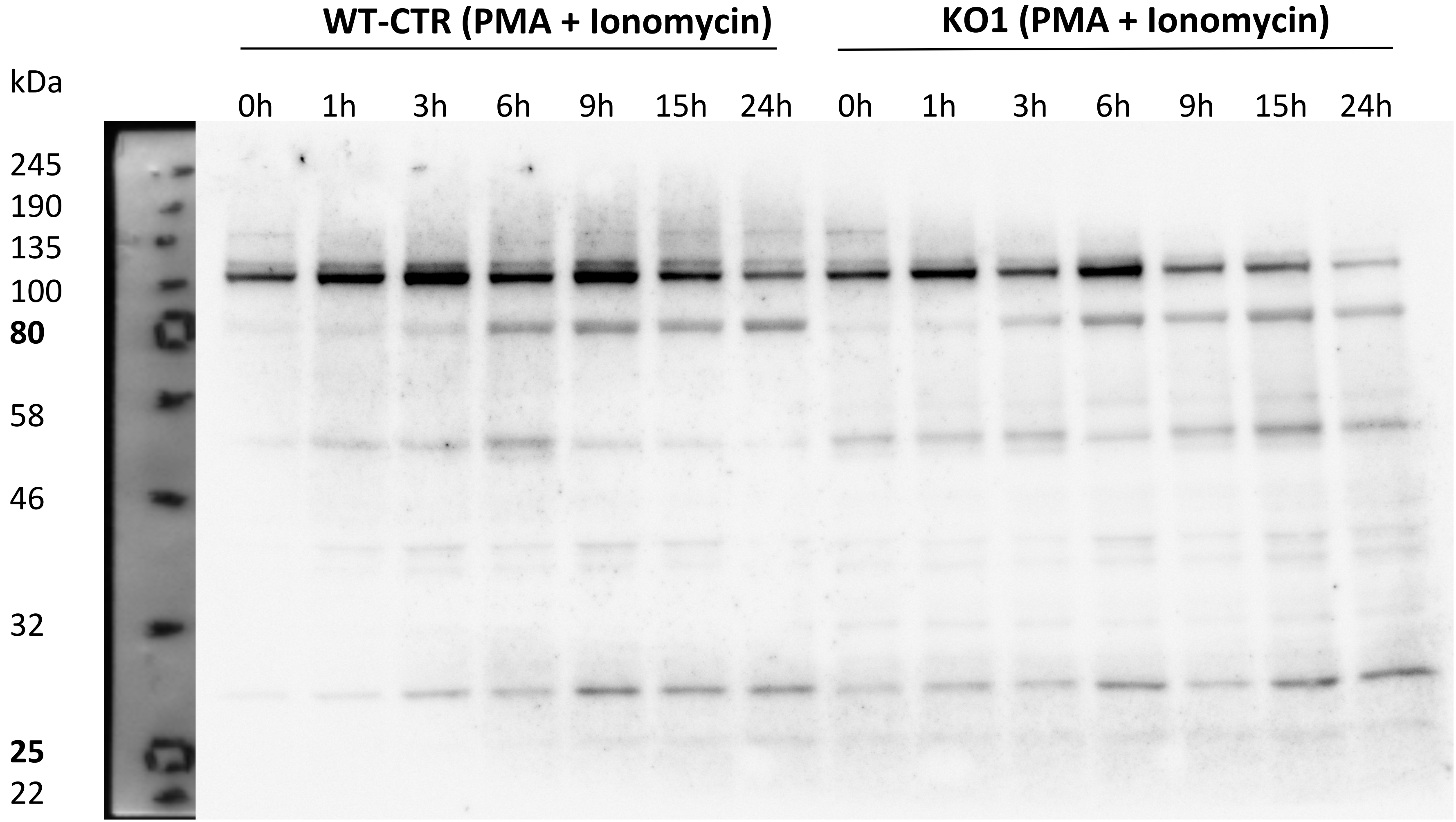

Full images of Western blots for Caspase-3 (Suppl. Fig. 1 h) with short exposure.

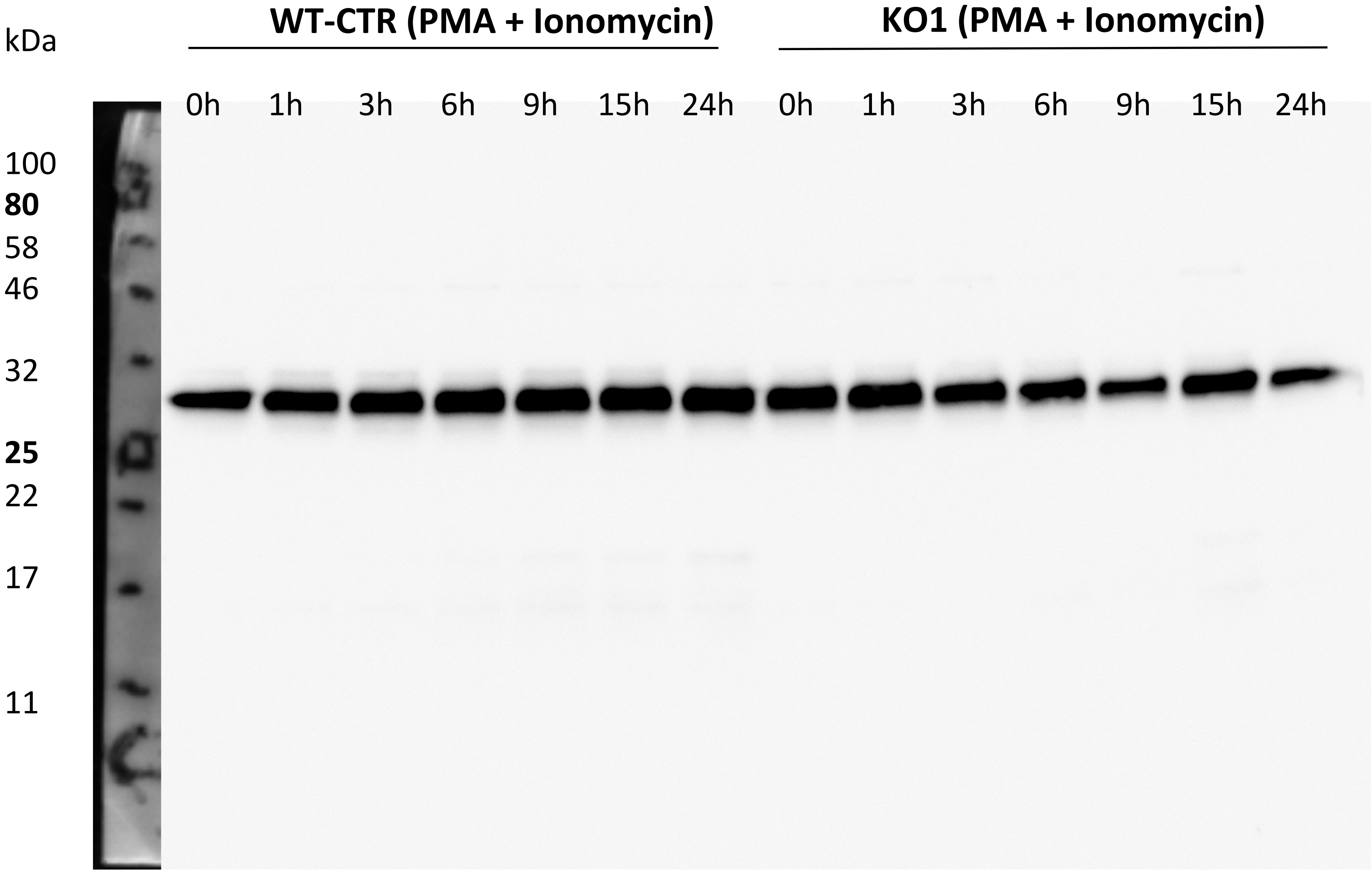

Full images of Western blots for Caspase-3 (Suppl. Fig. 1 h) with long exposure.

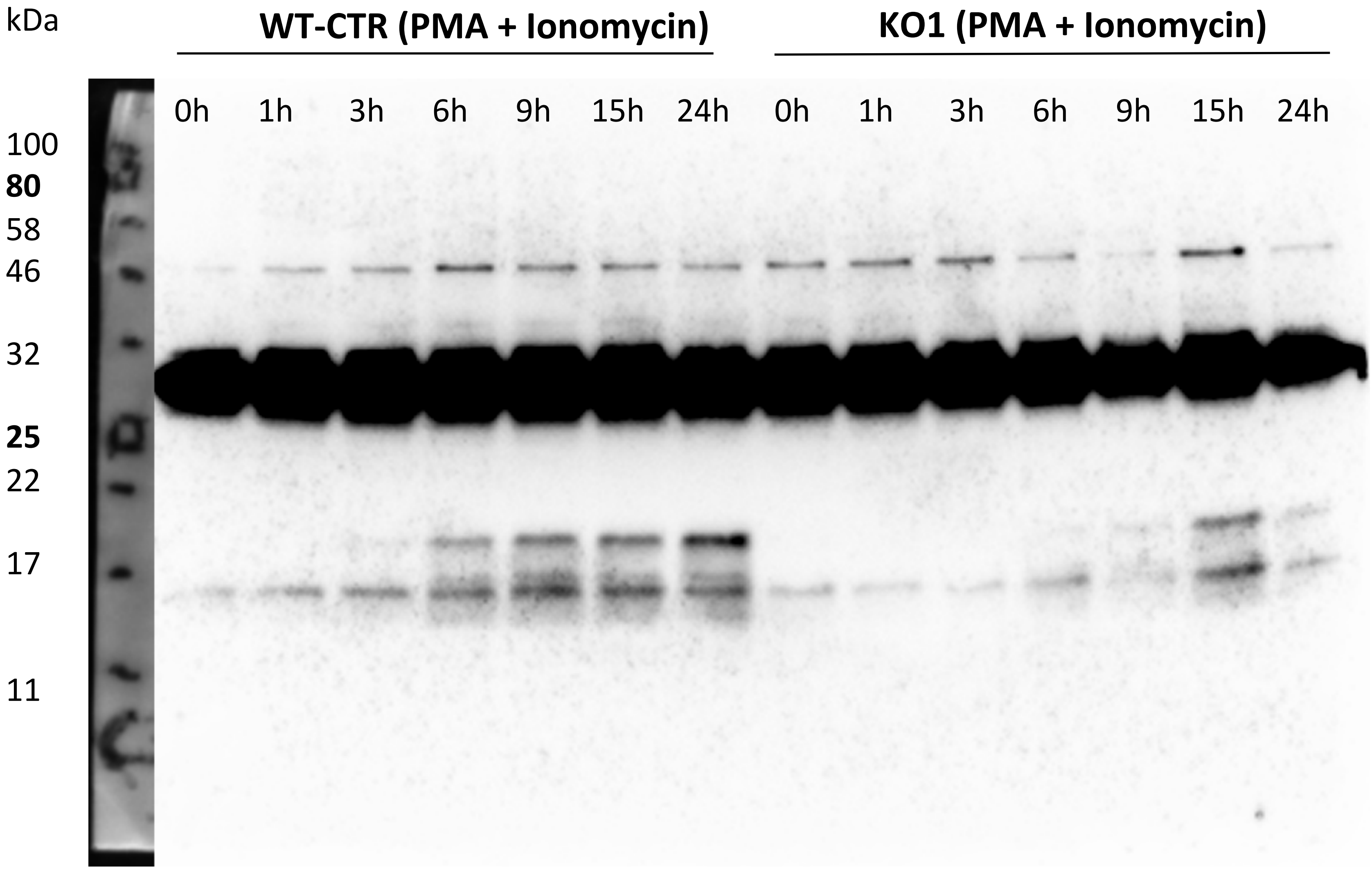

Full images of Western blots for cIAP-2 (Suppl. Fig. 1 h).

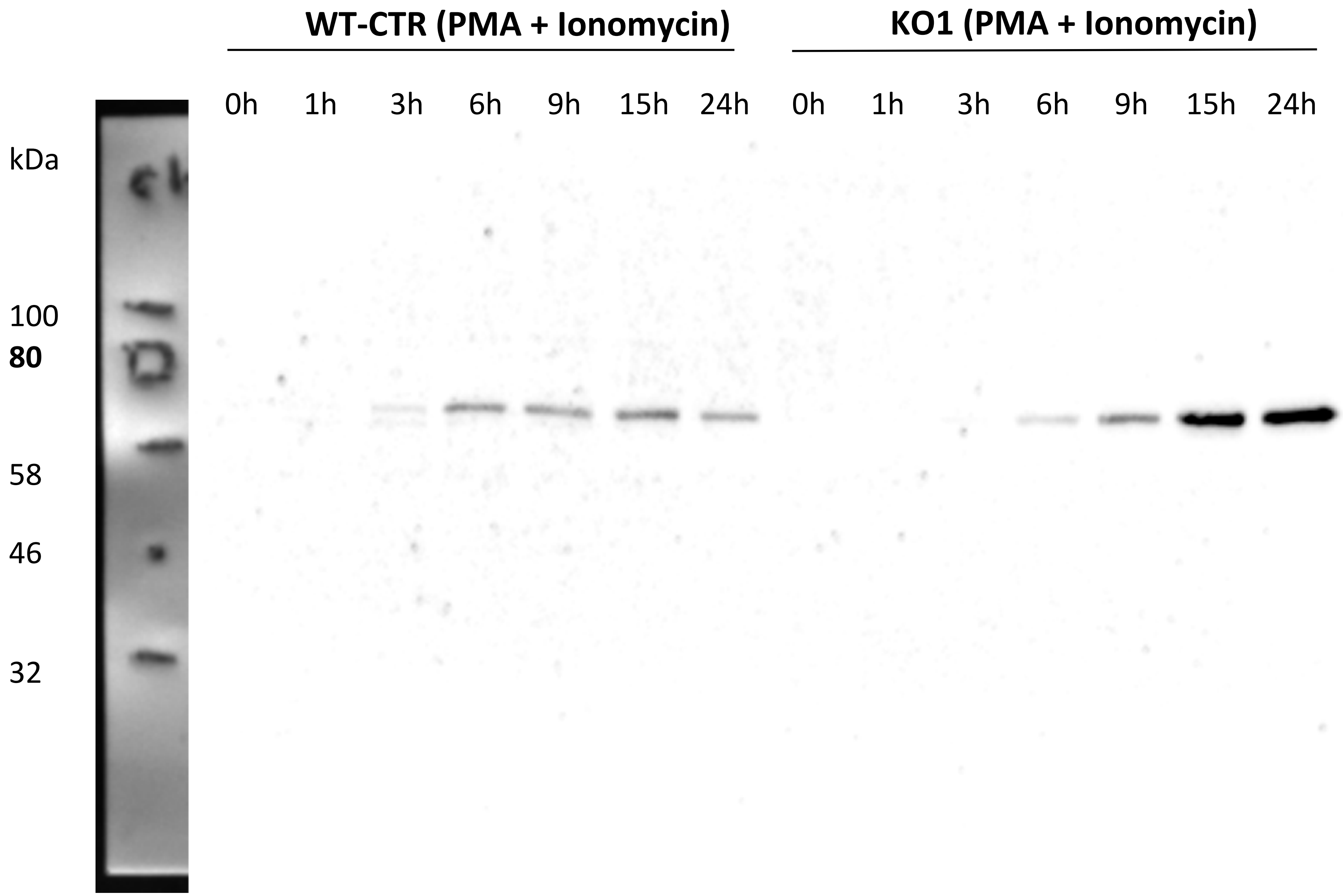

Full images of Western blots for β-actin (Suppl. Fig. 1 h).

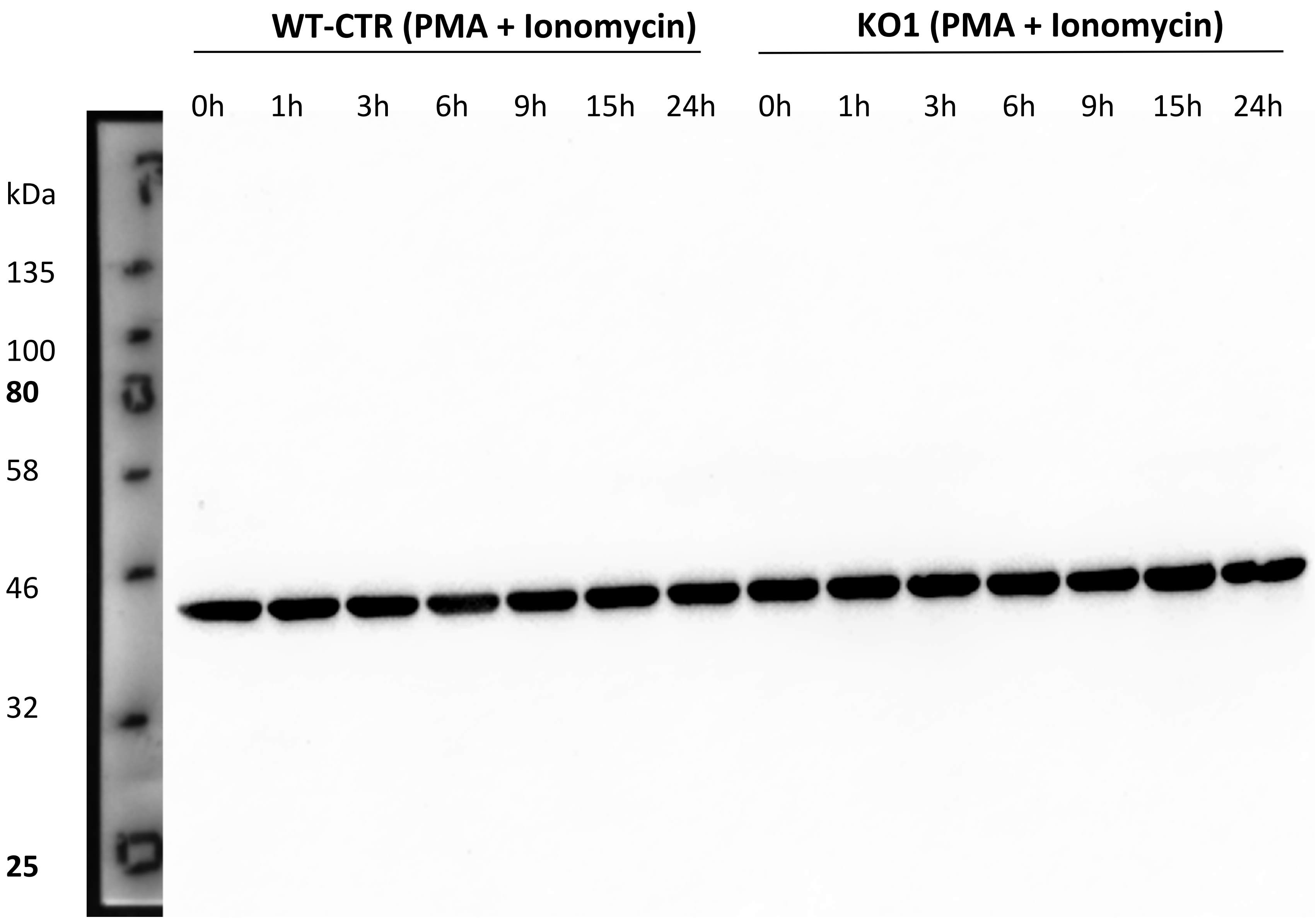

Full images of Western blots for OGlcNac (Suppl. Fig. 2j).

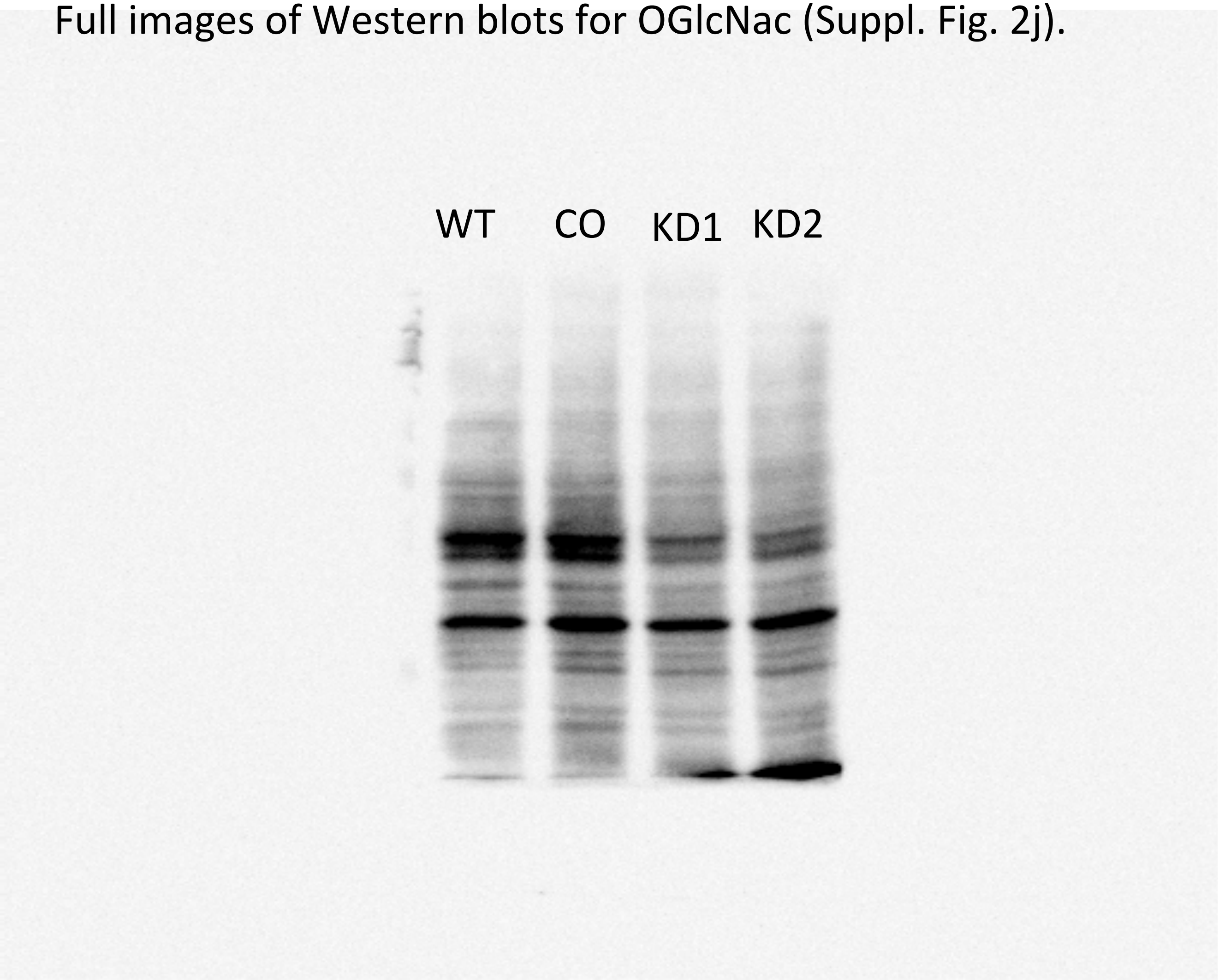

Full images of Western blots for Actin (Suppl. Fig. 2j), size appropriate.

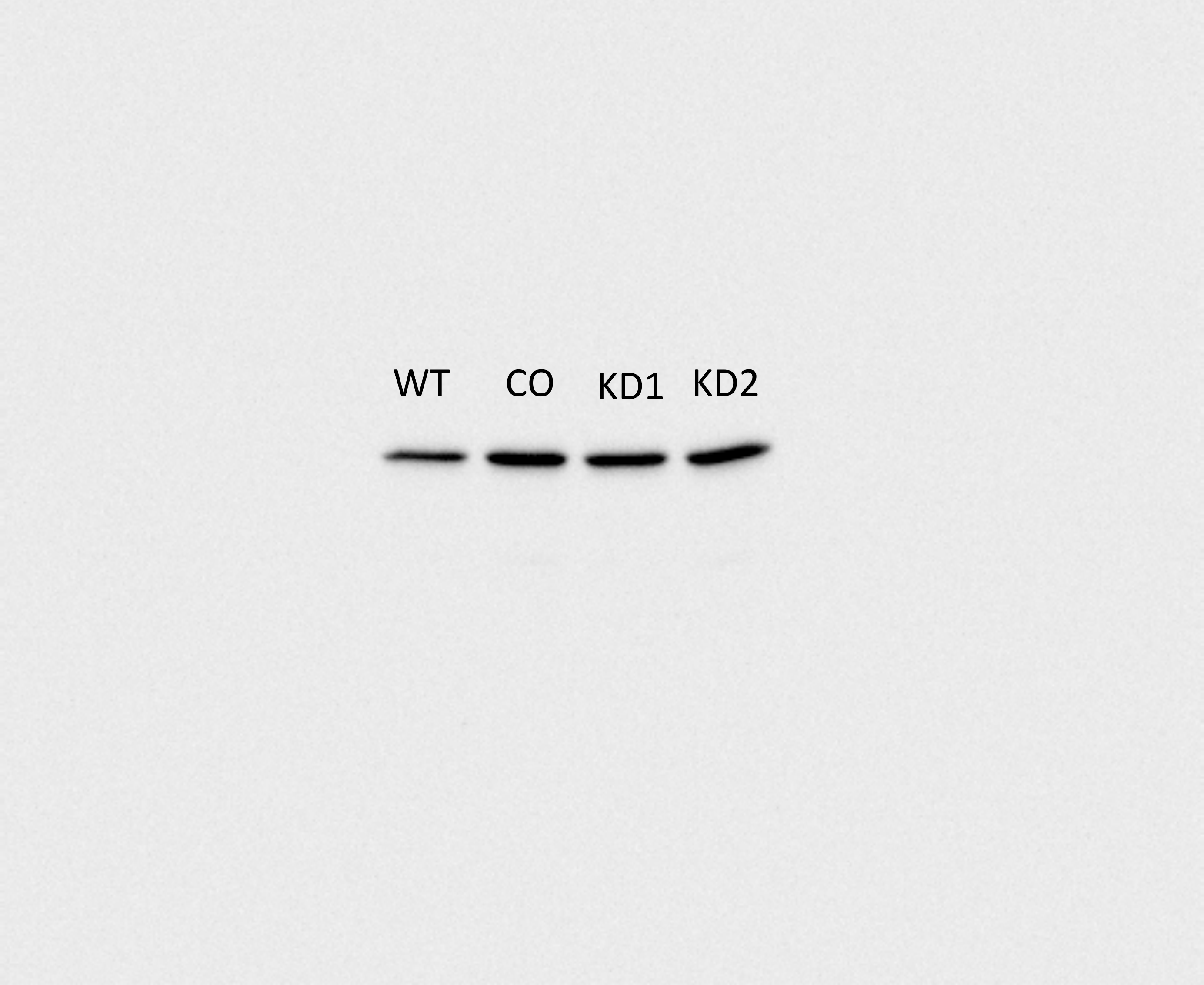

Full images of Western blots for O-GlcNAc residues on proteins (Suppl. Fig. 1j).

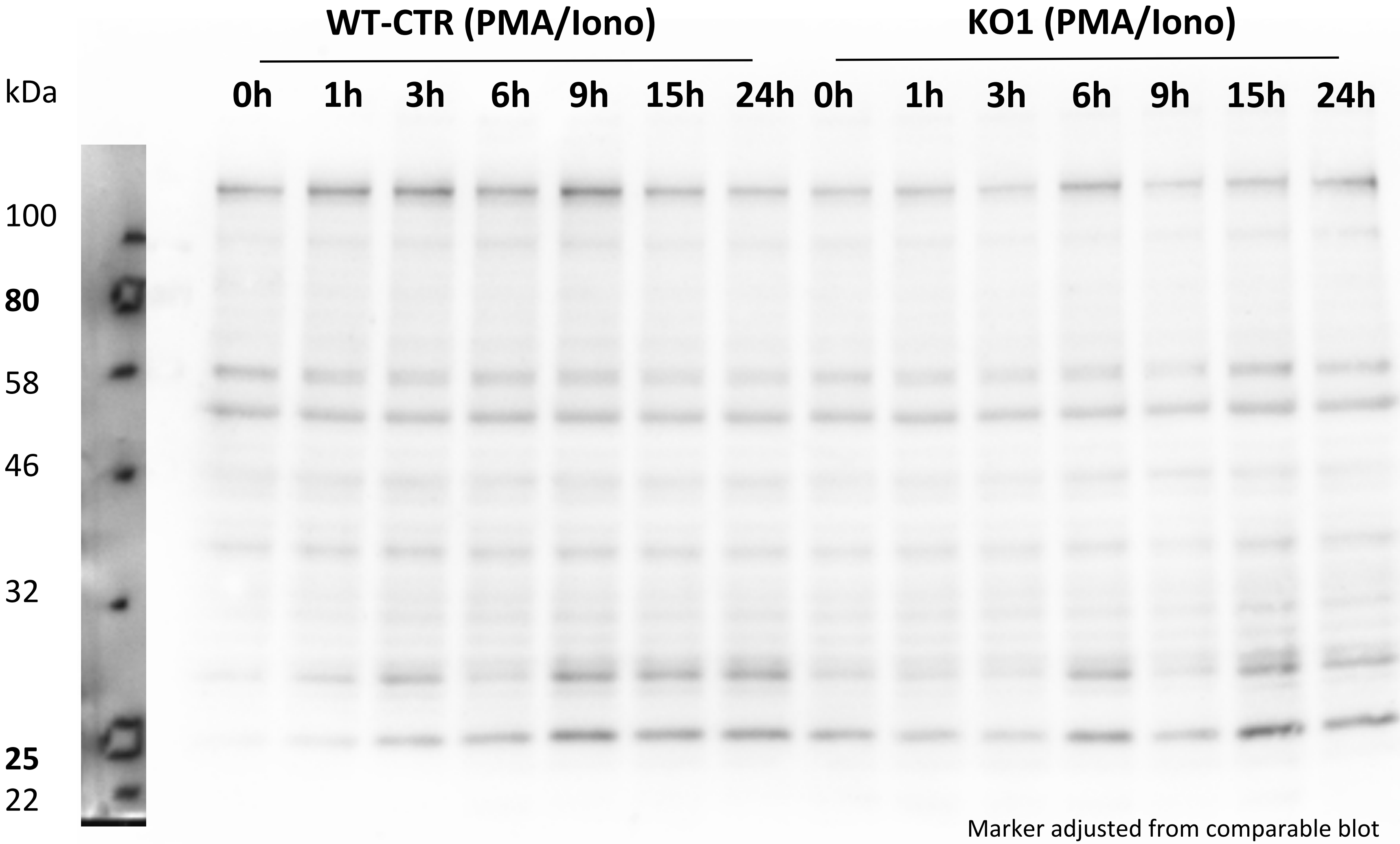

Full images of Western blots for beta Actin (Suppl. Fig. 1j).

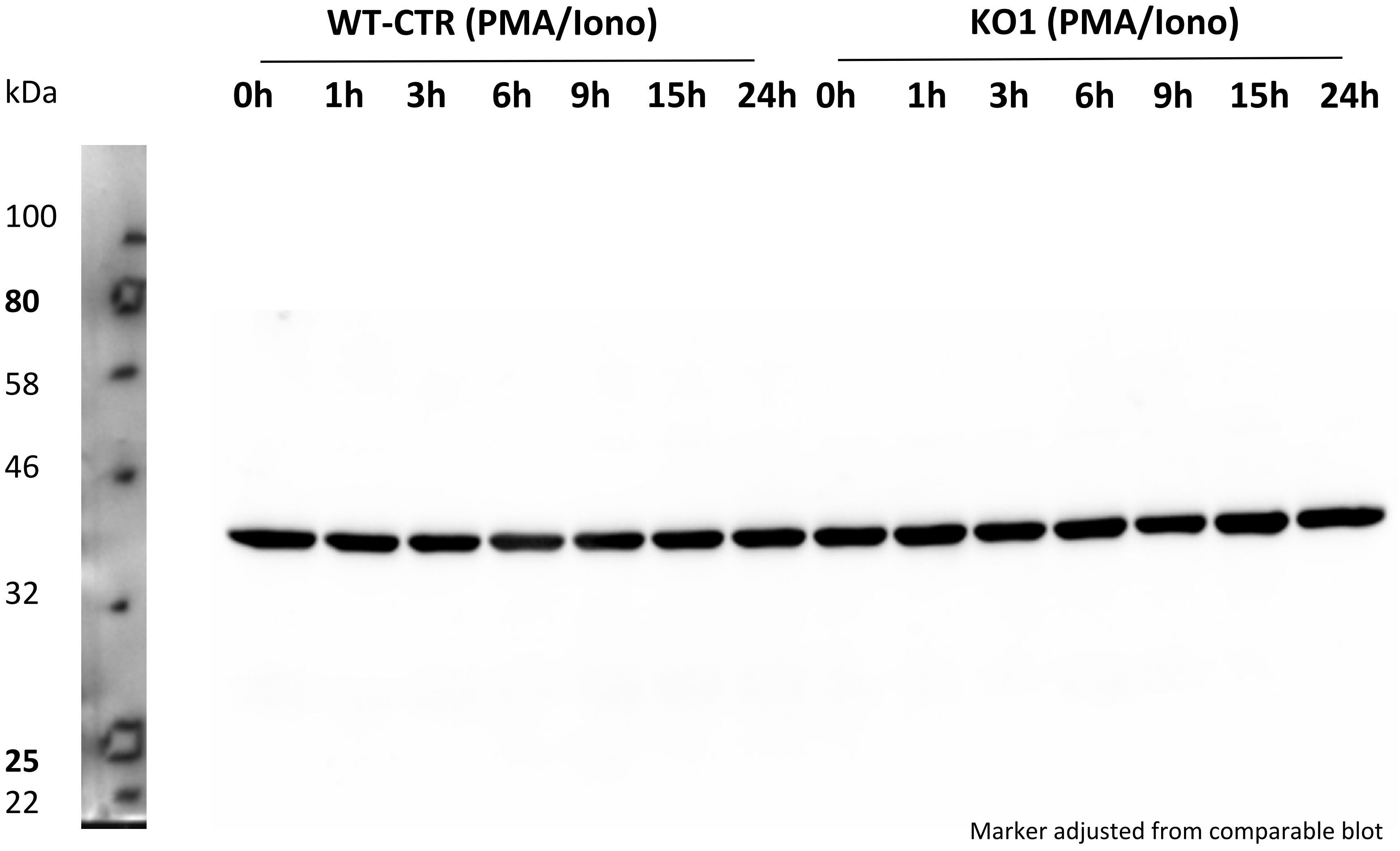

Supplement: Supplementary file 1 — Supplementary information [file 41598_2019_50566_MOESM1_ESM.pdf]
